# Global Changes in the Rat Heart Proteome Induced by Prolonged Morphine Treatment and Withdrawal

**DOI:** 10.1371/journal.pone.0047167

**Published:** 2012-10-09

**Authors:** Zdenka Drastichova, Jitka Skrabalova, Petr Jedelsky, Jan Neckar, Frantisek Kolar, Jiri Novotny

**Affiliations:** 1 Department of Physiology, Faculty of Science, Charles University in Prague, Prague, Czech Republic; 2 Department of Cell Biology, Faculty of Science, Charles University in Prague, Prague, Czech Republic; 3 Department of Developmental Cardiology, Institute of Physiology, Academy of Sciences of the Czech Republic, Prague, Czech Republic; Thomas Jefferson University, United States of America

## Abstract

Morphine belongs among the most commonly used opioids in medical practice due to its strong analgesic effects. However, sustained administration of morphine leads to the development of tolerance and dependence and may cause long-lasting alterations in nervous tissue. Although proteomic approaches enabled to reveal changes in multiple gene expression in the brain as a consequence of morphine treatment, there is lack of information about the effect of this drug on heart tissue. Here we studied the effect of 10-day morphine exposure and subsequent drug withdrawal (3 or 6 days) on the rat heart proteome. Using the iTRAQ technique, we identified 541 proteins in the cytosol, 595 proteins in the plasma membrane-enriched fraction and 538 proteins in the mitochondria-enriched fraction derived from the left ventricles. Altogether, the expression levels of 237 proteins were altered by morphine treatment or withdrawal. The majority of changes (58 proteins) occurred in the cytosol after a 3-day abstinence period. Significant alterations were found in the expression of heat shock proteins (HSP27, α-B crystallin, HSP70, HSP10 and HSP60), whose levels were markedly up-regulated after morphine treatment or withdrawal. Besides that morphine exposure up-regulated MAPK p38 (isoform CRA_b) which is a well-known up-stream mediator of phosphorylation and activation of HSP27 and α-B crystallin. Whereas there were no alterations in the levels of proteins involved in oxidative stress, several changes were determined in the levels of pro- and anti-apoptotic proteins. These data provide a complex view on quantitative changes in the cardiac proteome induced by morphine treatment or withdrawal and demonstrate great sensitivity of this organ to morphine.

## Introduction

Morphine is one of the most effective drugs known for pain-relieving effects and it has been successfully used in medical practice for a long time as a powerful analgesic to treat many kinds of chronic pain [1]–[3]. Morphine exerts its physiological effects through opioid receptors (ORs), which belong to the large family of G protein-coupled receptors (GPCRs) [4]. Because ORs are mainly expressed in the central nervous system [5]–[6], and morphine is a potentially highly addictive substance [7]–[9], a great deal of attention has been paid to studying the impact of morphine and other opioids on nervous tissue.

Chronic administration of morphine was found to induce changes in OR-mediated signaling, which may underlie the development of opioid tolerance and dependence [10]–[11]. Importantly, neuronal changes induced by opioids have been observed to persist for a long time following cessation of drug exposure [12]. A number of studies indicated that morphine affects the expression of genes involved in processes as diverse as cell signaling [13]–[16], transcription [17]–[18], apoptotis [19]–[20] and cytoskeleton assembly [21]. Nowadays, there is increasing evidence to show that morphine can alter protein expression in different brain areas, even following a single dose. Numerous proteomic studies further expanded the list of brain proteins potentially altered by morphine [22]–[31]. Protein changes induced by morphine treatment have also been observed in *in vitro* experiments on the human neuroblastoma SH-SY5Y cell line, CHO (Chinese hamster ovary) epithelial cells [32]–[33] and primary cortical astrocytes [34].

Whereas the pharmacology and function of opioids in the central nervous system have been quite extensively characterized, the actions of these drugs in peripheral tissues are relatively less understood. It has been well established that morphine may exert significant effects on cardiovascular system [35] and it is used for treatment of some types of heart disease [36]. Morphine has also been studied in connection with its potential cardioprotective effects against ischemia-reperfusion injury [37]–[40]. Despite its broad therapeutic application, the current knowledge regarding morphine effects on myocardial protein expression is rather limited. In our previous study, we found that prolonged administration of high doses of morphine to rats up-regulated the expression of some cytoprotective proteins in the left ventricular myocardium, such as ORP150 (hypoxia up-regulated protein1), GRP78 (78 kDa glucose-regulated protein), HSP27 (heat shock protein beta-1) and HSC71 (heat shock cognate 71 kDa protein) [41]. However, information about the possible effect of morphine withdrawal on the expression of myocardial proteins is missing.

Our present work dealing with the impact of prolonged morphine treatment and subsequent withdrawal on the rat heart proteome has been designed to allow extension of previous findings which were based on a rather limited number of protein spots showing alterations in intensity on 2D gels. For this purpose we used a novel proteomic method called iTRAQ (isobaric tag for relative and absolute quantitation). Besides two-dimensional differential in-gel electrophoresis (2D-DIGE) or proteomic methods based on stable isotope labeling (e.g., isotope-coded affinity tag (ICAT) and stable isotope labeling with amino acids in cell culture (SILAC)), the iTRAQ technique has proved to be very suitable especially for comparative studies in which more than two samples should be evaluated in parallel [42]–[43]. This approach was demonstrated to be more sensitive than 2D-DIGE and ICAT [44]. In contrast to the methods using stable isotope labeling, iTRAQ enables all samples to be processed simultaneously, which reduces analysis time [45]. The big advantage of iTRAQ over 2D electrophoresis, which is the most commonly used method in proteomics, lies in the possibility of identifying low abundant proteins [46] as well as integral membrane proteins [47]. Membrane proteins must be solubilized by detergents before 2D electrophoresis, which can be quite a difficult task [48]–[49]. iTRAQ is a s powerful proteomic approach based on usage of four amine specific isobaric reagents which label the primary amines of peptides from four different biological samples. These isobaric mass labels are placed at the N-termini and lysine side chains of peptides and produce abundant MS/MS signature ions at *m/z* 114.1, 115.1, 116.1, and 117.1. Their relative peak areas are determined by the relative proportions of the labeled peptides [43]. Using this proteomic technique we succeeded in identifying 1090 proteins and revealed that both prolonged morphine treatment and withdrawal is accompanied with wide-ranging changes in myocardial proteins involved in different functions.

## Methods

### Animal Model

All animal experiments complied with the Guide for the Care and Use of Laboratory Animals (NIH Publication No. 85–23, revised 1996) and they were performed with the approval of the Animal Care and Use Committee of the Institute of Physiology, Academy of Sciences of the Czech Republic (Protocol #52/2008). Adult male Wistar rats were housed in groups of 3–4 in standard boxes enriched with saw-dust bedding. They were maintained on a 12-h light/dark cycle with ad libitum access to food and water. Control group of rats (C, n = 10) received an intramuscular (i.m.) injection of sterile normal saline and three groups of animals (n = 10 each) were treated with morphine (10 mg/kg/day, i.m. injection for 10 consecutive days). The first group of morphine-exposed animals was sacrificed 24 h (M), the second group 3 days (M_W_-I) and the third group 6 days (M_W_-II) after the last dose to assess the presumed impact of drug withdrawal. After terminating the experiments, hearts were rapidly excised, dissected, snap-frozen in liquid nitrogen and stored at −80°C until use.

### Cardiac Tissue Processing

**Figure 1:**
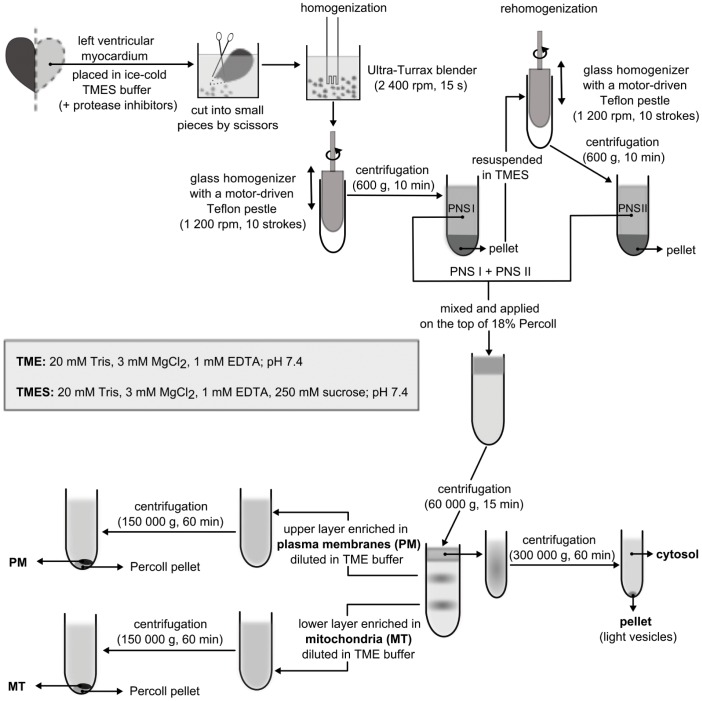
Flow diagram of the fractionation procedure for the rat myocardium. The left ventricles were cut into small pieces with dissecting scissors, homogenized using an Ultra-Turrax homogenizer and subsequently with a motorized glass-Teflon homogenizer. The postnuclear supernatant (PNS I) after low-speed centrifugation was collected and the pellet rehomogenized in a glass-Teflon homogenizer. The resulting postnuclear supernatant (PNS II) was collected, mixed with PNS I and applied on the top of 18% Percoll density gradient. After high-speed centrifugation, the top portion of the gradient mainly containing soluble material was further centrifuged to yield a clear cytosolic fraction. The upper layer enriched in plasma membranes (PM) and the lower layer rich in mitochondria (MT) were separately diluted in TME buffer and spun down to remove the Percoll, which formed a compact glassy pellet at the bottom of the centrifuge tube. The sedimented PM or MT containing material formed a loose white layer on the Percoll pellet and could be easily collected by pipetting. All centrifugation steps were performed at 4°C and tubes were kept on ice during samples collection.

Samples of cardiac tissue were processed basically the same way as described previously [41]. Briefly, the left ventricles were cut into small pieces by scissors and then homogenized on ice using an Ultra-Turrax homogenizer. After subsequent homogenization in a glass-Teflon homogenizer, the suspension was centrifuged at low speed for a short time and the resulting postnuclear supernantant (PNS) collected. The pellet was re-homogenized and the second part of the PNS collected after low-speed centrifugation was mixed with the PNS obtained in the preceding step. The final PNS was fractionated on a Percoll density gradient into three major fractions: cytosol (CS) and plasma membrane (PM)- and mitochondria (MT)-enriched fractions. The whole fractionation procedure is described in detail on a schematic flow diagram (Fig. 1). The final aliquots of cytosol, PM- and MT-enriched fractions were snap-frozen in liquid nitrogen and stored at −80°C until use.

### Proteomic Analysis

For the iTRAQ analysis acetone precipitated protein samples (100 µg each) were dissolved in buffer provided in iTRAQ 4-plex reagent kit (AB Sciex, Foster City, CA) and treated as described by manufacturer. Labeled samples were pooled and precipitated with acetone. Pellet was dissolved in 2 M urea in LC-MS grade water prior to isoelectric focusing on 17 cm immobilized pH gradient strips pH 3–10 (Bio-Rad, Hercules, CA) for 40,000 VHrs. The strip was cut into slices 2 mm wide, which were separately extracted with 50% acetonitrile and 1% trifluoroacetic acid. Supernatant was dilluted 1∶1 by water and used for LC-MALDI.

LC-MALDI analyses were performed on Ultimate 3000 HPLC system (Dionex, Framingham, MA) coupled to Probot micro-fraction collector (Dionex). Tryptic peptides were loaded onto a PepMap 100 C18 RP column (3 µm particle size, 15 cm long, 75 µm internal diameter; Dionex) and separated by a gradient of 5% (v/v) acetonitrile, 0.1% (v/v) trifluoroacetic acid to 80% (v/v) acetonitrile, 0.1% (v/v) trifluoroacetic acid over a period of 60 min. The flow rate was set to 300 nL/min. Eluate was mixed in proportion of 1∶3 with matrix solution (2 mg/mL α-cyano-4-hydroxycinnamic acid in 80% ACN) prior to spotting onto a MALDI target. Spectra were acquired on 4800 Plus MALDI TOF/TOF analyzer (Applied Biosystems/MDS Sciex) equipped with a Nd:YAG laser (355 nm, firing rate 200 Hz). All spots were first measured in MS mode and then up to 12 strongest precursors were selected for MS/MS analysis which was performed with collision energy of 1 kV and operating pressure of collision cell set to 10^–6^ Torr. Tandem mass spectra were processed with 4000 Series Explorer with subtract baseline enabled (peak width 50), Gaussian smoothing enabled (filter width 5), minimum signal to noise 8, local noise window width 250 m/z, minimum peak width at full width half max 2.9 bins, cluster area signal to noise optimization enabled (threshold 15), flag monoisotopic peaks enabled.

Protein identification and quantitation were performed using Protein Pilot 4.0 (AB Sciex). MS/MS spectra were searched against Rattus norvegicus sequences assembly downloaded from GenBank (www.ncbi.nlm.nih.gov/protein) with the following settings: Trypsin digestion (semitryptic peptides allowed), methyl methanethiosulfonate modification of cysteines, iTRAQ 4-plex labeled peptides, instrument 4800, no special factors, default iTRAQ isotope correction settings, quantification, bias correction, background correction, biological modifications and thorough ID parameters selected. Probabilities of modifications were not altered. Detected protein threshold (unused protein score and confidence of results) was set to 2.0 and 99.0% and false discovery rate analysis was enabled. Proteins sharing a set of peptides were grouped automatically with default Pro Group™ Algorithm. Ratios of iTRAQ were calculated with default settings of the Protein Pilot. Protein fold change (iTRAQ ratio for individual protein) was calculated automatically by the Protein Pilot software as a weighted average of Log iTRAQ ratios determined for individual peptides belonging to the particular protein after background subtraction. To estimate the false discovery rate (FDR) a decoy database search was performed. For each protein ratio the Protein Pilot reported p-value and EF (error factor). To be considered as differentially expressed, individual proteins had to fulfill the following statistical criteria: P value<0.05, EF<1.5 and average iTRAQ ratio>2.

### Electrophoresis and Western Blotting

Different preparations of myocardial fractions were solubilized in Laemmli sample buffer and proteins separated by standard SDS-PAGE. After electrotransfer onto nitrocellulose membranes, selected proteins were detected on Western blots using appropriate antibodies. Immunochemically reactive proteins were visualized by conventional enhanced chemiluminiscence detection (Pierce Biotechnology, Rockford, IL, USA). The Western blots were scanned and evaluated quantitatively by means of densitometry using ImageQuant™ TL software (GE Healthcare, Chalfont St. Giles, UK).

## Results and Discussion

### Identification of Proteins in Myocardial Fractions

The left ventricles isolated from control (C), morphine-treated (M) and morphine-withdrawn (M_W_-I and M_W_-II) rats were subfractionated into cytosolic (CS), plasma membrane (PM)- and mitochondria (MT)-enriched fractions (Fig. 1). Enrichment of these fractions with typical cytosolic, plasma membrane-bound or mitochondrial proteins was demonstrated previously [41]. Proteins present in each preparation were digested with trypsin and peptide populations in the individual fractions were labeled with distinct iTRAQ reagents. The corresponding peptide populations from samples of all four experimental groups were then combined for further analyses.

**Figure 2:**
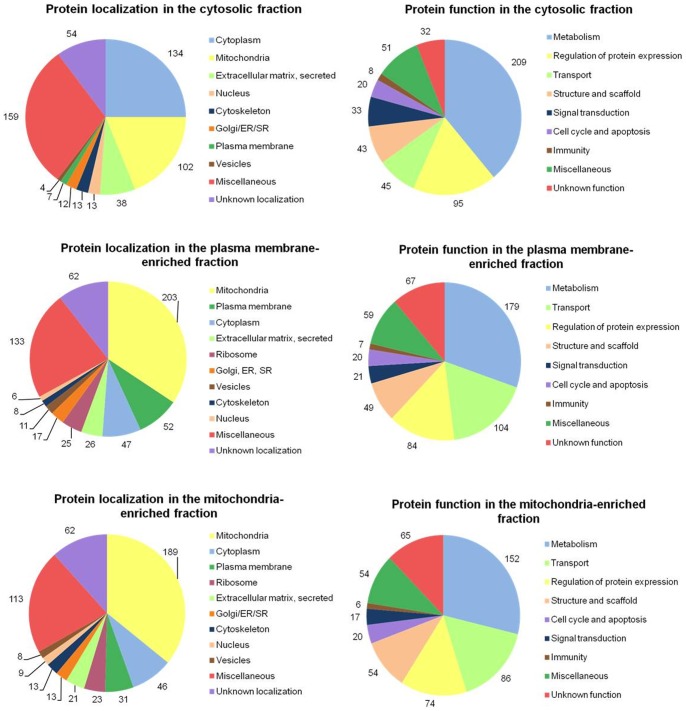
Classification of proteins identified in the left ventricular myocardium according to their subcellular localization and function. The proteins of each fraction (CT, PM and MT) isolated from hearts of control (C), morphine-treated (M), and morphine-withdrawn (M_W_-I and M_W_-II) rats were labeled by isobaric reagents provided in iTRAQ 4-plex reagent kit. After LC-MALDI analyses performed on Ultimate 3000 HPLC system and acquisition of spectra on 4800 Plus MALDI TOF/TOF analyzer, proteins were identified and quantified using Protein Pilot 4.0. Localization and function of the proteins were assigned on the basis of current annotations in the Swiss-Prot database. Sections of the pie charts represent the proportion of proteins found within each functional category.

Using this approach, 541 proteins were identified in the cytosol, 595 proteins in the PM-enriched fraction and 538 proteins in the MT-enriched fraction. Some of these proteins were found in two or all three fractions simultaneously. Altogether, 1090 different proteins were identified in all three fractions prepared from rat heart. The proteins detected in each fraction were divided according to their localization or function and their distributions depicted in the form of pie charts (Fig. 2).

Almost three-quarters of all proteins determined in the cytosol were specific only for this fraction and were not present in the other two fractions or at least they did not occur there in detectable amounts. On the other hand, around half of the proteins found in the PM-enriched fraction were present also in the MT-enriched fraction, and *vice versa*. However, relatively extensive cross-contamination of these two fractions did not preclude the assessment of many typical plasma membrane or mitochondrial proteins in these preparations. Although the proteins found in both PM- and MT-enriched fractions most often belonged to the mitochondrial compartment, the PM-enriched fraction harbored a far greater number of PM-bound proteins than MT-enriched fraction and it was enriched in such PM proteins as caveolin 1, caveolin 2 and caveolin 3, flotillin 1, Gβ_2_ subunit, AP-2 α_1_ subunit and AP-2 σ_1_ subunit. Whereas four different subunits of spectrin (erythrocytic spectrin α_1_, erythroid spectrin β, spectrin β_2_, α-fodrin (spectrin α_2_)), which represents an important scaffold protein crosslinking the PM with actin skeleton, were detected only in the PM-enriched fraction, non-erythrocytic spectrin β_1_ was found only in the MT-enriched fraction. Some of the PM-bound proteins (Na,K-ATPase α_1_ subunit precursor, Na,K-ATPase α_2_ subunit precursor, Gα_i2_ subunit) were detected in both membrane fractions and others (clathrin heavy chain) in the PM-enriched fraction and cytosol.

Protein classification according to their functional categories revealed that the largest group in each of the three fractions comprised the proteins participating in metabolic processes (Fig. 2). This finding is in agreement with some previous reports indicating a high proportion of metabolic proteins in the rat cardiac proteome [50]–[51]. Interestingly, both membrane fractions (PM and MT) contained approximately twice more transport proteins than the cytosolic fraction.

**Figure 3:**
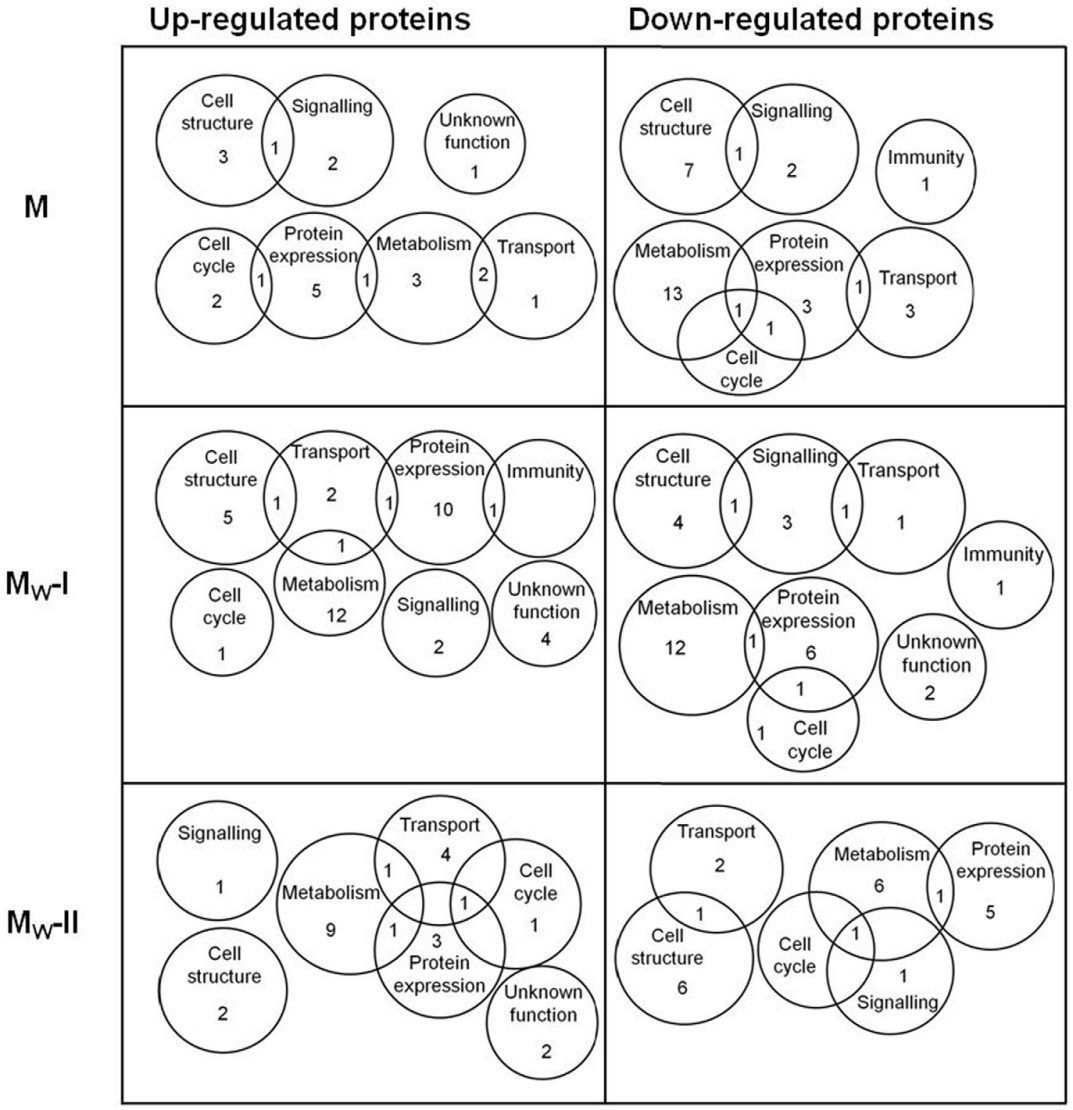
Venn diagrams showing the distribution of myocardial proteins in the cytosolic fraction altered by morphine treatment and withdrawal. Numbers in the individual circles or sectors refer to the number of altered proteins which were determined in the cytosolic fraction isolated from the left ventricles of morphine-treated (M) and morphine-withdrawn for 3 days (M_W_-I) or 6 days (M_W_-II) rats. These proteins were arranged according to their function into 16 groups.

**Figure 4:**
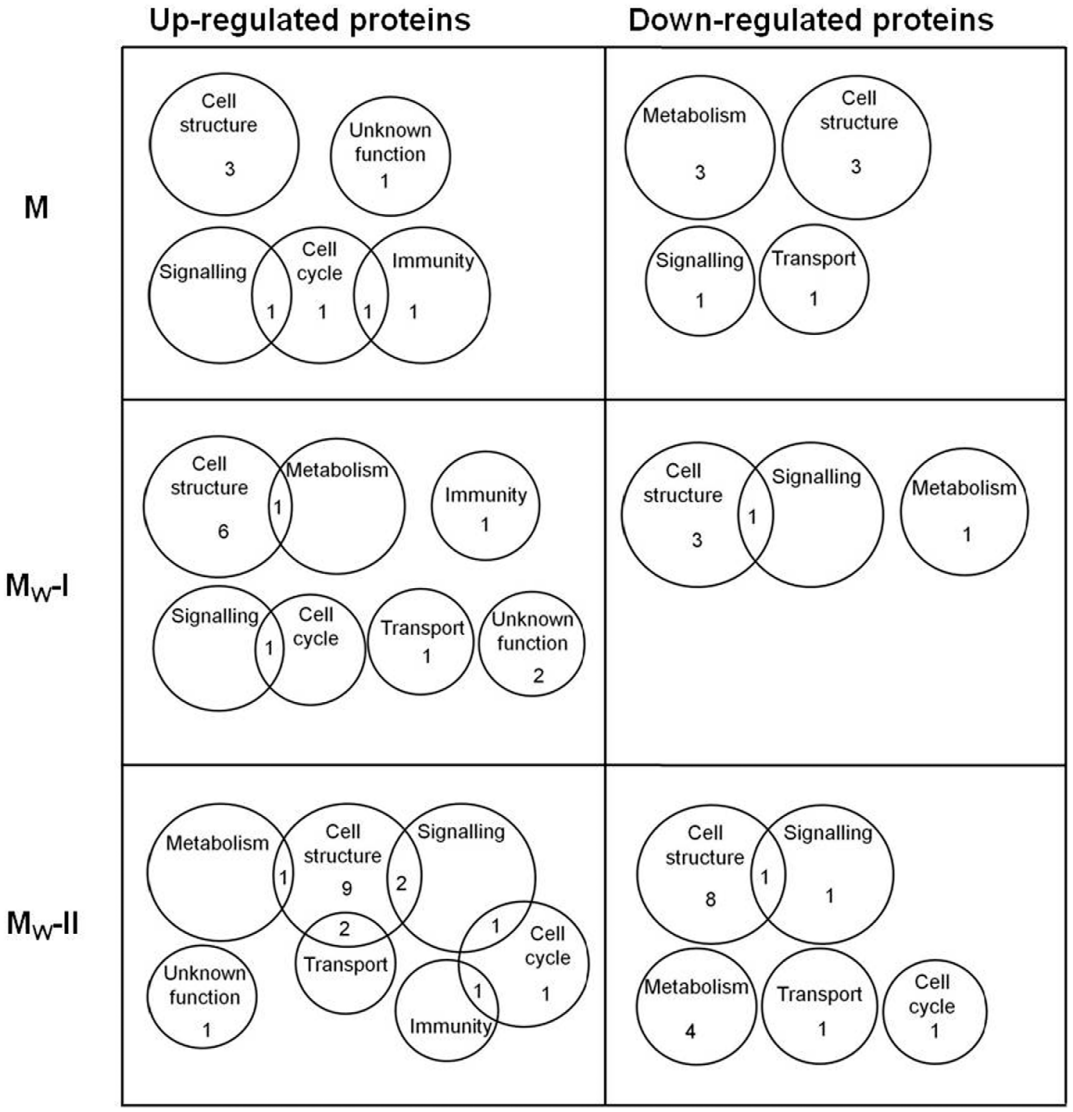
Venn diagrams showing the distribution of myocardial proteins in the plasma membrane-enriched fraction altered by morphine treatment and withdrawal. Numbers in the individual circles or sectors refer to the number of altered proteins which were determined in the PM-enriched fraction isolated from the left ventricles of morphine-treated (M) and morphine-withdrawn for 3 days (M_W_-I) or 6 days (M_W_-II) rats. These proteins were arranged according to their function into 16 groups.

**Figure 5:**
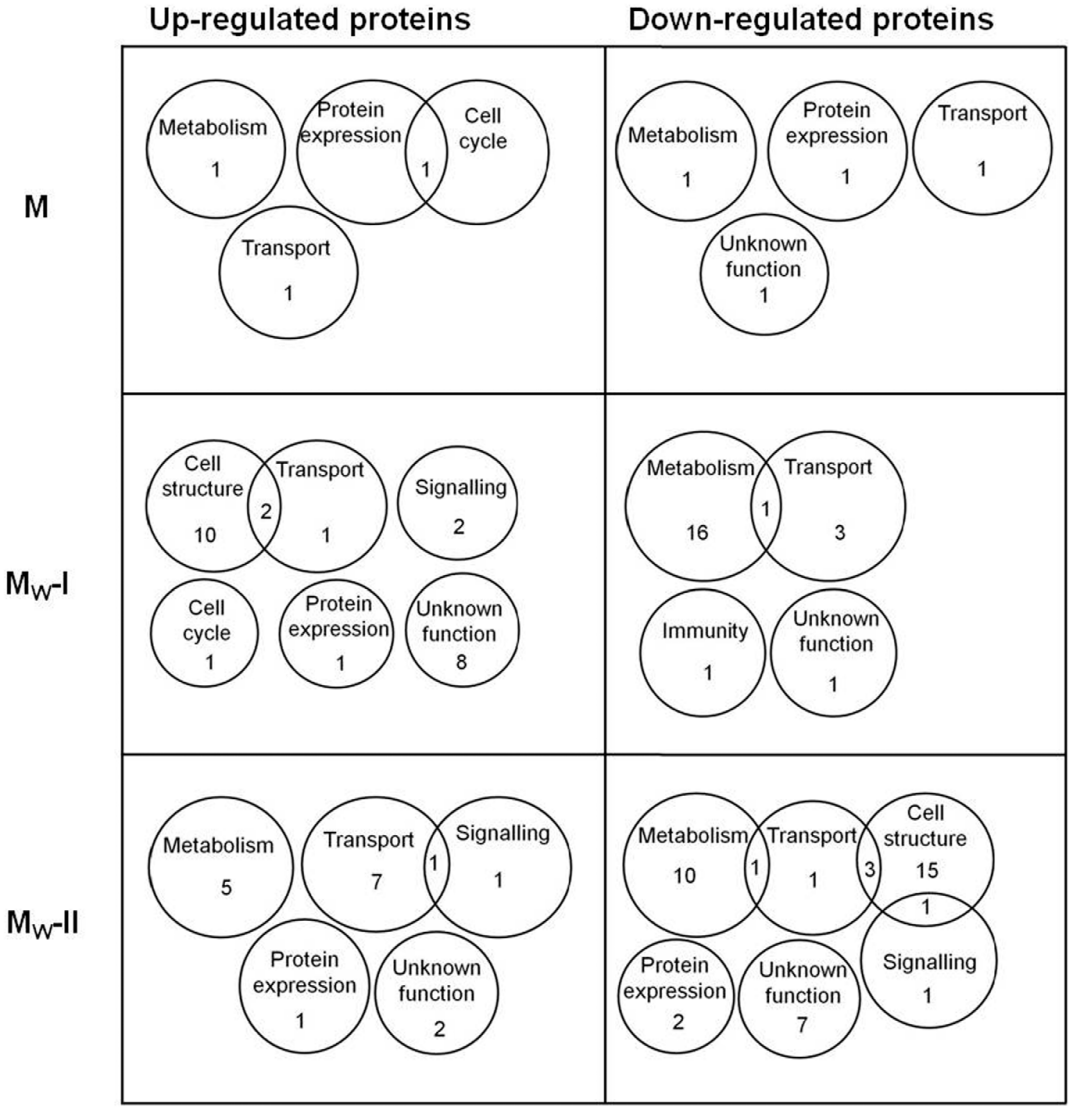
Venn diagrams showing the distribution of myocardial proteins in the mitochondria-enriched fraction altered by morphine treatment and withdrawal. Numbers in the individual circles or sectors refer to the number of altered proteins which were determined in the MT-enriched fraction isolated from the left ventricles of morphine-treated (M) and morphine-withdrawn for 3 days (M_W_-I) or 6 days (M_W_-II) rats. These proteins were arranged according to their function into 16 groups.

Proteins were considered as up- or down-regulated only if at least two-fold change was recorded in the protein abundance. Levels of 237 proteins were found to be altered in myocardial samples from morphine-exposed or withdrawn rats as compared with the corresponding controls. In order to describe synoptically which cell functions might have been affected by morphine treatment or withdrawal, distribution of altered proteins according to their function has been depicted using Venn diagrams for each group (M, M_W_-I and M_W_-II) and fraction (CS, PM and MT) (Fig. 3, 4 and 5).

**Figure 6:**
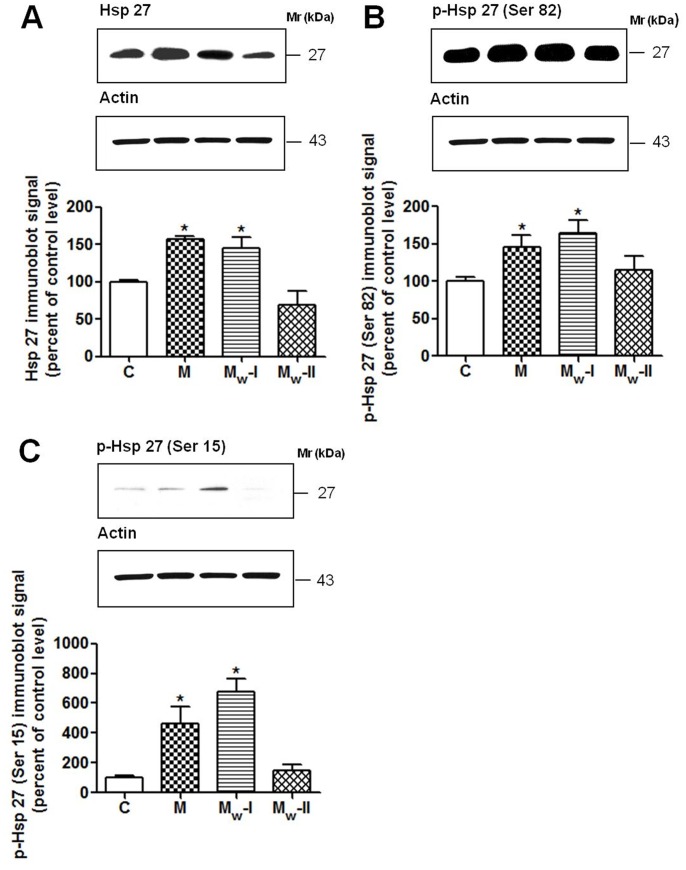
Immunoblot analyses of the unphosphorylated and phosphorylated forms of HSP27. Cytosolic proteins (20 µg) isolated from the left ventricular myocardium of control (C), morphine-treated (M), and morphine-withdrawn for 3 days (M_W_-I) or 6 days (M_W_-II) rats were separated on 15% polyacrylamide gels by SDS-PAGE and electrotransferred onto the nitrocellulose membrane. Specific primary antibodies were used for the detection of unphosphorylated HSP27 (A) and HSP27 phosphorylated at Ser82 (B) and Ser15 (C). Actin was used as a loading control and the relative levels of individual forms of HSP27 (HSP27, p-HSP27-Ser82 or p-HSP27-Ser15) after normalization were expressed as a percentage of the corresponding control level. Data represent the mean±S.E.M. of three separate experiments; *p<0.05 vs control.

The proteins whose levels were altered by morphine treatment or withdrawal are listed in Table S1 (see Supplement). Whereas administration of morphine for 10 days changed the expression of 72 myocardial proteins, subsequent 3- or 6-day drug abstinence was accompanied by alteration in 105 or 60 proteins, respectively. Proteins with significantly altered expression levels were unevenly distributed between all three myocardial fractions: 121 proteins were found in the cytosol, 30 proteins in the PM-enriched fraction, 69 proteins in the MT-enriched fraction and 17 altered proteins occurred in two or three fractions simultaneously. The majority of proteins with altered expression were found in the cytosolic compartment and most changes (58) appeared 3 days after morphine withdrawal. Hence, drug withdrawal was apparently associated with more profound alterations in the rat cardiac proteome than sustained morphine treatment alone. Most of the altered proteins included those involved in the metabolism, structural support and regulation of protein expression. All the identified proteins whose levels were not significantly altered are listed in Table S2 (see Supplement).

### Proteins Involved in Signal Transduction

All detected proteins can be arranged into several groups according to their function or structural homology. Some proteins such as ribosomal proteins or small G proteins (members of Rab family, RhoA, Rac2, RAP1A, Ran or Ral-A) were not altered at all. However, it does not mean that the fate of small G proteins cannot be influenced by morphine treatment or withdrawal. Interestingly, the level of GDP dissociation inhibitor 2 (GDI-2) which regulates the translocation of GDP-bound Rabs from and to the membrane [52] decreased 2.3 times following a 3-day morphine abstinence period. It can be speculated that membrane release and activation of Rab proteins might have been affected under these conditions [53].

As mentioned above, mass spectrometry analysis allowed the identification of some subunits of trimeric G proteins and also some other proteins, which can be involved in signal transduction mediated by opioid receptors. We did not reveal any alterations in the amount of Gα_i2_ protein, Gβ_2_ protein, GNAS complex locus Xlas (fragment of the long isoform of Gα_s_ protein) or subunits of c-AMP dependent protein kinases (PKA catalytic subunit α, PKA I regulatory subunit α, PKA II regulatory subunit α). Interestingly, 6-day morphine withdrawal resulted in up-regulation of caveolin 1 (2.0-fold) and flotillin 1 (2.1-fold), which could imply a possible reorganization of the plasma membrane and increased formation of caveolae and lipid rafts [54].

Although there was no detectable amount of protein kinase C isoforms, some proteins with protein kinase activity or proteins engaged in PKC signaling, such as PKC substrates or binding proteins (PKC and casein kinase substrate in neurons protein 2, PKC and casein kinase substrate in neurons protein 3, PKC δ-binding protein, PKC substrate 80K-H), were identified mainly in the PM-enriched fraction. However, the myocardial expression of these proteins was not affected by morphine treatment. Similarly, no changes were found in the levels of the identified protein phosphatases (PP1 catalytic subunit α, PP1 catalytic subunit β, PP2A catalytic subunit β, PP2A regulatory subunit A α, PP2A regulatory subunit B α, protein phosphatase T, protein tyrosine phosphatase-like member a, protein tyrosine phosphatase non-receptor type 11, protein-tyrosine phosphatase mitochondrial 1, low molecular weight protein tyrosine phosphatase isoform A).

Interestingly, phospholipase C δ1 (PLC-δ1) was found to be up-regulated (3.2-fold) in the cytosolic fraction after morphine treatment, and 3 days of abstinence resulted in its marked down-regulation (10.7-fold). PLC-δ isoforms were shown to differ from the other PLC isoforms because they are not activated by heterotrimeric G proteins or protein phosphorylation cascades [55]. It was proposed that this enzyme is activated by a rise in the concentration of free cytosolic calcium generated by PLC-β and further amplifies Ca^2+^ signals initiated by activation of PLC-β, -γ and -ε isoforms [56]. It also can be activated by transglutaminase II (TGII or Gα_h_), which is an atypical G protein with GTPase and transglutaminase activities [57]. PLC-δ1 was shown to be an effector and GEF protein for TGII in the α_1B_- and α_1D_-adrenergic receptor pathways [55], [57]. Our analyses allowed us to identify some proteins which can interact with PLC-δ1 such as importin β1 [58], isoform of Ral protein and calmodulins [56] or proteins which can inhibit PLC-δ1 activity such as RhoA [59], but the expression levels of none of these proteins were affected neither by morphine treatment nor by subsequent drug withdrawal. Interestingly, in our previous study based on 2D mapping we found a 2.3-fold increase in the amount of phosphatidylinositol transfer protein α (PITPα) in the cytosolic fraction after prolonged administration of morphine to rats [41]. Because previous research has shown that the presence of PITPα may enhance the activity of PLC-δ1 in HL-60 cells [60], it is highly likely that not only morphine treatment led to up-regulation of PLC-δ1 but also might have enhanced its activity. It was previously reported that PLC-δ1 has an important role in the protection mediated by tumor necrosis factor (TNF) receptor against adriamycin-induced cardiotoxicity [61]. The present finding of a 3.2-fold increase of PLC-δ1 in the cytosolic fraction of myocardial samples from morphine-treated rats suggests that this enzyme could also participate in the molecular mechanism of cardioprotective action of this drug. However, only future research aimed at untangling the molecular mechanism of cardioprotection conferred by morphine can prove or disprove this speculative hypothesis. Apart from PLC-δ1, lysophospholipase 1 and phospholipase A2 activating protein were also identified by iTRAQ analysis but myocardial levels of these proteins were not altered by morphine.

It is known that the acute or chronic morphine treatment can cause the elevation of free cytosolic calcium concentration via PLC-β [62]–[64]. One might therefore speculate that the levels of some calcium binding proteins or proteins involved in calcium handling, transport and signaling could possibly be influenced by morphine treatment. Nevertheless, only histidine-rich calcium protein identified in the PM-enriched fraction was significantly up-regulated (3- or 3.9-fold, respectively) after 3- or 6-day drug withdrawal. Interestingly, its level was not changed in the other two fractions prepared from samples derived from morphine-exposed animals (M, M_W_-I, M_W_-II), compared to the corresponding controls. The other proteins in this group (calcium binding protein, calcium regulated heat stable protein 1, calcyclin, calmodulin 2, calnexin, calsequestrin 1, calsequestrin 2, calpain small subunit 1, sarcalumenin, sarcoplasmic reticulum Ca^2+^-ATPase, vasopressin-activated calcium-binding mobilizing receptor protein) were not altered at all. These results suggest that if Ca^2+^ signaling pathways were affected by morphine, this effect need not necessarily be accompanied by marked quantitative changes of Ca^2+^ binding proteins.

### Proteins Involved in Apoptosis and Oxidative Stress

A number of studies dealing with morphine effects on cell viability, apoptosis and oxidative stress have been published during the past decade. It was reported that morphine treatment resulted in apoptosis in macrophages [65]–[66], SH-SY5Y [67] or microglia and neurons [68]–[69]. On the other hand, protective effects of morphine were observed in macrophages [70], astrocytes [69], [71] or heart [37]–[38], [64]. Morphine has also been shown to enhance the generation of reactive oxygen species (ROS) in macrophages [65] and SH-SY5Y [67]. Hence, it seems that morphine can cause either cell apoptosis or protection depending on the cell type and experimental context. Therefore, it is worth paying attention to potential changes of proteins involved in apoptosis, cellular protection and ROS generation.

Many proteins involved in cellular protection/apoptosis, which were detected in our present study, were not altered after morphine treatment or withdrawal, e.g. proteins that can induce apoptosis and/or activate caspases (apoptosis-inducing factor, diablo, C9 protein, B-cell associated protein 31, BCL2/adenovirus E1B 19 kDa-interacting protein 3, endonuclease G, Gβ_2_-like 1, reticulon-3, VDAC1, SP120), proteins that protect cells against apoptosis (CHIP28k, mitochondrial inner membrane protein, glutathione peroxidase 4 isoform A precursor, mitochondrial protein 18 kDa, prohibitin, protein DJ-1, vanin 1), proteins engaged in release of cytochrome *c* from mitochondria into the cytoplasm (Clu protein, growth hormone-inducible transmembrane protein, mitochondrial fission 1 protein, presenilin associated rhomboid like) or proteins conferring protection against release of cytochrome *c* (mitochondrial OPA1, optic atrophy 1 homolog, sulphated glycoprotein 2).

We determined four proteins possibly involved in the induction of apoptosis, expression of which was affected by morphine. Programmed cell death 5 protein was up-regulated (2.1 fold) in the cytosolic fraction after morphine treatment, dynamin 1 like was up-regulated (2.6-fold) in the cytosolic fraction after a 3-day drug abstinence period, programmed cell death 6 interacting protein was up-regulated (2.6-fold) in the cytosolic fraction after 6-day abstinence and lactate dehydrogenase A was down-regulated (3.4-fold) in the cytosolic fraction after 6-day abstinence. Interestingly, the level of the latter protein was not changed in the PM- and MT-enriched fractions. Another four proteins were found to be altered after morphine exposure or withdrawal that can play a role in protection of cells against apoptosis. Cystatin B was down-regulated (2.2-fold) and Bcl2-associated anthanogene 3 up-regulated (2.4-fold) in the cytosolic fraction after morphine treatment, prothymosin α was down-regulated (4.9-fold) in the cytosolic fraction after 3 days of drug abstinence, acetyl-CoA acyltransferase 2 was down-regulated (3.2-fold) in the MT-enriched fraction after 6 days of abstinence (its level was unchanged in the PM-enriched fraction and cytosol). These results indicate that a certain imbalance between pro- and anti-apoptotic proteins may arise in the myocardium during morphine treatment or withdrawal. However, up-regulation of programmed cell death 5 protein in the cytosol does not necessarily mean the induction of apoptosis because this protein is translocated from the cytosol to the nucleus during the early stage of apoptosis [72].

It seems that if chronic morphine treatment induced apoptosis, it would not be mediated by the formation of pores in the mitochondrial membrane and release of cytochrome *c*. OPA1 protein and mitochondrial inner membrane protein, which control the shape of mitochondrial cristae and thus may regulate cytochrome c redistribution [73]–[75], were not affected by morphine treatment or withdrawal. The expression of another protein involved in mitochondrial fusion, mitofusin-1 [73]–[74], was also not changed. Mitochondrial morphology thus did not seem to be affected by morphine. Moreover, no release of cytochrome *c* from mitochondria to the cytoplasm was observed after morphine treatment or withdrawal; the levels of cytochrome *c* detected in all three fractions were not altered.

Calpain-dependent proteolysis can contribute to cell death by cleavage of pro-apoptotic Bid [76]. There are two different major isoforms of calpain, μ-calpain (calpain 1) and m-calpain (calpain 2), which contain a large 80 kDa catalytic subunit and a common small 30 kDa regulatory subunit [77]. They are activated by elevation of intracellular calcium and their proteolytic activity is regulated by specific inhibitor calpastatin [77]. In our present study, a small regulatory subunit of calpain, m-calpain and calpastatin were identified. Nevertheless, the levels of these proteins were not altered by morphine treatment or withdrawal. In addition, expression of the majority of caspase and calpain substrates (α-fodrin, actin, protein phosphatase 2A, vimentin, lamin A, Ca^2+^-ATPase, Gα protein, PKA, ryanodine receptors, talin, tropomyosin, tubulins, vimentin, vinculin) [78]–[79] identified in our study were also not altered. Only tau and troponin T were down-regulated (17.0-fold and 2.8-fold, respectively) after 3-day drug abstinence. These results indicate that calpain and caspase pathways were not significantly activated by morphine treatment or withdrawal.

Similarly, there were no significant changes in the expression levels of proteins involved in modulation of oxidative stress, such as superoxide dismutase 1, superoxide dismutase 2, glutathione peroxidase, glutathione peroxidase 4 isoform A precursor, glutaredoxin 3, glutaredoxin 5, peroxiredoxin 2, peroxiredoxin 3, peroxiredoxin 5, peroxiredoxin 6, antioxidant enzyme B166, catalase, protein DJ-1, thioredoxin 1, thioredoxin like protein 1, thioredoxin domain containing 17, thioredoxin reductase 2, protein disulfide isomerise A6, CDGSH iron sulfur domain containing protein 1, dihydrolipoyl dehydrogenase mitochondrial.

### HSPs

Some members of the family of heat shock proteins (HSPs) were markedly affected by morphine treatment or withdrawal. Alterations in HSPs expression were observed mainly in the cytosolic fraction and less in the MT-enriched fraction. No changes were found in the PM-enriched fraction. Heat shock 10 kDa protein 1 (HSP10) was up-regulated (2.1-fold) in the cytosolic fraction after morphine treatment. Interestingly, this protein was up-regulated (2.3-fold) in the cytosol and down-regulated in the MT-enriched fraction (2.1-fold) after 6-day drug abstinence. These results suggest that HSP10 is affected by morphine and that subsequent withdrawal can cause its redistribution from the cytoplasm to membrane compartments.

Heat shock 60 kDa protein (HSP60) was significantly up-regulated only in the cytosolic fraction (and not in both membrane fractions) after morphine treatment (2.0-fold) and a 6-day morphine abstinence (2.0-fold). Similarly to HSP10, a slight redistribution of HSP60 to the MT-enriched fraction (1.8-fold increase) was found after 6-day morphine abstinence, which may imply that both proteins may undergo a common fate. The anti-apoptotic effect of both HSP10 and HSP60 has been well established in several studies in which over-expression of these proteins protected myocytes and H9C2 cells against ischemic injury [80]–[81]. In addition, HSP60 have been shown to interact with Bax/Bak proteins, thus providing protection against apoptosis [82].

Heat shock 70 kDa protein 1A/1B (HSP70) was markedly up-regulated in the cytosolic fraction (15.9-fold) as well as MT-enriched fraction (2.3-fold) after morphine treatment. This protein was also up-regulated (6.8-fold) in the cytosolic fraction after 6 days of morphine abstinence. Importantly, it has been demonstrated that over-expression of HSP70 protects the myocardium from ischemic injury [83].

Two members of the family of small heat shock proteins with cell protective effects, heat shock protein 27 kDa protein 1 (HSP27, HSPB1) and α-B crystallin [84]–[85], were also found to be altered. Both HSP27 and α-B crystallin were up-regulated (2.6- and 2.1-fold, respectively) in the cytosolic fraction after morphine treatment. α-B crystallin was up-regulated (2.1-fold) in the cytosolic fraction also after a 6-day drug abstinence. It was shown that these proteins can be phosphorylated and activated *via* the MAPK p38 (MAPK14)/MAPKAP-2 pathway [86]–[87]. Interestingly, in our study MAPK14 (isoform CRA_b) was found to be up-regulated (2.5-fold) in the cytosolic fraction after morphine treatment. Therefore, it is plausible to assume that cardioprotection induced by morphine may occur at least partly via the MAPK p38/MAPKAP-2/HSP27 or α-B crystalline pathway.

In order to determine whether HSP27 could be activated by morphine treatment or withdrawal, its phosphorylation state was assessed by Western blotting using specific antibodies against phosphorylated amino acids Ser82 and Ser15 of HSP27 (Fig. 6). Both these sites can be phosphorylated by MAPKAP-2 and the major site of phosphorylation of this protein is Ser82 [88]. The increased expression levels of HSP27 in samples from morphine treated animals were verified by Western blotting (Fig. 6A). The amount of HSP27 phosphorylated on Ser15 and, to a lesser extent, on Ser82 was also increased (Fig. 6B,C). Although HSP27 was phosphorylated at both phosphorylation sites after morphine treatment, 3-day drug abstinence further increased its phosphorylation. By contrast, after 6 days of abstinence HSP27 phosphorylation returned to normal values. These results partly support some recently published findings. Two recent studies have reported that naloxone-precipitated morphine withdrawal induced activation of HSP27 by phosphorylation at Ser15 but not at Ser82 [89]–[90]. Moreover, although chronic morphine treatment led to increased expression of HSP27, enhanced phosphorylation and activation of this protein was not observed [89]–[90]. The partial discrepancy between these and our present observations may be explained by different experimental conditions. Nevertheless, it can be concluded that morphine withdrawal can strongly enhance phosphorylation of HSP27 at Ser15, irrespective whether it is spontaneous (in the case of morphine abstinence) or elicited by treatment with the opioid inverse agonist naloxone.

In order to determine whether HSP27 could be activated after morphine treatment or withdrawal, its phosphorylation was assessed by immunoblotting with specific antibodies against phosphorylated amino acids Ser82 and Ser15 of HSP27 (Fig. 2). Both sites can be phosphorylated by MAPKAP-2 and the major site of phosphorylation of this protein is Ser82 [88]. It was verified using antibodies against HSP27 that morphine treatment led to up-regulation of HSP27. Nevertheless, it resulted also in phosphorylation of Ser15 and in less extent in phosphorylation of Ser82 (Fig. 2).

Non-phosporylated HSP27 was shown to exist in the form of large oligomers and its phosphorylation leads to dissociation of these oligomers [91]. Phosphorylation and oligomerization of HSP27 is connected with modulation of the interaction between HSP27 and actin [91]–[92]. While the non-phosphorylated HSP27 can block actin polymerization, the phosphorylation of HSP27 is related to re-organization of actin-based cytoskeletal structures [88]. It has been suggested that this re-organization of the actin cytoskeleton induced by phosphorylation of HSP27 could lead to cytoprotection due to stabilization of actin filaments [93]. The phosphorylation and activation of HSP27 might be also related to the observed down-regulation of tau after 3 days of morphine abstinence. HSP27 and α-B crystallin can interact with microtubules and neurofilaments and protect against protein aggregation [94]. Both proteins can interact with pathological hyperphosphorylated tau and thus facilitate its dephosphorylation and degradation. This process was observed mainly in studies dealing with Alzheimer disease [94]–[96].

### Conclusions and Future Directions

This work follows up our previous research focused on the consequences of prolonged morphine administration on the rat heart [41] and further elaborates this issue. Results of our present proteomic study clearly indicate that iTRAQ approach may yield a wealth of information regarding the effect of morphine on the cardiac proteome. Quantitative analysis of proteomics data obtained by this method revealed a number of significant changes induced by both morphine exposure and withdrawal. An important outcome from this study is the realization that the protein and hence gene expression responses to morphine in the heart are quite complex. A similarly wide-ranging response to morphine has been previously observed in brain tissue.

A number of significant changes found in the expression of different myocardial proteins due to morphine treatment suggest that the potential cardiac effects of this drug should be carefully taken into account when using it for medical purposes. Importantly, as can be seen from this study, morphine withdrawal may apparently have even a greater impact on the heart proteome than the use of this compound itself. Hence, morphine should be considered as a drug with potentially profound effects on cardiac protein expression profiling, which may have been difficult to appreciate so far. Future studies should elucidate whether and to what extent morphine-induced changes in protein expression may play a specific role in the regulation of heart function.

## Supporting Information

Table S1 **Complete list of the myocardial proteins altered after morphine treatment or withdrawal.** The proteins whose expression levels were altered at least twice after morphine treatment (M) or drug withdrawal for 3 days (M_W_-I) or 6 days (M_W_-II) compared to controls were arranged according to their function into several groups. Expression values of up-regulated (↑) or down-regulated (↓) proteins are expressed as fold change from untreated controls. Number of accession (gi numbers from GenBank/EMBL/DDBJ databases) and fraction in which protein alteration was detected are quoted for each protein (CS, cytosol; PM, plasma membrane-enriched fraction; MT, mitochondria-enriched fraction). %Cov, the percentage of matching amino acids from the identified peptides divided by the total number of amino acids in the sequence. Peptides, number of unique peptides per identified protein. The occurrence of individual proteins in other fraction(s) without alterations after morphine treatment or withdrawal is mentioned in Notes noted using the following markings: (1) CS, no change; (2) PM, no change; (3) MT, no change; (4) CS+PM, no change; (5) PM+MT, no change.
(PDF)

Table S2 **Complete list of the myocardial proteins whose levels were not significantly altered after morphine treatment or withdrawal.** The proteins whose expression levels were not significantly altered after morphine treatment (M) or withdrawal for 3 days (M_W_-I) or 6 days (M_W_-II) compared to controls were arranged according to their function into several groups. Number of accession (gi numbers from GenBank/EMBL/DDBJ databases) and fraction in which the protein was detected are quoted for each protein (CS, cytosol; PM, plasma membrane-enriched fraction; MT, mitochondria-enriched fraction). %Cov, the percentage of matching amino acids from identified peptides divided by the total number of amino acids in the sequence. Peptides, number of unique peptides per identified protein.
(PDF)
