# Supplementary material for: Global Changes in the Rat Heart Proteome Induced by Prolonged Morphine Treatment and Withdrawal
Source: PLoS One. 2012 Oct 9;7(10):e47167. doi: 10.1371/journal.pone.0047167 (PMC3467212; doi:10.1371/journal.pone.0047167)
Supplement: Table S1 — Complete list of the myocardial proteins altered after morphine treatment or withdrawal. The proteins whose expression levels were altered at least twice after morphine treatment (M) or drug withdrawal for 3 days (MW-I) or 6 days (MW-II) compared to controls were arranged according to their function into several groups. Expression values of up-regulated (↑) or down-regulated (↓) proteins are expressed as fold change from untreated controls. Number of accession (gi numbers from GenBank/EMBL/DDBJ databases) and fraction in which protein alteration was detected are quoted for each protein (CS, cytosol; PM, plasma membrane-enriched fraction; MT, mitochondria-enriched fraction). %Cov, the percentage of matching amino acids from the identified peptides divided by the total number of amino acids in the sequence. Peptides, number of unique peptides per identified protein. The occurrence of individual proteins in other fraction(s) without alterations after morphine treatment or withdrawal is mentioned in Notes noted using the following markings: (1) CS, no change; (2) PM, no change; (3) MT, no change; (4) CS+PM, no change; (5) PM+MT, no change. (PDF) [file pone.0047167.s001.pdf]

Table S1. Complete list of the myocardial proteins altered after morphine treatment or withdrawal.

# Accession	Protein	Fraction	%Cov	Peptides	M	M_W-I	M_W-II	Notes
Metabolism								
gi|37748456	2,4-dienoyl CoA reductase 1	CS	21.2	3			4.3 (↑)	(3)
gi|55250706	3'(2'), 5'-bisphosphate nucleotidase 1	CS	14.3	2		2.8 (↑)		
gi|149027156	Acetyl-CoA acyltransferase 2	MT	78.6	36			3.2 (↓)	(4)
gi|60391194	Aconitate hydratase, mitochondrial	MT	52.7	39		2.2 (↓)		(4)
gi|157817043	Acyl-CoA thioesterase 13	CS	45.0	5		3.8 (↓)		(2)
gi|48734846	Acyl-CoA dehydrogenase, C-2 to C-3 short chain	MT	43.7	3		2.1 (↓)	2.1 (↓)	(4)
gi|56541110	Acyl-CoA dehydrogenase, very long chain	CS	39.4	13			2.7 (↑)	(5)
gi|61889092	Adenylate kinase 1	CS	72.7	13			2.1 (↑)	
gi|77020256	Adenylate kinase 2 isoform b	CS	32.8	7		2.8 (↑)		(5)
gi|976252	AIR carboxylase-SAICAR synthetase	CS	8.9	2		2.0 (↑)		
gi|974168	Aldehyde dehydrogenase	CS	13.2	3		12.3 (↓)		
gi|6978491	Aldose reductase family 1, member B1	CS	62.3	21	2.0 (↓)			
gi|38197390	Aspartate aminotransferase 1	CS	80.4	53		2.1(↓)		(5)
gi|197246855	Biliverdin reductase B	CS	66.5	10		3.3 (↓)		
gi|81894530	Carboxymethylenebutenolidase homolog	CS	41.2	9	2.0 (↓)			
gi|33086660	Cc1-8	MT	20.4	10		3.2 (↓)		
gi|55741551	Coiled-coil-helix-coiled-coil-helix domain containing 10	CS	36.2	2		2.0 (↑)		
gi|54145374	Cytochrome c oxidase subunit 1	MT	8.8	3			2.2 (↑)	(2)
gi|6015047	Delta(3,5)-Delta(2,4)-dienoyl-CoA isomerase	PM	26.9	8	2.1 (↓)			(1)
		MT	19.9	4			2.7 (↓)	
gi|78365255	Dihydrolipoamide S-acetyltransferase	PM	50.3	22			2.6 (↓)	
gi|157786744	Dihydropyrimidinase-like 2	CS	22.6	6	3.6 (↓)	2.1 (↓)		
gi|81911114	Dimethylarginine dimethylaminohydrolase 2	CS	18.3	4	3.6 (↑)	7.9 (↑)	2.7 (↑)	
gi|55716049	Epoxide hydrolase 2, cytoplasmic	CS	29.6	5			2.3 (↑)	
gi|52782765	Flavoprotein subunit of complex II	MT	73.3	60	2.1 (↑)		4.6 (↑)	(4)
gi|6978487	Fructose-bisphosphate aldolase A	MT	58.0	15		2.0 (↓)		(4)
gi|227665	Fumarase	MT	25.3	6		2.2 (↓)		
gi|204188	Fumarase precursor	CS	63.5	35	2.1 (↑)		2.0 (↑)	
gi|281332160	Glucan (1,4-α-), branching enzyme 1	CS	14.8	7			2.2 (↓)	
gi|92090591	Glutamate dehydrogenase 1, mitochondrial	PM	39.4	17			2.4 (↓)	(1)
		MT	23.7	10		2.5 (↓)	2.1 (↓)	
gi|157823513	Glutaredoxin 5	CS	35.5	4		2.3 (↑)		

# Accession	Protein	Fraction	%Cov	Peptides	M	M_W-I	M_W-II	Notes
gi|9798638	Glyceraldehyde-3-phosphate dehydrogenase	CS	93.7	81	3.4 (↓)			(5)
gi|66911967	Hagh protein	CS	11.2	2		2.5 (↑)		
gi|7549765	Hexokinase 2	CS	6.0	2		2.1 (↑)		
gi|31077132	Histidine rich calcium binding protein	PM	39.1	20		3.0 (↑)	3.9 (↑)	(3)
gi|7387725	Hydroxyacyl-CoA dehydrogenase	CS	73.9	29			2.9 (↑)	(5)
gi|68534712	Hypoxanthine phosphoribosyl-transferase 1	CS	51.8	11		2.5 (↓)		(2)
gi|68051964	Isocitrate dehydrogenase [NAD] β	CS	36.9	10	3.0 (↓)		2.2 (↓)	(5)
gi|62079055	Isocitrate dehydrogenase 2 ($NADP^+$), mitochondrial precursor	MT	62.8	27		3.0 (↓)	2.2 (↓)	(4)
gi|8393706	Lactate dehydrogenase A	CS	76.5	37			3.4 (↓)	(5)
gi|6981146	Lactate dehydrogenase B	MT	37.7	13		2.0 (↓)		(4)
gi|6978431	Long-chain acyl-CoA dehydrogenase precursor	MT	61.9	21		2.0 (↓)		(4)
gi|50400214	L-xylulose reductase	CS	20.5	3	2.5 (↓)		2.0 (↓)	(5)
gi|81882966	Methylcrotonoyl-CoA carboxylase subunit α, mitochondrial	CS	14.8	3			5.5 (↓)	(5)
gi|220655	Mitochondrial acetoacetyl-CoA thiolase	MT	73.4	21	3.6 (↓)		3.3 (↓)	
gi|45737868	Mitochondrial aldehyde dehydrogenase precursor	MT	18.6	6			2.0 (↓)	
gi|157820787	NADH dehydrogenase 1, α/β subcomplex, 1	CS	24.4	4		3.3 (↓)		(2)
		MT	59.0	11			11.6 (↓)	
gi|81882328	NADH dehydrogenase 1, α subcomplex subunit 10	MT	81.7	42			2.1 (↑)	(2)
gi|81882598	NADH dehydrogenase 1, α subcomplex subunit 9	MT	70.3	31			2.0 (↑)	(2)
gi|81885576	NADH dehydrogenase flavoprotein 3	MT	71.3	14			2.5 (↓)	
gi|108773605	NADH dehydrogenase subunit 5	MT	16.6	8			2.8 (↑)	
gi|54145382	NADH dehydrogenase subunit 5	PM	13.0	7		2.1 (↓)	2.0 (↓)	
gi|76677911	Obg-like ATPase 1	CS	22.7	3		18.0 (↓)		
gi|119364627	PDHE1-A type I, precursor	CS	22.1	3		3.6 (↑)		
gi|8393948	Phosphoglycerate mutase 2	MT	36.4	10		2.8 (↓)		(4)
gi|281332119	Phosphoglycolate phosphatase	CS	12.2	2		2.2 (↑)		
gi|171846774	Phosphorylase, glycogen, muscle	MT	24.9	10		2.0 (↓)		(2)
gi|56072	Precursor polypeptide (AA -29 to 261)	MT	39.3	6		2.2 (↓)		(4)
gi|62657641	PREDICTED: similar to Trifunctional purine biosynthetic protein adenosine-3	CS	9.3	2	9.8 (↓)			
gi|51260066	Propionyl-CoA carboxylase, β polypeptide	MT	20.7	19		2.0 (↓)		(4)
gi|6981420	Protease, serine, 2 precursor	CS	12.6	16		5.9 (↓)	3.0 (↓)	(5)
gi|149023098	Protein disulfide isomerase associated 3	CS	32.9	10		2.1 (↓)		

# Accession	Protein	Fraction	%Cov	Peptides	M	M_W-I	M_W-II	Notes
gi|48675845	Purine biosynthesis protein PURH	CS	25.2	8	3.0 (↓)			
gi|71051030	Pyruvate dehydrogenase α 1	PM	75.1	24			2.8 (↓)	(3)
gi|56090293	Pyruvate dehydrogenase β precursor	CS	28.7	5	2.1 (↑)		2.4 (↑)	(2)
		MT	49.6	13		2.5 (↓)		
gi|59709473	Pyruvate dehydrogenase kinase 1 precursor	CS	27.0	5	2.6 (↓)			(2)
gi|694003	Pyruvate dehydrogenase kinase 2 subunit p45	CS	23.6	4	2.0 (↓)			(2)
gi|149029697	rCG42519, isoform CRA_a	MT	36.1	11			2.0 (↓)	(4)
gi|189083744	Sarcomeric mitochondrial creatine kinase precursor	CS	58.2	18		2.0 (↑)	2.3 (↑)	(5)
gi|223634703	Suclg1	CS	28.6	10	2.0 (↓)			(5)
gi|205829936	Succinyl-CoA:3-ketoacid-CoA transferase 1,	CS	52.3	29	2.2 (↓)			
	mitochondrial	PM	44.6	14	2.3 (↓)			
		MT	41.9	13		2.5 (↓)		
gi|485267	Transketolase	CS	14.7	5	2.1 (↓)			
gi|8394544	Xanthine dehydrogenase	CS	5.4	2		6.5 (↓)		
Cell cycle								
gi|97537204	Aortic preferentially expressed protein 1	MT	9.3	5		3.4 (↑)		
gi|54035288	Enolase 3, beta, muscle	CS	59.0	29		2.0 (↓)		(5)
gi|407164	Heat shock protein 70	CS	44.2	20	15.9 (↑)	6.3 (↑)	6.8 (↑)	
gi|48734832	NSFL1 (p97) cofactor (p47)	PM	26.8	4			3.8 (↓)	(1)
gi|157819939	Programmed cell death 5	CS	32.0	1	2.1 (↑)			
Regulation of protein expression								
gi|81883726	Alanyl-tRNA synthetase domain-containing protein 1	CS	18.2	3		2.2 (↑)		
gi|8650478	Cellular nucleic acid binding protein	CS	13.6	2	10.6 (↓)		2.4 (↓)	
gi|220698	Contrapsin-like protease inhibitor (CPi-21)	CS	47.8	19		2.0 (↓)		
gi|81892734	Cytosolic non-specific dipeptidase	CS	8.0	1		2.0 (↑)		
gi|190359305	Elongation factor Tu, mitochondrial	CS	46.2	17		2.5 (↑)		(5)
gi|61556967	Eukaryotic translation elongation factor 1 δ	CS	11.4	2		2.2 (↑)		
gi|8393296	Eukaryotic translation elongation factor 2	CS	42.0	22			2.8 (↑)	(5)
gi|81907626	Four and a half LIM domains protein 1	CS	23.9	5		2.0 (↑)		
gi|6226139	Four and a half LIM domains protein 2	MT	18.6	3	2.3 (↓)	2.5 (↑)	5.4 (↓)	
gi|55250714	G elongation factor, mitochondrial 1	CS	20.2	8	2.6 (↓)			

# Accession	Protein	Fraction	%Cov	Peptides	M	M_W-I	M_W-II	Notes
gi|6981052	Heat shock 10 kDa protein 1	CS	42.2	5	2.1 (↑)		2.3 (↑)	(2)
		MT	39.2	2			2.1 (↓)	
gi|56383	Heat shock protein (hsp60) precursor	CS	63.5	35	2.0 (↑)		2.0 (↑)	(3)
gi|224493240	hnRNP A2 / hnRNP B1	CS	16.4	3			2.0 (↓)	
gi|951425	Housekeeping protein	CS	76.2	13	2.3 (↑)			(2)
gi|6981076	Insulin-degrading enzyme	CS	6.2	1		2.5 (↑)		
gi|81916424	Lon protease homolog, mitochondrial	CS	12.2	4		2.6 (↓)		(5)
gi|6978513	N-acylaminoacyl-peptide hydrolase	CS	10.3	2		2.5 (↑)		
gi|52789215	Poly(A) binding protein, cytoplasmic 1	CS	8.0	2			2.2 (↓)	
gi|157786694	Polymerase I and transcript repase factor	MT	16.6	3			2.4 (↑)	
gi|55855	Precursor (AA -17 to 399)	CS	31.3	7		2.8 (↓)		(5)
gi|72255509	Proteasome 26S subunit, non-ATPase, 2	CS	4.3	1	9.6 (↓)	10.9 (↑)		
gi|38014563	Proteasome subunit, α type 5	CS	19.9	2	2.4 (↑)			
gi|52345385	Protein disulfide isomerase A6	CS	9.9	2		5.1 (↑)		
gi|56970	Prothrombin precursor	CS	19.3	2	5.7 (↑)	6.5 (↑)		
gi|149054664	rCG35339	PM	11.0	3	2.1 (↑)			
gi|149028555	rCG36203, isoform CRA_a	CS	21.5	1		11.2 (↓)	2.6 (↓)	
gi|40018548	Serpinb6a	CS	9.5	2		4.1 (↓)		
gi|157819737	Seryl-tRNA synthetase 2	CS	14.1	2		2.5 (↑)		
gi|209571551	Tubulin tyrosine ligase-like family, member 12	CS	6.1	2		17.0 (↓)		
gi|210032365	Tumor rejection antigen gp96 precursor	CS	17.3	5	4.1 (↓)			(5)
gi|90111992	Ubiquinone biosynthesis protein COQ9, mitochondrial	PM	23.4	6	4.2 (↓)			(5)
gi|92373398	Y box binding protein 1	CS	48.5	5			2.0 (↓)	
Signal transduction								
gi|61216932	14-3-3 protein ε	CS	63.1	16	2.0 (↓)			(1)
gi|5732982	Carboxylesterase	CS	9.4	2		4.0 (↑)	2.7 (↓)	
gi|56405004	COP9 signalosome complex subunit 4	CS	10.8	1		3.1 (↓)	2.1 (↑)	
gi|157822045	Hedgehog acyltransferase-like	MT	20.7	7		2.1 (↑)	2.3 (↑)	(2)
gi|120474989	Keratin 1	CS	23.2	10	11.6 (↓)			
		PM	39.4	18	2.2 (↓)		2.3 (↓)	(3)
gi|54035563	Ldb3 protein	CS	24.4	3		5.4 (↓)		
		MT	31.8	8		2.1 (↑)	6.2 (↓)	

# Accession	Protein	Fraction	%Cov	Peptides	M	M_W-I	M_W-II	Notes
gi|71051822	LOC683313 protein	CS	20.7	4	25.4 (↓)			(3)
		PM	22.5	10			2.9 (↓)	
gi|62461582	Mitogen-activated protein kinase 14	CS	9.4	1	2.5 (↑)			
gi|8393981	Phospholipase C, delta 1	CS	9.8	2	3.2 (↑)	10.7 (↓)		
Structure								
gi|77993370	Actin alpha cardiac 1	PM	61.3	23			2.5 (↑)	(1)
		MT	78.5	94		3.0 (↑)	6.1 (↓)	
gi|281332157	Actinin alpha 2	MT	65.3	58		2.0 (↑)	3.0 (↓)	
gi|207158	Big tau	CS	7.1	2		17.0 (↓)		
gi|205495	Cardiac myosin light chain 2	MT	90.4	40			5.9 (↓)	(1)
gi|729088	CD9 antigen	PM	21.7	2	2.1 (↑)	2.1 (↑)	2.4 (↑)	
gi|281427229	Collagen, type VI, alpha 2	PM	16.3	6		2.0 (↑)	2.7 (↑)	(3)
gi|38197676	Desmin	MT	80.2	32			3.5 (↓)	(1)
gi|452779	Desmin	PM	24.7	4		2.0 (↑)	2.8 (↑)	
gi|75991707	Destrin	CS	61.2	9			2.1 (↓)	
gi|83302140	Dihydropyrimidinase-related protein 3	CS	8.2	2	3.0 (↓)	7.9 (↓)		
gi|77917614	Dynamin 1-like	CS	17.9	6		3.5 (↑)		
gi|149056792	Echinoderm microtubule associated protein like 2	CS	11.1	3	2.9 (↑)			
gi|81883744	Evolutionarily conserved signaling intermediate in Toll pathway	MT	8.5	2		2.7 (↑)		(2)
gi|56090614	Filamin binding LIM protein 1	CS	16.5	3	3.2 (↑)		4.3 (↑)	
gi|46237655	Flotillin 1	PM	8.8	1			2.1 (↑)	
gi|56847618	Keratin 16	PM	16.6	5			2.1 (↓)	
gi|56912231	Keratin 33A	PM	15.8	5	5.9 (↑)	14.3 (↑)	4.9 (↑)	
gi|67678416	Keratin 8	PM	21.3	10	3.5 (↓)	2.4 (↓)	3.1 (↓)	
gi|81891690	Keratin, type I cytoskeletal 10	CS	49.4	10	23.3 (↓)		2.5 (↓)	
		PM	32.7	20	4.3 (↓)	3.2 (↓)	2.4 (↓)	
		MT	30.0	12			2.5 (↓)	
gi|81891674	Keratin, type I cytoskeletal 17	PM	27.0	4			2.1 (↓)	
gi|81891673	Keratin, type I cytoskeletal 42	CS	17.7	3	18.9 (↓)			
		PM	24.6	6			2.2 (↓)	
		MT	18.6	4		2.1 (↑)		
gi|81891699	Keratin, type II cytoskeletal 2 epidermal	CS	31.1	7	3.3 (↓)		2.2 (↓)	(3)
		PM	42.9	12	3.1 (↓)	2.9 (↓)	2.4 (↓)	

# Accession	Protein	Fraction	%Cov	Peptides	M	M_W-I	M_W-II	Notes
gi|81170669	Keratin, type II cytoskeletal 5	CS	19.4	11	20.1 (↓)			
		PM	25.4	16			2.6 (↓)	
		MT	20.3	11			2.7 (↓)	
gi|281371490	Laminin, gamma 1	PM	11.2	4		2.3 (↑)	2.5 (↑)	(3)
gi|6625487	Lanthionine synthetase C-like protein 1	CS	6.8	1		2.5 (↑)		
gi|207352	Minor striated-muscle α tropomyosin	PM	51.4	7			2.5 (↑)	
gi|149042266	Moesin, isoform CRA_a	CS	25.8	7		2.0 (↑)	2.0 (↓)	(2)
gi|281306803	Myomesin 2	MT	24.5	12		2.6 (↑)	3.1 (↓)	(1)
gi|149025875	Myozenin 2, isoform CRA_a	MT	44.3	8		2.2 (↑)	3.2 (↓)	
gi|282154799	NFU1 iron-sulfur cluster scaffold homolog precursor	CS	15.8	2		2.3 (↑)		
gi|215276950	Plakophilin 2	MT	15.9	2		2.3 (↑)	7.8 (↓)	
gi|40849886	Plectin 1	MT	15.8	11			2.5 (↓)	
gi|9789715	Septin-7	CS	9.9	1	23.5 (↓)	2.1 (↑)	2.2 (↓)	
gi|2555185	SH3-containing protein p4015	MT	6.1	2		2.6 (↑)		
gi|78103170	Small muscular protein	CS	38.8	2	8.5 (↑)	7.2 (↑)	4.7 (↑)	
gi|171846571	Tnnt2 protein	MT	52.5	22		2.0 (↑)	6.8 (↓)	
gi|149040761	Transgelin 2, isoform CRA_b	CS	32.8	2		7.0 (↓)		
gi|48675841	Tropomodulin 1	MT	19.5	3		2.4 (↑)	2.0 (↓)	
gi|92090646	Tropomyosin alpha-1 chain	MT	81.7	38			5.3 (↓)	(1)
gi|8394469	Troponin 1, type 3 (cardiac muscle)	MT	66.4	14			7.3 (↓)	
gi|77627992	Troponin C type 1 (slow)	MT	46.0	7			10.7 (↓)	
gi|6981666	Troponin T type 2 (cardiac)	CS	30.1	6		2.8 (↓)		(2)
gi|81883217	Tubulin polymerization-promoting protein family member 3	CS	24.4	1			3.6 (↓)	
gi|57480	Vimentin	PM	26.4	7	2.1 (↑)	3.0 (↑)	2.2 (↑)	(3)
Transport								
gi|55391508	Albumin	CS	82.2	188	5.8 (↓)		2.0 (↑)	
gi|763181	Annexin VI	PM	26.3	12	2.1 (↓)			(3)
gi|55824759	Apolipoprotein E	CS	40.1	4	2.3 (↑)		2.8 (↑)	(3)
gi|71681130	ATP synthase, beta polypeptide	CS	60.9	22			3.6 (↑)	(2)
		MT	83.7	206			2.1 (↑)	
gi|56252	Beta-globin	MT	78.2	13			2.6 (↓)	(2)
gi|93279422	Chain A, Rat Liver F1-Atpase	CS	53.7	27			2.9 (↑)	(2)
gi|81884378	Cytochrome b-c1 complex subunit 1	MT	66.3	81			2.0 (↑)	(2)

# Accession	Protein	Fraction	%Cov	Peptides	M	M_W-I	M_W-II	Notes
gi|62511137	Cytochrome b-c1 complex subunit 6	PM	77.5	24		2.2 (↑)		(3)
gi|81910618	EH domain-containing protein 1	CS	14.2	4	2.4 (↓)	2.3 (↑)		(3)
gi|204080	Fatty acid binding protein	MT	91.7	19		3.9 (↓)		(1)
gi|149068324	Importin 7	CS	8.0	2		10.4 (↑)		
gi|81864913	Mitochondrial import receptor subunit TOM22 homolog	MT	56.3	6			2.1 (↑)	(2)
gi|6155712	Nicotinamide nucleotide transhydrogenase	MT	48.2	79	2.1 (↑)	2.4 (↑)	2.8 (↑)	
gi|38014819	Solute carrier family 25 , member 4	MT	84.2	114			2.2 (↑)	(2)
gi|189491614	Solute carrier family 25, member 46	MT	11.5	2	2.0 (↓)	12.1 (↓)		
gi|76443687	Solute carrier family 4, member 1	PM	25.5	14			2.3 (↓)	
gi|6981684	Transthyretin precursor	CS	46.3	3	11.2 (↓)	10.5 (↓)	8.3 (↓)	
gi|55628	Unnamed protein product	MT	66.5	34		2.4 (↓)		
gi|4558734	Voltage dependent anion channel	MT	74.2	31			2.0 (↑)	
gi|8810247	Voltage-dependent anion channel 2	MT	72.2	29			2.3 (↑)	(2)
gi|802111	Zero beta-globin	CS	76.7	32			3.7 (↓)	
Immunity								
gi|158138561	Complement component 3	MT	8.4	5		2.7 (↓)		(4)
gi|992567	MHC class I RT1.Aw3 protein	PM	28.9	7			2.4 (↑)	
gi|81170680	Mitochondrial antiviral-signaling protein	PM	16.6	1	2.4 (↑)	2.5 (↑)		(3)
gi|81892278	Plasma protease C1 inhibitor	CS	11.7	1	6.3 (↓)	2.3 (↓)		
Metabolism + regulation of protein expression								
gi|744592	Alpha-B crystallin	CS	78.3	24	2.1 (↑)		2.1 (↑)	(3)
gi|81861326	Prolyl endopeptidase	CS	7.5	3		3.5 (↓)	2.3 (↓)	
Metabolism + transport								
gi|6978515	Apolipoprotein A-I precursor	CS	66.0	11	2.1 (↑)			(2)
gi|6980972	Aspartate aminotransferase 2	MT	54.9	21		2.9 (↓)	2.9 (↓)	(4)
gi|6978705	Carnitine O-palmitoyltransferase precursor	CS	34.5	10			2.6 (↑)	(5)
gi|83305118	NADH dehydrogenase flavoprotein 2	CS	39.1	5	5.2 (↑)			(5)
gi|81884209	NADH-ubiquinone oxidoreductase 75 kDa subunit	CS	8.8	2		2.0 (↑)		(5)
Signal transduction+ structure								
gi|575380	Caveolin	PM	50.5	10			2.0 (↑)	(3)
gi|56797757	Fibrinogen α chain isoform 1	MT	20.0	7			2.6 (↓)	
gi|149063018	Heat shock 27kDa protein 1	CS	74.8	14	2.6 (↑)			

# Accession	Protein	Fraction	%Cov	Peptides	M	M_W-I	M_W-II	Notes
gi|207028435	Hypothetical protein LOC684352	CS	12.9	2		3.4 (↓)		
gi|83816931	Junctophilin 2	PM	25.4	12			2.6 (↑)	(3)
gi|266495	Myristoylated alanine-rich C-kinase substrate	CS	28.8	2	3.4 (↓)			
		PM	24.0	3		2.2 (↓)	2.0 (↓)	(3)
Cell cycle and apoptosis + signal transduction								
gi|58865636	ADP-ribosyltransferase 3	PM	27.5	4	3.1 (↑)	2.4 (↑)	3.6 (↑)	(3)
Cell cycle and apoptosis + signal transduction + metabolism								
gi|8394502	Ubiquitin C	CS	91.4	7			2.1 (↓)	(5)
Cell cycle and apoptosis + regulation of protein expression								
gi|58865450	Bcl2-associated athanogene 3	CS	23.9	3	2.4 (↑)			
gi|6978715	Cystatin B	CS	48.0	2	2.2 (↓)			
gi|55977739	Heat shock 70 kDa protein 1A/1B	MT	22.6	9	2.3 (↑)			(2)
gi|62201921	Prothymosin α	CS	27.7	3		4.9 (↓)		
Immunity + cell cycle								
gi|806379	RB13-6 antigen	PM	5.4	2	2.0 (↑)		2.0 (↑)	
Immunity + regulation of protein expression								
gi|8394060	Proteasome α 1 subunit	CS	15.2	1		2.3 (↑)		
Transport + signal transduction								
gi|6978896	Gap junction protein, alpha 1	MT	35.3	10			2.5 (↑)	(2)
gi|40254781	GDP dissociation inhibitor 2	CS	36.4	8		2.3 (↓)		
Transport + regulation of protein expression								
gi|157820325	Chromosome segregation 1-like	CS	6.2	2		2.4 (↑)		
gi|60097941	Haptoglobin precursor	CS	32.0	10	2.2 (↓)			
Structure + transport								
gi|157824043	Myosin binding protein C, cardiac	MT	63.7	52		2.0 (↑)	4.2 (↓)	(1)
gi|186659510	Myosin, heavy polypeptide 6 α	MT	77.9	441			6.5 (↓)	
gi|763179	Unnamed protein product	PM	47.0	7			2.5 (↑)	(1)
	(similar to Myosin light chain 3)	MT	89.5	42		2.0 (↑)	12.4 (↓)	

# Accession	Protein	Fraction	%Cov	Peptides	M	M_W-I	M_W-II	Notes
gi|56655	Unnamed protein product	PM	18.8	22			2.9 (↑)	
Cell cycle and apoptosis + regulation of protein expression + transport								
gi|210032180	Programmed cell death 6 interacting protein	CS	13.1	2			2.6 (↑)	
Unknown function								
gi|42417059	Cardiac titin fetal N2BA isoform middle Ig	MT	9.8	20		2.0 (↑)	2.1 (↓)	
gi|77627996	Hypothetical protein LOC298384	CS	31.9	2		2.0 (↑)		
gi|157819345	Hypothetical protein LOC313776	PM	9.8	4	2.4 (↑)	3.7 (↑)		(3)
gi|2780408	MIPP65	MT	37.1	15		4.6 (↓)		(2)
gi|34873230	PREDICTED: similar to abhydrolase domain containing 11	CS	11.4	1		2.9 (↑)		
gi|62644491	PREDICTED: similar to Interferon- induced guanylate-binding protein 1	CS	16.5	5		3.9 (↑)	3.6 (↑)	
gi|109487640	PREDICTED: similar to Myomesin-1	MT	17.1	12		2.5 (↑)	5.7 (↓)	
gi|109506129	PREDICTED: similar to Nebulette	MT	25.9	12		2.1 (↑)	2.5 (↓)	
gi|109490823	PREDICTED: similar to obscurin	MT	8.4	5		2.2 (↑)	3.0 (↓)	
gi|34854800	PREDICTED: similar to solute carrier family 25	MT	42.9	9			2.9 (↑)	(2)
gi|109463865	PREDICTED: similar to sorbin and SH3 domain containing 1 isoform 3	MT	9.6	3		2.0 (↑)	2.1 (↓)	
gi|109486250	PREDICTED: similar to tensin	MT	6.9	2		2.2 (↑)		
gi|149037631	Procollagen, type VI, alpha 3	PM	12.2	13		2.0 (↑)	3.0 (↑)	(3)
gi|149063941	rCG23467, isoform CRA_a	MT	75.6	358			3.5 (↓)	
gi|149026101	rCG28661, isoform CRA_b	MT	19.3	4	2.5 (↓)	2.1 (↑)	12.0 (↓)	
gi|149031601	rCG45246	MT	40.6	4		2.1 (↑)		
gi|149035504	rCG50422, isoform CRA_d	CS	23.4	2	2.1 (↑)	2.4 (↑)	3.8 (↑)	
gi|149056475	rCG54610, isoform CRA_a	MT	27.8	5			2.4 (↑)	(2)
gi|55628	Unnamed protein product	CS	85.7	192		5.4 (↓)		
gi|57506	Zinc binding protein	CS	33.0	1		5.0 (↓)		

The proteins whose expression levels were altered at least twice after morphine treatment (M) or drug withdrawal (M_W-I and M_W-II) compared to controls were arranged according to their function into several groups. Number of accession (gi numbers from GenBank/EMBL/DDBJ databases) and fraction in which protein alteration was detected are quoted for each protein (CS, cytosol; PM, plasma membrane-enriched fraction; MT, mitochondria-enriched fraction). %Cov, the percentage of matching amino acids from the identified peptides divided by the total number of amino acids in the sequence. Peptides, number of unique peptides per identified protein. The occurrence of individual proteins in

other fraction(s) without alterations after morphine treatment or withdrawal is mentioned in Notes noted using the following markings: (1) CS, no change; (2) PM, no change; (3) MT, no change; (4) CS+PM, no change; (5) PM+MT, no change.
