# Supplementary material for: Global Changes in the Rat Heart Proteome Induced by Prolonged Morphine Treatment and Withdrawal
Source: PLoS One. 2012 Oct 9;7(10):e47167. doi: 10.1371/journal.pone.0047167 (PMC3467212; doi:10.1371/journal.pone.0047167)
Supplement: Table S2 — Complete list of the myocardial proteins whose levels were not significantly altered after morphine treatment or withdrawal. The proteins whose expression levels were not significantly altered after morphine treatment (M) or withdrawal for 3 days (MW-I) or 6 days (MW-II) compared to controls were arranged according to their function into several groups. Number of accession (gi numbers from GenBank/EMBL/DDBJ databases) and fraction in which the protein was detected are quoted for each protein (CS, cytosol; PM, plasma membrane-enriched fraction; MT, mitochondria-enriched fraction). %Cov, the percentage of matching amino acids from identified peptides divided by the total number of amino acids in the sequence. Peptides, number of unique peptides per identified protein. (PDF) [file pone.0047167.s002.pdf]

**Table S2. Complete list of the myocardial proteins whose levels were not significantly altered after morphine treatment or withdrawal.**

| # Accession | Protein name | % Cov | Peptides | Fraction |
|---|---|---|---|---|
| gi\|81883737 | [Protein ADP-ribosylarginine] hydrolase-like protein 1 | 26,6 | 6 | CS |
| gi\|61216932 | 14-3-3 protein epsilon | 23,1 | 2 | MT |
| gi\|1051270 | 14-3-3 zeta isoform | 57,1 | 11 | MT |
| gi\|37748456 | 2,4-dienoyl CoA reductase 1, mitochondrial | 60,6 | 24 | MT |
| gi\|67476443 | 2,4-dienoyl-CoA reductase, mitochondrial | 57,9 | 25 | PM |
| gi\|81883712 | 2-oxoglutarate dehydrogenase E1 component, mitochondrial | 40 | 27 | CS |
| gi\|81883712 | 2-oxoglutarate dehydrogenase E1 component, mitochondrial | 61,2 | 52 | PM |
| gi\|81883712 | 2-oxoglutarate dehydrogenase E1 component, mitochondrial | 45,2 | 35 | MT |
| gi\|57333 | 3-2trans-enoyl-CoA isomerase | 53,6 | 14 | CS |
| gi\|57333 | 3-2trans-enoyl-CoA isomerase | 36 | 9 | PM |
| gi\|57333 | 3-2trans-enoyl-CoA isomerase | 26 | 4 | MT |
| gi\|7387724 | 3-hydroxyacyl-CoA dehydrogenase type-2 | 82,4 | 22 | CS |
| gi\|7387724 | 3-hydroxyacyl-CoA dehydrogenase type-2 | 41,8 | 5 | PM |
| gi\|7387724 | 3-hydroxyacyl-CoA dehydrogenase type-2 | 38,7 | 6 | MT |
| gi\|83977457 | 3-hydroxyisobutyrate dehydrogenase precursor | 27,2 | 6 | CS |
| gi\|83977457 | 3-hydroxyisobutyrate dehydrogenase precursor | 17,9 | 3 | PM |
| gi\|149046212 | 3-hydroxyisobutyryl-Coenzyme A hydrolase | 41 | 10 | CS |
| gi\|61556993 | 3-hydroxyisobutyryl-Coenzyme A hydrolase | 13,2 | 3 | PM |
| gi\|38511566 | 3-hydroxymethyl-3-methylglutaryl-Coenzyme A lyase | 38,2 | 3 | CS |
| gi\|54039450 | 40S ribosomal protein S15a | 41,5 | 2 | PM |
| gi\|54039450 | 40S ribosomal protein S15a | 36,2 | 2 | MT |
| gi\|50403620 | 40S ribosomal protein S18 | 22,4 | 2 | PM |
| gi\|50403620 | 40S ribosomal protein S18 | 34,2 | 3 | MT |
| gi\|50403678 | 40S ribosomal protein S23 | 18,9 | 2 | PM |
| gi\|51338623 | 40S ribosomal protein S28 | 33,3 | 2 | PM |
| gi\|49065830 | 40S ribosomal protein S7 | 22,2 | 4 | PM |
| gi\|49065830 | 40S ribosomal protein S7 | 28,4 | 4 | MT |
| gi\|52788199 | 40S ribosomal protein S9 | 15 | 1 | PM |
| gi\|52788199 | 40S ribosomal protein S9 | 18,6 | 3 | MT |
| gi\|183986025 | 5'-nucleotidase, cytosolic III | 13,8 | 2 | CS |
| gi\|730581 | 60S acidic ribosomal protein P0 | 16,4 | 4 | PM |
| gi\|730581 | 60S acidic ribosomal protein P0 | 23 | 5 | MT |

| # Accession | Protein name | % Cov | Peptides | Fraction |
|---|---|---|---|---|
| gi\|50403574 | 60S ribosomal protein L10 | 29.4 | 2 | PM |
| gi\|730529 | 60S ribosomal protein L13 | 29.4 | 5 | MT |
| gi\|548747 | 60S ribosomal protein L13a | 19.2 | 2 | PM |
| gi\|548747 | 60S ribosomal protein L13a | 13.8 | 2 | MT |
| gi\|2500360 | 60S ribosomal protein L14 | 24.8 | 2 | MT |
| gi\|51338615 | 60S ribosomal protein L23a | 12.2 | 2 | PM |
| gi\|51338615 | 60S ribosomal protein L23a | 20.5 | 3 | MT |
| gi\|51704206 | 60S ribosomal protein L7 | 20.8 | 4 | PM |
| gi\|51704206 | 60S ribosomal protein L7 | 7.3 | 2 | MT |
| gi\|205686171 | 6-phosphogluconolactonase | 39.7 | 3 | CS |
| gi\|91234898 | 84 kDa heat shock protein | 61.9 | 39 | CS |
| gi\|91234898 | 84 kDa heat shock protein | 18.9 | 6 | PM |
| gi\|91234898 | 84 kDa heat shock protein | 26.0 | 11 | MT |
| gi\|33086444 | Ab1-021 | 27.8 | 19 | CS |
| gi\|33086478 | Ab1-205 | 13.4 | 6 | MT |
| gi\|33086478 | Ab1-205 | 13.9 | 4 | PM |
| gi\|33086600 | Ab2-371 | 7.0 | 4 | PM |
| gi\|32264613 | Ac1164 | 23.8 | 8 | PM |
| gi\|32264613 | Ac1164 | 14.7 | 3 | MT |
| gi\|8392836 | Acetyl-CoA acetyltransferase 1 precursor | 31.4 | 9 | CS |
| gi\|8392836 | Acetyl-CoA acetyltransferase 1 precursor | 69.8 | 32 | PM |
| gi\|6978429 | Acetyl-CoA acyltransferase 1 | 16.4 | 2 | MT |
| gi\|149027156 | Acetyl-CoA acyltransferase 2, isoform CRA_f | 82.4 | 36 | CS |
| gi\|149027156 | Acetyl-CoA acyltransferase 2, isoform CRA_f | 72.5 | 36 | PM |
| gi\|94963134 | Acetyl-CoA carboxylase 2 | 5.8 | 1 | CS |
| gi\|60391194 | Aconitate hydratase, mitochondrial | 59.9 | 50 | PM |
| gi\|60391194 | Aconitate hydratase, mitochondrial | 70.8 | 117 | CS |
| gi\|77993370 | Actin α, cardiac 1 | 70.6 | 27 | CS |
| gi\|9506371 | Actin, α 1, skeletal muscle | 78.5 | 84 | MT |
| gi\|77993368 | Acyl-CoA synthetase family member 2 precursor | 48.1 | 27 | CS |
| gi\|77993368 | Acyl-CoA synthetase family member 2 precursor | 28.1 | 10 | PM |
| gi\|77993368 | Acyl-CoA synthetase family member 2 precursor | 10.2 | 3 | MT |
| gi\|157818027 | Acyl-CoA synthetase short-chain family member 1 | 52.8 | 29 | CS |
| gi\|157818027 | Acyl-CoA synthetase short-chain family member 1 | 6.7 | 2 | MT |

| # Accession | Protein name | % Cov | Peptides | Fraction |
|---|---|---|---|---|
| gi\|157818027 | Acyl-CoA synthetase short-chain family member 1 | 21.0 | 6 | PM |
| gi\|157817043 | Acyl-CoA thioesterase 13 | 14.3 | 2 | PM |
| gi\|48675862 | Acyl-CoA thioesterase 2 | 63.6 | 18 | CS |
| gi\|62078649 | Acyl-CoA thioesterase 9 | 16.9 | 4 | CS |
| gi\|62078649 | Acyl-CoA thioesterase 9 | 8.4 | 2 | PM |
| gi\|197313734 | Acyl-CoA dehydrogenase family, member 9 | 37.3 | 10 | CS |
| gi\|197313734 | Acyl-CoA dehydrogenase family, member 9 | 26.2 | 8 | MT |
| gi\|149048722 | Acyl-CoA dehydrogenase family, member 9, isoform CRA_c | 26.5 | 9 | PM |
| gi\|48734846 | Acyl-CoA dehydrogenase, C-2 to C-3 short chain | 61.8 | 19 | CS |
| gi\|48734846 | Acyl-CoA dehydrogenase, C-2 to C-3 short chain | 47.6 | 10 | PM |
| gi\|56541110 | Acyl-CoA dehydrogenase, very long chain | 71.5 | 54 | PM |
| gi\|56541110 | Acyl-CoA dehydrogenase, very long chain | 59.1 | 33 | MT |
| gi\|6166586 | Acyl-CoA thioesterase 2, mitochondrial | 24.9 | 7 | PM |
| gi\|6166586 | Acyl-CoA thioesterase 2, mitochondrial | 22.7 | 5 | MT |
| gi\|188595700 | Acylglycerol kinase | 8.1 | 2 | PM |
| gi\|157822589 | Acylphosphatase 1 | 39.4 | 2 | CS |
| gi\|281332093 | Acylphosphatase 2, muscle type | 42.7 | 4 | CS |
| gi\|157823677 | Adaptor-related protein complex 2, α 1 subunit | 4.7 | 2 | PM |
| gi\|56961624 | Adaptor-related protein complex 2, σ 1 subunit | 14.1 | 2 | PM |
| gi\|61556832 | Adenine phosphoribosyltransferase | 60.0 | 8 | CS |
| gi\|52345435 | Adenosine kinase | 28.0 | 6 | CS |
| gi\|8392878 | Adenosylhomocysteinase | 8.3 | 2 | CS |
| gi\|77020256 | Adenylate kinase 2 isoform b | 40.5 | 8 | PM |
| gi\|77020256 | Adenylate kinase 2 isoform b | 44.0 | 12 | MT |
| gi\|8392885 | Adenylate kinase 3-like 1 | 26.5 | 5 | CS |
| gi\|8918488 | Adenylate kinase isozyme 1 | 28.9 | 4 | PM |
| gi\|8918488 | Adenylate kinase isozyme 1 | 35.6 | 6 | MT |
| gi\|194473622 | Adenylosuccinate lyase | 20.0 | 4 | CS |
| gi\|54035294 | Adh5 protein | 18.5 | 4 | CS |
| gi\|5002228 | Adipocyte lipid-binding protein | 46.7 | 5 | MT |
| gi\|728810 | ADP/ATP translocase 2 | 74.5 | 68 | PM |
| gi\|728810 | ADP/ATP translocase 2 | 79.2 | 70 | MT |
| gi\|149052783 | ADP-ribosylation factor 1, isoform CRA_d | 21.2 | 2 | MT |
| gi\|57033190 | ADP-ribosylation factor 3 | 37.6 | 4 | CS |

| # Accession | Protein name | % Cov | Peptides | Fraction |
|---|---|---|---|---|
| gi\|57033190 | ADP-ribosylation factor 3 | 15.5 | 2 | PM |
| gi\|81890516 | ADP-ribosylation factor-like protein 8B | 23.7 | 3 | PM |
| gi\|58865636 | ADP-ribosyltransferase 3 | 13.9 | 2 | MT |
| gi\|543793 | Afamin | 16.8 | 7 | CS |
| gi\|198442897 | AFG3(ATPase family gene 3)-like 2 | 25.9 | 16 | PM |
| gi\|198442897 | AFG3(ATPase family gene 3)-like 2 | 32.2 | 19 | MT |
| gi\|39930812 | Aflatoxin B1 aldehyde reductase member 2 | 14.2 | 3 | CS |
| gi\|40352787 | Ak3 protein | 58.2 | 7 | CS |
| gi\|197927423 | Aldehyde dehydrogenase 4A1 | 33.1 | 9 | CS |
| gi\|75905479 | Aldehyde dehydrogenase 9A1 | 35.6 | 9 | CS |
| gi\|55605 | Aldehyde dehydrogenase preprotein | 44.9 | 18 | CS |
| gi\|55605 | Aldehyde dehydrogenase preprotein | 43.7 | 13 | PM |
| gi\|399660 | Aldehyde reductase | 23.7 | 4 | CS |
| gi\|158138555 | Aldo-keto reductase family 1, member C-like 1 | 36.1 | 9 | CS |
| gi\|55926139 | α isoform of regulatory subunit A, protein phosphatase 2 | 26.7 | 8 | CS |
| gi\|83816939 | α-1-inhibitor III precursor | 44.8 | 60 | CS |
| gi\|81872093 | α-1-macroglobulin | 28.9 | 24 | CS |
| gi\|58865362 | α-2-antiplasmin precursor | 11.6 | 2 | CS |
| gi\|60552688 | α-2-HS-glycoprotein | 46.6 | 13 | CS |
| gi\|6978477 | α-2-HS-glycoprotein precursor | 7.7 | 1 | PM |
| gi\|744592 | α-B crystallin | 53.7 | 8 | PM |
| gi\|744592 | α-B crystallin | 60.0 | 9 | MT |
| gi\|3462887 | α-fodrin | 35.1 | 48 | PM |
| gi\|206050 | α-propionyl-CoA carboxylase (EC 6.4.1.3) | 17.2 | 9 | PM |
| gi\|6094309 | α-soluble NSF attachment protein | 21.7 | 4 | PM |
| gi\|81884653 | Aminoacylase-1A | 26.7 | 5 | CS |
| gi\|158749540 | Aminopeptidase puromycin sensitive | 15.7 | 7 | CS |
| gi\|157819187 | Amylo-1,6-glucosidase, 4-α-glucanotransferase | 24.9 | 23 | CS |
| gi\|157822539 | Ankyrin 1, erythrocytic | 8.1 | 2 | PM |
| gi\|6978501 | Annexin A1 | 37.6 | 6 | CS |
| gi\|58865414 | Annexin A11 | 35.4 | 11 | CS |
| gi\|58865414 | Annexin A11 | 14.9 | 2 | PM |
| gi\|9845234 | Annexin A2 | 39.5 | 8 | CS |
| gi\|9845234 | Annexin A2 | 45.7 | 10 | PM |

| # Accession | Protein name | % Cov | Peptides | Fraction |
|---|---|---|---|---|
| gi\|9845234 | Annexin A2 | 37.8 | 7 | MT |
| gi\|51980303 | Annexin A3 | 54.9 | 13 | CS |
| gi\|51980303 | Annexin A3 | 18.5 | 3 | PM |
| gi\|51980303 | Annexin A3 | 14.2 | 2 | MT |
| gi\|55742832 | Annexin A4 | 29.8 | 4 | CS |
| gi\|6978505 | Annexin A5 | 41.1 | 10 | PM |
| gi\|6978505 | Annexin A5 | 37.3 | 8 | MT |
| gi\|763181 | Annexin VI | 19.8 | 8 | MT |
| gi\|6103726 | Antioxidant enzyme B166 | 22.5 | 2 | MT |
| gi\|6103726 | Antioxidant enzyme B166 | 22.5 | 2 | PM |
| gi\|73695330 | Ap2b1 protein | 9.4 | 3 | PM |
| gi\|73695330 | Ap2b1 protein | 4.0 | 1 | MT |
| gi\|165971324 | Apoa1bp protein | 14.9 | 2 | CS |
| gi\|6978515 | Apolipoprotein A-I precursor | 16.6 | 2 | PM |
| gi\|60552712 | Apolipoprotein A-IV | 16.9 | 2 | CS |
| gi\|195540026 | Apolipoprotein B mRNA editing enzyme, catalytic polypeptide-like 2 | 11.6 | 1 | CS |
| gi\|71051103 | Apolipoprotein C-I | 18.2 | 1 | PM |
| gi\|149041554 | Apolipoprotein C-III, isoform CRA_a | 46.0 | 2 | PM |
| gi\|295916 | Apolipoprotein E | 19.9 | 2 | PM |
| gi\|55824759 | Apolipoprotein E | 18.0 | 2 | MT |
| gi\|57528174 | Apolipoprotein H (β-2-glycoprotein I) | 23.2 | 5 | PM |
| gi\|62078943 | Apolipoprotein O-like | 44.1 | 9 | PM |
| gi\|62078943 | Apolipoprotein O-like | 47.2 | 8 | MT |
| gi\|7619915 | Apoptosis-inducing factor | 55.7 | 21 | PM |
| gi\|7619915 | Apoptosis-inducing factor | 52.9 | 21 | MT |
| gi\|6978527 | Aquaporin 1 | 19.3 | 5 | PM |
| gi\|38512104 | Arginyl aminopeptidase (aminopeptidase B) | 12.3 | 5 | CS |
| gi\|6980972 | Aspartate aminotransferase 2 | 81.6 | 65 | CS |
| gi\|6980972 | Aspartate aminotransferase 2 | 70.5 | 35 | PM |
| gi\|67846036 | Aspartyl aminopeptidase | 9.5 | 1 | CS |
| gi\|55249793 | Asph protein | 30.4 | 6 | PM |
| gi\|55249793 | Asph protein | 23.7 | 4 | MT |
| gi\|54145376 | ATP synthase F0 subunit 8 | 59.7 | 5 | PM |
| gi\|54145376 | ATP synthase F0 subunit 8 | 55.2 | 5 | MT |

| # Accession | Protein name | % Cov | Peptides | Fraction |
|---|---|---|---|---|
| gi\|157822047 | ATP synthase mitochondrial F1 complex assembly factor 2 | 14.1 | 2 | CS |
| gi\|83300587 | ATP synthase subunit α, mitochondrial | 79.0 | 189 | MT |
| gi\|461587 | ATP synthase subunit e, mitochondrial | 84.5 | 9 | PM |
| gi\|461587 | ATP synthase subunit e, mitochondrial | 84.5 | 10 | MT |
| gi\|543880 | ATP synthase subunit O, mitochondrial | 74.7 | 28 | PM |
| gi\|39645769 | ATP synthase, H+ transporting, mitochondrial F0 complex, subunit B1 | 59.8 | 39 | PM |
| gi\|39645769 | ATP synthase, H+ transporting, mitochondrial F0 complex, subunit B1 | 58.6 | 38 | MT |
| gi\|9506411 | ATP synthase, H+ transporting, mitochondrial F0 complex, subunit d | 90.1 | 52 | PM |
| gi\|9506411 | ATP synthase, H+ transporting, mitochondrial F0 complex, subunit d | 91.3 | 44 | MT |
| gi\|47058994 | ATP synthase, H+ transporting, mitochondrial F0 complex, subunit G | 65.1 | 9 | PM |
| gi\|47058994 | ATP synthase, H+ transporting, mitochondrial F0 complex, subunit G | 69.9 | 17 | MT |
| gi\|71681130 | ATP synthase, H+ transporting, mitochondrial F1 complex, β polypeptide | 79.6 | 165 | PM |
| gi\|149034587 | ATP synthase, H+ transporting, mitochondrial F1 complex, δ subunit, isoform CRA_b | 74.4 | 21 | PM |
| gi\|149034587 | ATP synthase, H+ transporting, mitochondrial F1 complex, δ subunit, isoform CRA_b | 59.5 | 28 | MT |
| gi\|34786049 | ATP synthase, H+ transporting, mitochondrial F1 complex, ε subunit | 82.4 | 4 | PM |
| gi\|34786049 | ATP synthase, H+ transporting, mitochondrial F1 complex, ε subunit | 92.2 | 4 | MT |
| gi\|39930503 | ATP synthase, H+ transporting, mitochondrial F1 complex, γ subunit | 59.4 | 36 | MT |
| gi\|149059861 | ATP synthase, H+ transporting, mitochondrial F1 complex, O subunit, isoform CRA_c | 78.1 | 21 | MT |
| gi\|81908923 | ATPase family AAA domain-containing protein 1 | 18.8 | 3 | PM |
| gi\|81908923 | ATPase family AAA domain-containing protein 1 | 20.2 | 2 | MT |
| gi\|77917538 | ATPase family, AAA domain containing 3A | 28.3 | 7 | PM |
| gi\|77917538 | ATPase family, AAA domain containing 3A | 26.6 | 8 | MT |
| gi\|77917528 | ATPase inhibitory factor 1 precursor | 74.8 | 5 | PM |
| gi\|77917528 | ATPase inhibitory factor 1 precursor | 72.0 | 6 | MT |
| gi\|50927657 | ATPase, Na+/K+ transporting, β 1 polypeptide | 39.1 | 13 | PM |
| gi\|50927657 | ATPase, Na+/K+ transporting, β 1 polypeptide | 34.2 | 9 | MT |
| gi\|71152214 | ATP-binding cassette sub-family B member 7, mitochondrial | 8.1 | 3 | PM |
| gi\|71152214 | ATP-binding cassette sub-family B member 7, mitochondrial | 7.4 | 4 | MT |
| gi\|81910043 | ATP-binding cassette sub-family B member 8, mitochondrial | 15.6 | 4 | PM |
| gi\|81910043 | ATP-binding cassette sub-family B member 8, mitochondrial | 14.9 | 5 | MT |
| gi\|149044979 | AU RNA binding protein/enoyl-coenzyme A hydratase (predicted), isoform CRA_a | 22.9 | 3 | PM |
| gi\|33086642 | Ba2-693 | 11.2 | 6 | CS |
| gi\|81917434 | Basal cell adhesion molecule | 7.5 | 3 | PM |
| gi\|7709992 | Basigin isoform 2 | 43.0 | 11 | PM |

| # Accession | Protein name | % Cov | Peptides | Fraction |
|---|---|---|---|---|
| gi\|7709992 | Basigin isoform 2 | 28.7 | 7 | MT |
| gi\|38512141 | Bcat2 protein | 6.2 | 2 | PM |
| gi\|51948420 | B-cell receptor-associated protein 31 | 6.1 | 1 | PM |
| gi\|51948420 | B-cell receptor-associated protein 31 | 12.7 | 2 | MT |
| gi\|9789466 | BCL2/adenovirus E1B 19 kDa-interacting protein 3 | 13.9 | 2 | PM |
| gi\|9789466 | BCL2/adenovirus E1B 19 kDa-interacting protein 3 | 11.8 | 2 | MT |
| gi\|56090628 | BCS1-like | 8.1 | 3 | PM |
| gi\|56090628 | BCS1-like | 23.4 | 5 | MT |
| gi\|55575 | β-actin | 55.7 | 17 | CS |
| gi\|55575 | β-actin | 62.4 | 29 | PM |
| gi\|56260 | β-globin | 81.0 | 58 | CS |
| gi\|984679 | β-globin | 86.4 | 75 | CS |
| gi\|56252 | β-globin | 84.4 | 19 | PM |
| gi\|984679 | β-globin | 83.0 | 22 | PM |
| gi\|984679 | β-globin | 85.7 | 18 | MT |
| gi\|81888008 | β-lactamase-like protein 2 | 25.4 | 4 | CS |
| gi\|50925455 | Biliverdin reductase A | 20.7 | 3 | CS |
| gi\|81295385 | Biphenyl hydrolase-like (serine hydrolase) | 22.7 | 2 | CS |
| gi\|81866115 | Bone marrow stromal antigen 2 | 11.6 | 3 | PM |
| gi\|81866115 | Bone marrow stromal antigen 2 | 12.8 | 2 | MT |
| gi\|158187544 | Brain glycogen phosphorylase | 64.3 | 47 | CS |
| gi\|730248 | Brain protein 44 | 32.3 | 2 | PM |
| gi\|730248 | Brain protein 44 | 40.9 | 4 | MT |
| gi\|66911415 | Brain protein 44-like | 26.6 | 3 | PM |
| gi\|66911415 | Brain protein 44-like | 26.6 | 3 | MT |
| gi\|38142418 | Brain Y-box binding protein 1 | 19.6 | 2 | MT |
| gi\|77736548 | Branched chain keto acid dehydrogenase E1, α polypeptide | 25.1 | 6 | PM |
| gi\|3023378 | Branched-chain-amino-acid aminotransferase, mitochondrial | 35.1 | 8 | CS |
| gi\|60688421 | C9 protein | 10.1 | 1 | CS |
| gi\|55249666 | Cadherin 13 | 18.1 | 6 | PM |
| gi\|55249666 | Cadherin 13 | 6.3 | 1 | MT |
| gi\|871525 | Calcium binding protein | 29.9 | 5 | PM |
| gi\|49522734 | Calcium regulated heat stable protein 1 | 21.1 | 1 | CS |
| gi\|4753894 | Calcyclin | 25.8 | 2 | CS |

| # Accession | Protein name | % Cov | Peptides | Fraction |
|---|---|---|---|---|
| gi\|8394168 | Calmodulin 2 | 51.7 | 2 | CS |
| gi\|8394168 | Calmodulin 2 | 72.5 | 6 | PM |
| gi\|8394168 | Calmodulin 2 | 26.9 | 3 | MT |
| gi\|543922 | Calnexin | 30.0 | 14 | PM |
| gi\|543922 | Calnexin | 31.0 | 11 | MT |
| gi\|83301638 | Calpain small subunit 1 | 15.9 | 3 | CS |
| gi\|77540019 | Calpastatin | 12.2 | 4 | CS |
| gi\|988307 | Calsequestrin | 14.0 | 3 | CS |
| gi\|78099764 | Calsequestrin-2 | 43.1 | 22 | PM |
| gi\|78099764 | Calsequestrin-2 | 34.1 | 19 | MT |
| gi\|158186676 | Calumenin isoform a | 8.9 | 3 | PM |
| gi\|76559925 | Calumenin isoform b | 11.1 | 3 | CS |
| gi\|155369271 | cAMP-dependent protein kinase catalytic subunit α | 19.9 | 4 | CS |
| gi\|83304285 | cAMP-dependent protein kinase type II-α regulatory subunit | 11.7 | 2 | CS |
| gi\|74355722 | Capping protein (actin filament) muscle Z-line, α 2 | 17.1 | 3 | MT |
| gi\|205687264 | Carbonic anhydrase 1 | 51.0 | 10 | CS |
| gi\|9506445 | Carbonic anhydrase II | 54.2 | 8 | CS |
| gi\|157818703 | Carbonic anhydrase XIV | 9.8 | 2 | PM |
| gi\|157818703 | Carbonic anhydrase XIV | 9.8 | 2 | MT |
| gi\|9506467 | Carbonyl reductase 1 | 49.8 | 9 | CS |
| gi\|205495 | Cardiac myosin light chain 2 | 68.1 | 8 | CS |
| gi\|46577628 | Cardiac phospholamban | 32.7 | 2 | PM |
| gi\|46577628 | Cardiac phospholamban | 34.6 | 2 | MT |
| gi\|59797483 | Carnitine O-acetyltransferase | 8.1 | 2 | CS |
| gi\|59797483 | Carnitine O-acetyltransferase | 24.1 | 11 | PM |
| gi\|59797483 | Carnitine O-acetyltransferase | 8.3 | 4 | MT |
| gi\|6978705 | Carnitine O-palmitoyltransferase precursor | 59.6 | 27 | PM |
| gi\|6978705 | Carnitine O-palmitoyltransferase precursor | 41.3 | 19 | MT |
| gi\|48735405 | Carnitine palmitoyltransferase 1a, liver | 11.3 | 4 | PM |
| gi\|48735405 | Carnitine palmitoyltransferase 1a, liver | 9.8 | 3 | MT |
| gi\|6978607 | Catalase | 22.0 | 4 | CS |
| gi\|6978607 | Catalase | 18.8 | 7 | PM |
| gi\|6978607 | Catalase | 28.5 | 10 | MT |
| gi\|82830420 | Cathepsin B preproprotein | 9.1 | 2 | PM |

| # Accession | Protein name | % Cov | Peptides | Fraction |
|---|---|---|---|---|
| gi\|27465583 | Cationic trypsinogen precursor | 10.9 | 2 | CS |
| gi\|575380 | Caveolin 1 | 27.4 | 6 | MT |
| gi\|45267819 | Caveolin 2 | 21.0 | 3 | PM |
| gi\|9506465 | Caveolin 3 | 37.1 | 3 | PM |
| gi\|6978635 | CD59 molecule, complement regulatory protein precursor | 23.0 | 2 | PM |
| gi\|6978639 | Cd81 molecule | 27.5 | 3 | PM |
| gi\|215275245 | CDGSH iron sulfur domain-containing protein 1 | 44.4 | 8 | PM |
| gi\|215275245 | CDGSH iron sulfur domain-containing protein 1 | 51.9 | 5 | MT |
| gi\|6531681 | Cell division cycle 42 | 20.9 | 4 | CS |
| gi\|6531681 | Cell division cycle 42 | 30.9 | 4 | PM |
| gi\|6531681 | Cell division cycle 42 | 16.8 | 3 | MT |
| gi\|6707016 | Cell surface protein CD36 | 39.2 | 13 | PM |
| gi\|6707016 | Cell surface protein CD36 | 28.8 | 10 | MT |
| gi\|213512607 | Citrate lyase β like precursor | 26.3 | 7 | CS |
| gi\|56388799 | Ckb protein | 67.4 | 42 | CS |
| gi\|56388799 | Ckb protein | 46.6 | 14 | PM |
| gi\|56388799 | Ckb protein | 35.8 | 13 | MT |
| gi\|9506497 | Clathrin, heavy chain (Hc) | 10.9 | 5 | CS |
| gi\|9506497 | Clathrin, heavy chain (Hc) | 13.9 | 9 | PM |
| gi\|38181879 | Clu protein | 8.9 | 2 | MT |
| gi\|8393101 | Cofilin 1 | 55.4 | 8 | CS |
| gi\|149051244 | Cofilin 2, muscle (predicted), isoform CRA_b | 56.0 | 8 | CS |
| gi\|62078929 | Coiled-coil domain containing 51 | 26.9 | 4 | PM |
| gi\|62078929 | Coiled-coil domain containing 51 | 20.6 | 3 | MT |
| gi\|157817027 | Coiled-coil-helix-coiled-coil-helix domain containing 3 | 66.1 | 18 | PM |
| gi\|157817027 | Coiled-coil-helix-coiled-coil-helix domain containing 3 | 66.1 | 18 | MT |
| gi\|259016391 | Collagen α-1(I) chain | 34.3 | 20 | MT |
| gi\|281427229 | Collagen, type VI, α 2 | 10.3 | 5 | MT |
| gi\|48675371 | Complement component 1, q subcomponent binding protein precursor | 33.3 | 6 | CS |
| gi\|48675371 | Complement component 1, q subcomponent binding protein precursor | 39.4 | 7 | PM |
| gi\|48675371 | Complement component 1, q subcomponent binding protein precursor | 17.9 | 4 | MT |
| gi\|158138561 | Complement component 3 | 44.8 | 53 | CS |
| gi\|158138561 | Complement component 3 | 10.6 | 6 | PM |
| gi\|46237589 | Complement component 4, gene 1 | 4.6 | 2 | CS |

| # Accession | Protein name | % Cov | Peptides | Fraction |
|---|---|---|---|---|
| gi\|56268879 | Complement factor B | 8.4 | 4 | CS |
| gi\|74353709 | Cox15 protein | 9.4 | 2 | PM |
| gi\|74353709 | Cox15 protein | 17.2 | 3 | MT |
| gi\|34849861 | Cox6c protein | 67.1 | 15 | PM |
| gi\|34849861 | Cox6c protein | 69.7 | 9 | MT |
| gi\|187469733 | Cox7a2l protein | 72.6 | 6 | PM |
| gi\|187469733 | Cox7a2l protein | 61.1 | 6 | MT |
| gi\|847719 | CPTI like protein | 50.9 | 30 | PM |
| gi\|847719 | CPTI like protein | 46.2 | 26 | MT |
| gi\|67460125 | Cullin-associated NEDD8-dissociated protein 2 | 8.6 | 2 | CS |
| gi\|737713 | Cys-rich protein CRP2 | 17.3 | 3 | MT |
| gi\|737713 | Cys-rich protein CRP2 | 42.8 | 6 | CS |
| gi\|54036889 | Cysteine-rich protein 1 | 13.0 | 1 | CS |
| gi\|829020 | Cytochrome B gene | 4.2 | 3 | PM |
| gi\|38303959 | Cytochrome b5 reductase 3 | 38.9 | 8 | PM |
| gi\|38303959 | Cytochrome b5 reductase 3 | 29.2 | 7 | MT |
| gi\|48735409 | Cytochrome b5 type B (outer mitochondrial membrane) | 65.8 | 7 | PM |
| gi\|149015875 | Cytochrome b-5, isoform CRA_e | 49.6 | 5 | PM |
| gi\|149015875 | Cytochrome b-5, isoform CRA_e | 52.5 | 5 | MT |
| gi\|81884378 | Cytochrome b-c1 complex subunit 1, mitochondrial | 66.9 | 65 | PM |
| gi\|62511137 | Cytochrome b-c1 complex subunit 6, mitochondrial | 70.8 | 18 | MT |
| gi\|81865392 | Cytochrome b-c1 complex subunit 8 | 54.9 | 4 | PM |
| gi\|81865392 | Cytochrome b-c1 complex subunit 8 | 57.3 | 6 | MT |
| gi\|5851903 | Cytochrome C oxidase assembly protein COX17 | 36.5 | 2 | CS |
| gi\|54145374 | Cytochrome c oxidase subunit 1 | 7.6 | 3 | PM |
| gi\|54145375 | Cytochrome c oxidase subunit 2 | 47.6 | 10 | PM |
| gi\|26983979 | Cytochrome c oxidase subunit II | 36.6 | 15 | MT |
| gi\|8393180 | Cytochrome c oxidase subunit IV isoform 1 precursor | 69.2 | 39 | PM |
| gi\|8393180 | Cytochrome c oxidase subunit IV isoform 1 precursor | 64.5 | 38 | MT |
| gi\|55971 | Cytochrome c oxidase subunit Va preprotein | 70.6 | 56 | PM |
| gi\|55971 | Cytochrome c oxidase subunit Va preprotein | 69.2 | 50 | MT |
| gi\|55992 | Cytochrome c oxidase subunit VIa (AA 1 - 118) | 67.5 | 32 | PM |
| gi\|55992 | Cytochrome c oxidase subunit VIa (AA 1 - 118) | 61.5 | 38 | MT |
| gi\|56025 | Cytochrome c oxidase subunit VIIa | 37.4 | 7 | PM |

| # Accession | Protein name | % Cov | Peptides | Fraction |
|---|---|---|---|---|
| gi\|56025 | Cytochrome c oxidase subunit VIIa | 37.4 | 9 | MT |
| gi\|65301490 | Cytochrome c oxidase subunit VIIb precursor | 16.3 | 2 | PM |
| gi\|65301490 | Cytochrome c oxidase subunit VIIb precursor | 16.3 | 1 | MT |
| gi\|77736544 | Cytochrome c oxidase, subunit VIa, polypeptide 1 precursor | 69.4 | 7 | PM |
| gi\|77736544 | Cytochrome c oxidase, subunit VIa, polypeptide 1 precursor | 44.1 | 6 | MT |
| gi\|223718723 | Cytochrome c oxidase, subunit VIb polypeptide 1 | 81.4 | 33 | PM |
| gi\|197927439 | Cytochrome c oxidase, subunit VIIc | 36.5 | 11 | PM |
| gi\|197927439 | Cytochrome c oxidase, subunit VIIc | 39.7 | 7 | MT |
| gi\|6978725 | Cytochrome c, somatic | 49.5 | 10 | CS |
| gi\|6978725 | Cytochrome c, somatic | 73.3 | 22 | PM |
| gi\|6978725 | Cytochrome c, somatic | 72.4 | 14 | MT |
| gi\|149066116 | Cytochrome c-1 (predicted), isoform CRA_c | 47.6 | 45 | PM |
| gi\|149066116 | Cytochrome c-1 (predicted), isoform CRA_c | 54.9 | 45 | MT |
| gi\|829025 | Cytochrome oxidase III | 7.7 | 3 | PM |
| gi\|729378 | Cytoplasmic dynein 1 heavy chain 1 | 5.9 | 4 | PM |
| gi\|157823877 | Cytoskeleton-associated protein 4 | 11.0 | 2 | PM |
| gi\|157823877 | Cytoskeleton-associated protein 4 | 19.0 | 3 | MT |
| gi\|81884348 | Cytosol aminopeptidase | 22.9 | 5 | CS |
| gi\|537941 | Cytosolic NADP-dependent isocitrate dehydrogenase | 37.9 | 11 | CS |
| gi\|68837285 | D-β-hydroxybutyrate dehydrogenase, mitochondrial | 41.1 | 11 | PM |
| gi\|68837285 | D-β-hydroxybutyrate dehydrogenase, mitochondrial | 41.1 | 13 | MT |
| gi\|56057 | Decorin | 21.8 | 5 | PM |
| gi\|56057 | Decorin | 29.7 | 5 | MT |
| gi\|457929 | δ subunit of F1F0 ATPase | 33.9 | 2 | CS |
| gi\|6015047 | δ(3,5)-δ(2,4)-dienoyl-CoA isomerase, mitochondrial | 47.4 | 6 | CS |
| gi\|157819621 | Deoxyguanosine kinase | 20.5 | 3 | CS |
| gi\|38197676 | Desmin | 8.7 | 2 | CS |
| gi\|149045175 | Desmoplakin, isoform CRA_b | 13.4 | 4 | MT |
| gi\|56605642 | Diablo | 14.8 | 3 | MT |
| gi\|54261671 | Diazepam binding inhibitor (GABA receptor modulator, acyl-Coenzyme A binding protein) | 73.6 | 5 | CS |
| gi\|54261671 | Diazepam binding inhibitor (GABA receptor modulator, acyl-Coenzyme A binding protein) | 58.6 | 2 | PM |
| gi\|54261671 | Diazepam binding inhibitor (GABA receptor modulator, acyl-Coenzyme A binding protein) | 51.7 | 2 | MT |
| gi\|183985854 | Dihydrolipoamide branched chain transacylase E2 | 8.5 | 4 | PM |
| gi\|81885266 | Dihydrolipoyl dehydrogenase, mitochondrial | 44.0 | 15 | CS |

| # Accession | Protein name | % Cov | Peptides | Fraction |
|---|---|---|---|---|
| gi\|81885266 | Dihydrolipoyl dehydrogenase, mitochondrial | 48.1 | 18 | PM |
| gi\|81885266 | Dihydrolipoyl dehydrogenase, mitochondrial | 23.0 | 7 | MT |
| gi\|6012071 | Dithiolethione-inducible gene-1 | 31.9 | 8 | CS |
| gi\|84370227 | DnaJ (Hsp40) homolog, subfamily A, member 3 isoform 1 | 27.9 | 7 | PM |
| gi\|84370227 | DnaJ (Hsp40) homolog, subfamily A, member 3 isoform 1 | 27.5 | 7 | MT |
| gi\|70794764 | DnaJ (Hsp40) homolog, subfamily A, member 4 | 9.9 | 1 | CS |
| gi\|171847074 | DnaJ (Hsp40) homolog, subfamily C, member 11 | 9.7 | 3 | PM |
| gi\|171847074 | DnaJ (Hsp40) homolog, subfamily C, member 11 | 16.6 | 4 | MT |
| gi\|62512124 | Dolichyl-diphosphooligosaccharide--protein glycosyltransferase subunit 2 | 5.7 | 2 | PM |
| gi\|62512124 | Dolichyl-diphosphooligosaccharide--protein glycosyltransferase subunit 2 | 19.5 | 4 | MT |
| gi\|294543 | Dynein heavy chain | 6.9 | 3 | MT |
| gi\|157823277 | Dysferlin | 9.6 | 7 | PM |
| gi\|157823277 | Dysferlin | 5.9 | 2 | MT |
| gi\|149021348 | EF hand domain family, member A2 | 8.0 | 1 | MT |
| gi\|81910618 | EH domain-containing protein 1 | 10.7 | 3 | MT |
| gi\|81908709 | EH domain-containing protein 2 | 22.1 | 8 | PM |
| gi\|81908709 | EH domain-containing protein 2 | 21.4 | 8 | MT |
| gi\|53237076 | EH-domain containing 4 | 8.5 | 2 | PM |
| gi\|53237076 | EH-domain containing 4 | 15.0 | 3 | MT |
| gi\|81884360 | Electron transfer flavoprotein subunit β | 63.1 | 26 | CS |
| gi\|81884360 | Electron transfer flavoprotein subunit β | 57.3 | 14 | PM |
| gi\|81884360 | Electron transfer flavoprotein subunit β | 47.5 | 9 | MT |
| gi\|57527204 | Electron-transfer-flavoprotein, α polypeptide precursor | 77.8 | 35 | CS |
| gi\|57527204 | Electron-transfer-flavoprotein, α polypeptide precursor | 70.0 | 19 | PM |
| gi\|57527204 | Electron-transfer-flavoprotein, α polypeptide precursor | 46.6 | 12 | MT |
| gi\|52138635 | Electron-transferring-flavoprotein dehydrogenase precursor | 57.8 | 40 | PM |
| gi\|52138635 | Electron-transferring-flavoprotein dehydrogenase precursor | 58.8 | 43 | MT |
| gi\|50402096 | Elongation factor 1-α 2 | 44.1 | 14 | CS |
| gi\|50402096 | Elongation factor 1-α 2 | 38.4 | 10 | PM |
| gi\|50402096 | Elongation factor 1-α 2 | 24.0 | 4 | MT |
| gi\|190359305 | Elongation factor Tu, mitochondrial | 62.4 | 21 | PM |
| gi\|190359305 | Elongation factor Tu, mitochondrial | 44.0 | 12 | MT |
| gi\|77917570 | Endonuclease G | 23.8 | 4 | MT |
| gi\|38649320 | Eno1 protein | 24.6 | 6 | PM |

| # Accession | Protein name | % Cov | Peptides | Fraction |
|---|---|---|---|---|
| gi\|59808815 | Enolase 1, α | 26.5 | 6 | MT |
| gi\|54035288 | Enolase 3, β, muscle | 22.4 | 6 | PM |
| gi\|54035288 | Enolase 3, β, muscle | 28.3 | 5 | MT |
| gi\|157821153 | Enoyl-CoA hydratase domain containing 2 | 21.6 | 1 | PM |
| gi\|82395853 | Epidermal fatty acid binding protein 5 | 40.6 | 2 | CS |
| gi\|229485399 | Erlin-2 | 13.0 | 3 | PM |
| gi\|197246046 | Erythrocyte protein band 4.2 | 6.1 | 2 | PM |
| gi\|149051496 | Erythroid spectrin β | 25.0 | 32 | PM |
| gi\|83302472 | ES1 protein homolog, mitochondrial | 63.2 | 17 | CS |
| gi\|83302472 | ES1 protein homolog, mitochondrial | 43.2 | 10 | PM |
| gi\|83302472 | ES1 protein homolog, mitochondrial | 40.2 | 6 | MT |
| gi\|57529187 | Esterase 1 | 22.2 | 7 | CS |
| gi\|81889423 | Eukaryotic initiation factor 4A-II | 23.6 | 4 | CS |
| gi\|85057089 | Eukaryotic translation elongation factor 1 α 1 | 27.1 | 9 | CS |
| gi\|71051349 | Eukaryotic translation elongation factor 1 γ | 16.0 | 4 | CS |
| gi\|8393296 | Eukaryotic translation elongation factor 2 | 8.4 | 2 | PM |
| gi\|8393296 | Eukaryotic translation elongation factor 2 | 10.0 | 2 | MT |
| gi\|88909158 | Eukaryotic translation initiation factor 3 subunit B | 8.3 | 2 | MT |
| gi\|40786436 | Eukaryotic translation initiation factor 4A1 | 28.3 | 3 | CS |
| gi\|91207082 | Eukaryotic translation initiation factor 5A-1 | 55.8 | 4 | CS |
| gi\|91207082 | Eukaryotic translation initiation factor 5A-1 | 20.1 | 2 | PM |
| gi\|81883744 | Evolutionarily conserved signaling intermediate in Toll pathway, mitochondrial | 18.4 | 3 | PM |
| gi\|54261546 | Fabp4 protein | 76.5 | 23 | CS |
| gi\|54261546 | Fabp4 protein | 37.9 | 7 | PM |
| gi\|81883689 | F-actin-capping protein subunit β | 15.4 | 2 | MT |
| gi\|72255551 | Family with sequence similarity 82, member B | 19.4 | 5 | CS |
| gi\|72255551 | Family with sequence similarity 82, member B | 18.4 | 4 | PM |
| gi\|72255551 | Family with sequence similarity 82, member B | 23.2 | 7 | MT |
| gi\|204080 | Fatty acid binding protein | 93.2 | 56 | CS |
| gi\|204080 | Fatty acid binding protein | 96.2 | 19 | PM |
| gi\|157823017 | Ferrochelatase | 12.1 | 2 | PM |
| gi\|157823017 | Ferrochelatase | 13.3 | 2 | MT |
| gi\|6562849 | Fetuin-like protein IRL685 | 23.0 | 8 | CS |
| gi\|4959650 | Fibrillin-1 | 4.4 | 4 | PM |

| # Accession | Protein name | % Cov | Peptides | Fraction |
|---|---|---|---|---|
| gi\|4959650 | Fibrillin-1 | 4.1 | 2 | MT |
| gi\|790487 | Fibrinogen α-E subunit | 27.0 | 12 | CS |
| gi\|790487 | Fibrinogen α-E subunit | 9.7 | 2 | PM |
| gi\|56971493 | Fibrinogen β chain | 42.2 | 9 | CS |
| gi\|56971493 | Fibrinogen β chain | 17.5 | 3 | MT |
| gi\|61098186 | Fibrinogen γ chain | 34.8 | 9 | CS |
| gi\|206725535 | FK506 binding protein 3, 25kDa | 10.3 | 1 | PM |
| gi\|6978487 | Fructose-bisphosphate aldolase A | 84.9 | 52 | CS |
| gi\|6978487 | Fructose-bisphosphate aldolase A | 54.7 | 18 | PM |
| gi\|56541238 | Fumarate hydratase 1 | 44.8 | 13 | PM |
| gi\|73919838 | Fumarylacetoacetate hydrolase domain-containing protein 1 | 27.2 | 2 | CS |
| gi\|229784139 | Fumarylacetoacetate hydrolase domain-containing protein 2 | 15.3 | 3 | CS |
| gi\|51571949 | G1 to S phase transition 1 | 7.4 | 2 | CS |
| gi\|197927125 | Galectin-related protein | 24.4 | 2 | CS |
| gi\|55741776 | γ sarcoglycan | 9.6 | 2 | PM |
| gi\|1183937 | γ-fibrinogen | 9.4 | 2 | PM |
| gi\|6978896 | Gap junction protein, α 1 | 30.9 | 10 | PM |
| gi\|71534276 | GDP dissociation inhibitor 1 | 30.0 | 4 | CS |
| gi\|77799118 | GIMAP4 | 18.0 | 3 | CS |
| gi\|62945328 | Glioblastoma amplified sequence | 34.2 | 8 | CS |
| gi\|62945328 | Glioblastoma amplified sequence | 54.8 | 14 | PM |
| gi\|62945328 | Glioblastoma amplified sequence | 38.4 | 8 | MT |
| gi\|62078447 | Globin, α | 73.2 | 10 | CS |
| gi\|81892272 | Glucose-6-phosphate isomerase | 59.7 | 35 | CS |
| gi\|81892272 | Glucose-6-phosphate isomerase | 14.9 | 4 | PM |
| gi\|81892272 | Glucose-6-phosphate isomerase | 9.7 | 2 | MT |
| gi\|92090591 | Glutamate dehydrogenase 1, mitochondrial | 39.3 | 14 | CS |
| gi\|38197390 | Glutamic-oxaloacetic transaminase 1, soluble (aspartate aminotransferase 1) | 30.8 | 8 | PM |
| gi\|38197390 | Glutamic-oxaloacetic transaminase 1, soluble (aspartate aminotransferase 1) | 34.9 | 9 | MT |
| gi\|66910891 | Glutamic-pyruvate transaminase (alanine aminotransferase) | 11.1 | 1 | CS |
| gi\|78187979 | Glutaredoxin 3 | 31.5 | 5 | CS |
| gi\|68138297 | Glutathione peroxidase | 83.6 | 14 | CS |
| gi\|90903249 | Glutathione peroxidase 4 isoform A precursor | 14.7 | 2 | PM |
| gi\|7188365 | Glutathione S-transferase α | 16.4 | 2 | CS |

| # Accession | Protein name | % Cov | Peptides | Fraction |
|---|---|---|---|---|
| gi\|208969735 | Glutathione S-transferase α 3 | 17.1 | 2 | CS |
| gi\|208969713 | Glutathione S-transferase α 4 | 26.1 | 4 | CS |
| gi\|28933457 | Glutathione S-transferase μ 2 | 82.1 | 18 | CS |
| gi\|28933457 | Glutathione S-transferase μ 2 | 20.6 | 2 | PM |
| gi\|28933457 | Glutathione S-transferase μ 2 | 37.2 | 3 | MT |
| gi\|56090550 | Glutathione S-transferase ω 1 | 23.7 | 3 | CS |
| gi\|529588 | Glutathione S-transferase Yb3 subunit | 67.9 | 10 | CS |
| gi\|9798638 | Glyceraldehyde-3-phosphate dehydrogenase | 60.7 | 23 | PM |
| gi\|9798638 | Glyceraldehyde-3-phosphate dehydrogenase | 61.9 | 17 | MT |
| gi\|57527919 | Glycerol-3-phosphate dehydrogenase 1 (soluble) | 33.5 | 7 | CS |
| gi\|6980978 | Glycerol-3-phosphate dehydrogenase 2, mitochondrial precursor | 8.4 | 1 | MT |
| gi\|204421 | Glycogen phosphorylase | 12.5 | 4 | PM |
| gi\|204421 | Glycogen phosphorylase | 11.9 | 4 | MT |
| gi\|6225463 | Glycogenin-1 | 14.4 | 3 | CS |
| gi\|37589607 | Glycoprotein, synaptic 2 | 9.7 | 3 | PM |
| gi\|37589607 | Glycoprotein, synaptic 2 | 5.8 | 1 | MT |
| gi\|506417 | Glypican | 19.2 | 6 | PM |
| gi\|506417 | Glypican | 8.2 | 3 | MT |
| gi\|8928123 | GMP reductase 1 | 25.2 | 3 | CS |
| gi\|253970435 | GNAS complex locus XLas | 10.1 | 6 | PM |
| gi\|253970435 | GNAS complex locus XLas | 7.9 | 4 | MT |
| gi\|149038203 | Golgi apparatus protein 1 | 7.5 | 4 | PM |
| gi\|6970046 | GPI-anchored ceruloplasmin | 17.3 | 11 | CS |
| gi\|8927570 | G-protein β-2 subunit | 14.7 | 3 | PM |
| gi\|51260133 | Group specific component | 28.8 | 5 | CS |
| gi\|81883733 | Growth hormone-inducible transmembrane protein | 11.9 | 2 | MT |
| gi\|67678103 | GrpE-like 1, mitochondrial | 53.0 | 6 | CS |
| gi\|67678103 | GrpE-like 1, mitochondrial | 12.9 | 2 | PM |
| gi\|51338593 | GTP-binding nuclear protein Ran | 35.2 | 5 | CS |
| gi\|9837357 | GTP-binding protein RAB11B | 31.7 | 5 | CS |
| gi\|9837357 | GTP-binding protein RAB11B | 33.0 | 7 | PM |
| gi\|9837357 | GTP-binding protein RAB11B | 28.9 | 5 | MT |
| gi\|9837359 | GTP-binding protein RAB7 | 32.9 | 4 | PM |
| gi\|9837359 | GTP-binding protein RAB7 | 18.4 | 3 | MT |

| # Accession | Protein name | % Cov | Peptides | Fraction |
|---|---|---|---|---|
| gi\|9910706 | Guanine deaminase | 30.6 | 8 | CS |
| gi\|71089913 | Guanine nucleotide binding protein α inhibiting 2 | 42.5 | 11 | PM |
| gi\|71089913 | guanine nucleotide binding protein α inhibiting 2 | 19.4 | 3 | MT |
| gi\|54037164 | Guanine nucleotide-binding protein subunit β-2-like 1 | 36.6 | 4 | PM |
| gi\|54037164 | Guanine nucleotide-binding protein subunit β-2-like 1 | 45.7 | 5 | MT |
| gi\|56346 | H2B histone | 46.4 | 4 | MT |
| gi\|157823683 | HD domain containing 2 | 15.6 | 1 | CS |
| gi\|157819889 | HD domain containing 3 | 24.6 | 3 | CS |
| gi\|6981052 | Heat shock 10 kDa protein 1 | 32.4 | 2 | PM |
| gi\|55977739 | Heat shock 70 kDa protein 1A/1B | 22.5 | 9 | PM |
| gi\|81886881 | Heat shock 70 kDa protein 4 | 35.6 | 16 | CS |
| gi\|56383 | Heat shock protein (hsp60) precursor | 49.7 | 21 | PM |
| gi\|56383 | Heat shock protein (hsp60) precursor | 31.4 | 12 | MT |
| gi\|94400790 | Heat shock protein 1 | 33.2 | 4 | PM |
| gi\|94400790 | Heat shock protein 1 | 35.1 | 4 | MT |
| gi\|81890517 | Heat shock protein 105 kDa | 14.1 | 4 | CS |
| gi\|38303969 | Heat shock protein 5 | 26.6 | 13 | CS |
| gi\|38303969 | Heat shock protein 5 | 52.8 | 29 | PM |
| gi\|38303969 | Heat shock protein 5 | 46.5 | 26 | MT |
| gi\|71051777 | Heat shock protein 8 | 56.4 | 41 | CS |
| gi\|71051777 | Heat shock protein 8 | 39.5 | 19 | PM |
| gi\|71051777 | Heat shock protein 8 | 43.7 | 20 | MT |
| gi\|54673763 | Heat shock protein 90, α (cytosolic), class A member 1 | 44.5 | 23 | CS |
| gi\|6016269 | Heat shock protein β-2 | 61.0 | 3 | CS |
| gi\|6016271 | Heat shock protein β-6 | 55.6 | 11 | CS |
| gi\|6016271 | Heat shock protein β-6 | 21.0 | 3 | PM |
| gi\|157822045 | Hedgehog acyltransferase-like | 21.1 | 10 | PM |
| gi\|6981010 | Hemoglobin α 1 chain | 85.9 | 78 | CS |
| gi\|6981010 | Hemoglobin α 1 chain | 73.9 | 30 | PM |
| gi\|6981010 | Hemoglobin α 1 chain | 70.4 | 23 | MT |
| gi\|483109 | Hemoglobin α-2 chain - rat (tentative semence) | 48.5 | 14 | CS |
| gi\|60688311 | Hemopexin | 56.1 | 26 | CS |
| gi\|60688311 | Hemopexin | 19.6 | 6 | PM |
| gi\|60688311 | Hemopexin | 9.8 | 1 | MT |

| # Accession | Protein name | % Cov | Peptides | Fraction |
|---|---|---|---|---|
| gi\|52788205 | Hepatoma-derived growth factor | 18.1 | 2 | CS |
| gi\|149052456 | Heterogeneous nuclear ribonucleoprotein H1, isoform CRA_b | 9.6 | 2 | CS |
| gi\|48429097 | Heterogeneous nuclear ribonucleoprotein K | 15.1 | 1 | CS |
| gi\|6981022 | Hexokinase 1 | 36.4 | 22 | PM |
| gi\|6981022 | Hexokinase 1 | 34.9 | 18 | MT |
| gi\|183986567 | HIG1 domain family, member 2A | 33.0 | 2 | MT |
| gi\|31077132 | Histidine rich calcium binding protein | 30.2 | 16 | MT |
| gi\|149055944 | Histidine rich calcium binding protein, isoform CRA_a | 9.6 | 3 | CS |
| gi\|164565401 | Histidine triad nucleotide binding protein 2 | 39.9 | 3 | CS |
| gi\|72679585 | Histone cluster 1, H4b | 31.1 | 3 | PM |
| gi\|72679585 | Histone cluster 1, H4b | 45.6 | 5 | MT |
| gi\|281312197 | Histone H2A type 2-A | 44.6 | 3 | MT |
| gi\|9624483 | Hormone-regulated proliferation-associated 20 kDa protein short form | 37.4 | 5 | PM |
| gi\|951425 | Housekeeping protein | 18.9 | 3 | PM |
| gi\|48734844 | Hsd17b4 protein | 4.9 | 2 | PM |
| gi\|48734844 | Hsd17b4 protein | 24.8 | 8 | MT |
| gi\|7387725 | Hydroxyacyl-CoA dehydrogenase, mitochondrial | 34.1 | 10 | PM |
| gi\|7387725 | Hydroxyacyl-CoA dehydrogenase, mitochondrial | 30.6 | 6 | MT |
| gi\|81907928 | Hydroxysteroid dehydrogenase-like protein 2 | 37.4 | 11 | CS |
| gi\|81907928 | Hydroxysteroid dehydrogenase-like protein 2 | 13.4 | 4 | PM |
| gi\|81907928 | Hydroxysteroid dehydrogenase-like protein 2 | 11.3 | 4 | MT |
| gi\|157787020 | Hypothetical protein LOC289278 | 31.6 | 3 | PM |
| gi\|157787020 | Hypothetical protein LOC289278 | 22.2 | 2 | MT |
| gi\|158262028 | Hypothetical protein LOC294231 | 9.0 | 1 | PM |
| gi\|187282120 | Hypothetical protein LOC299909 | 18.8 | 2 | CS |
| gi\|157819345 | Hypothetical protein LOC313776 | 22.8 | 7 | MT |
| gi\|157822273 | Hypothetical protein LOC315463 | 13.5 | 2 | PM |
| gi\|157822273 | Hypothetical protein LOC315463 | 18.7 | 2 | MT |
| gi\|157817209 | Hypothetical protein LOC361606 | 27.4 | 2 | CS |
| gi\|157817213 | Hypothetical protein LOC497874 | 38.2 | 2 | PM |
| gi\|157817213 | Hypothetical protein LOC497874 | 38.2 | 4 | MT |
| gi\|157817241 | Hypothetical protein LOC500694 | 25.3 | 2 | CS |
| gi\|155369672 | Hypothetical protein LOC500874 | 17.6 | 4 | PM |
| gi\|155369672 | Hypothetical protein LOC500874 | 26.8 | 3 | MT |

| # Accession | Protein name | % Cov | Peptides | Fraction |
|---|---|---|---|---|
| gi\|169234816 | Hypothetical protein LOC681996 | 12.4 | 1 | CS |
| gi\|68534712 | Hypoxanthine phosphoribosyltransferase 1 | 15.6 | 2 | PM |
| gi\|93279231 | Chain A, Acyl-CoA Oxidase Complexed With 3-OH-Dodecanoate | 10.9 | 2 | MT |
| gi\|28373861 | Chain A, Annexin V K27e Mutant | 71.2 | 18 | CS |
| gi\|9257037 | Chain A, M-Calpain | 18.6 | 4 | CS |
| gi\|93279422 | Chain A, Rat Liver F1-Atpase | 83.5 | 189 | PM |
| gi\|158428857 | Chain A, Rat Phosphatidylethanolamine-Binding Protein | 91.6 | 25 | CS |
| gi\|186973052 | Chain A, Structure Of Rattus Norvegicus Ntpdase2 In Complex With Calcium And Amppnp | 18.2 | 3 | PM |
| gi\|6435548 | Chain B, Mammalian 2-Cys Peroxiredoxin, Hbp23 | 58.3 | 13 | CS |
| gi\|1942646 | Chain B, Rat Procathepsin B | 29.2 | 2 | CS |
| gi\|3318959 | Chain B, Three-Dimensional Structure Of Nadph-Cytochrome P450 Reductase | 19.7 | 7 | PM |
| gi\|3318959 | Chain B, Three-Dimensional Structure Of Nadph-Cytochrome P450 Reductase | 16.4 | 4 | MT |
| gi\|48425083 | Chain D, Monoamine Oxidase A | 53.9 | 26 | PM |
| gi\|48425083 | Chain D, Monoamine Oxidase A | 43.5 | 23 | MT |
| gi\|93279424 | Chain G, Rat Liver F1-Atpase | 56.8 | 32 | PM |
| gi\|81882496 | Chaperone activity of bc1 complex-like, mitochondrial | 25.0 | 9 | CS |
| gi\|81882496 | Chaperone activity of bc1 complex-like, mitochondrial | 18.2 | 6 | PM |
| gi\|81882496 | Chaperone activity of bc1 complex-like, mitochondrial | 11.7 | 4 | MT |
| gi\|149059759 | Chaperonin subunit 8 (θ) (predicted), isoform CRA_a | 6.9 | 2 | CS |
| gi\|312924 | CHIP28k | 19.3 | 1 | MT |
| gi\|149024253 | Chloride intracellular channel 4, isoform CRA_b | 34.0 | 4 | CS |
| gi\|623560 | Immunoglobulin γ-2b | 15.4 | 2 | CS |
| gi\|501064 | Immunophilin FKBP12 | 40.7 | 3 | CS |
| gi\|149036390 | Inner membrane protein, mitochondrial, isoform CRA_a | 64.3 | 51 | PM |
| gi\|149036390 | Inner membrane protein, mitochondrial, isoform CRA_a | 60.1 | 57 | MT |
| gi\|157821079 | Inosine triphosphatase | 27.8 | 2 | CS |
| gi\|149027464 | Insulin-like growth factor 2 receptor, isoform CRA_d | 2.8 | 2 | PM |
| gi\|59808174 | Inter α-trypsin inhibitor, heavy chain 4 | 16.9 | 7 | CS |
| gi\|59808174 | Inter α-trypsin inhibitor, heavy chain 4 | 6.1 | 2 | MT |
| gi\|8393899 | Inter-α trypsin inhibitor, heavy chain 3 precursor | 11.8 | 7 | CS |
| gi\|68051964 | Isocitrate dehydrogenase [NAD] subunit β, mitochondrial | 24.9 | 6 | PM |
| gi\|68051964 | Isocitrate dehydrogenase [NAD] subunit β, mitochondrial | 18.7 | 5 | MT |
| gi\|6166247 | Isocitrate dehydrogenase [NAD] subunit γ, mitochondrial | 11.5 | 2 | MT |
| gi\|62079055 | Isocitrate dehydrogenase 2 (NADP+), mitochondrial precursor | 69.9 | 80 | CS |

| # Accession | Protein name | % Cov | Peptides | Fraction |
|---|---|---|---|---|
| gi\|62079055 | Isocitrate dehydrogenase 2 (NADP+), mitochondrial precursor | 71.2 | 36 | PM |
| gi\|149041699 | Isocitrate dehydrogenase 3 (NAD+) α, isoform CRA_a | 51.3 | 17 | CS |
| gi\|149041702 | Isocitrate dehydrogenase 3 (NAD+) α, isoform CRA_d | 35.6 | 7 | PM |
| gi\|149041704 | Isocitrate dehydrogenase 3 (NAD+) α, isoform CRA_f | 32.4 | 8 | MT |
| gi\|54020666 | Isocitrate dehydrogenase 3, γ precursor | 44.8 | 7 | CS |
| gi\|54020666 | Isocitrate dehydrogenase 3, γ precursor | 24.2 | 4 | PM |
| gi\|81863750 | Isochorismatase domain-containing protein 1 | 20.5 | 1 | CS |
| gi\|6981112 | Isovaleryl CoA dehydrogenase precursor | 52.4 | 21 | CS |
| gi\|6981112 | Isovaleryl CoA dehydrogenase precursor | 42.0 | 10 | PM |
| gi\|6981112 | Isovaleryl CoA dehydrogenase precursor | 30.2 | 6 | MT |
| gi\|81885083 | Junction plakoglobin | 10.3 | 2 | PM |
| gi\|81885083 | Junction plakoglobin | 15.4 | 4 | MT |
| gi\|83816931 | Junctophilin 2 | 24.1 | 7 | MT |
| gi\|8393610 | Karyopherin (importin) β 1 | 12.9 | 2 | CS |
| gi\|120474989 | Keratin 1 | 30.4 | 8 | MT |
| gi\|73920214 | Keratin, type I cytoskeletal 19 | 18.6 | 4 | MT |
| gi\|81891699 | Keratin, type II cytoskeletal 2 epidermal | 34.7 | 7 | MT |
| gi\|81891700 | Keratin, type II cytoskeletal 73 | 13.2 | 4 | MT |
| gi\|83776543 | Kinesin family member 5B | 9.5 | 2 | CS |
| gi\|80861401 | Kininogen 1 | 22.6 | 7 | CS |
| gi\|81861882 | Kynurenine--oxoglutarate transaminase 3 | 10.8 | 2 | CS |
| gi\|157820173 | L-2-hydroxyglutarate dehydrogenase | 14.3 | 3 | PM |
| gi\|157820173 | L-2-hydroxyglutarate dehydrogenase | 10.8 | 2 | MT |
| gi\|8393706 | Lactate dehydrogenase A | 32.2 | 7 | PM |
| gi\|8393706 | Lactate dehydrogenase A | 23.5 | 8 | MT |
| gi\|81885359 | Lactoylglutathione lyase | 54.9 | 4 | CS |
| gi\|149048132 | Lamin A, isoform CRA_b | 11.3 | 2 | MT |
| gi\|8393693 | Laminin receptor 1 | 30.5 | 4 | PM |
| gi\|8393693 | Laminin receptor 1 | 20.0 | 3 | MT |
| gi\|157818227 | Laminin, β 1 | 2.5 | 2 | PM |
| gi\|281371490 | Laminin, γ 1 | 6.5 | 1 | MT |
| gi\|9845261 | Lectin, galactoside-binding, soluble, 1 | 46.7 | 5 | CS |
| gi\|9845261 | Lectin, galactoside-binding, soluble, 1 | 37.0 | 6 | PM |
| gi\|9845261 | Lectin, galactoside-binding, soluble, 1 | 43.0 | 6 | MT |

| # Accession | Protein name | % Cov | Peptides | Fraction |
|---|---|---|---|---|
| gi\|169234844 | Lectin, mannose-binding 2 | 12.9 | 1 | PM |
| gi\|169234844 | Lectin, mannose-binding 2 | 11.7 | 2 | MT |
| gi\|687712 | Lens epithelial protein | 23.8 | 4 | CS |
| gi\|62510718 | LETM1 and EF-hand domain-containing protein 1, mitochondrial | 31.4 | 10 | PM |
| gi\|62510718 | LETM1 and EF-hand domain-containing protein 1, mitochondrial | 19.4 | 12 | MT |
| gi\|81871846 | Leucine-rich PPR motif-containing protein, mitochondrial | 22.6 | 13 | CS |
| gi\|149050517 | Leucine-rich PPR-motif containing, isoform CRA_b | 12.5 | 4 | PM |
| gi\|19424264 | Leucyl/cystinyl aminopeptidase isoform 2 | 7.9 | 2 | PM |
| gi\|397357 | L-iditol 2-dehydrogenase | 15.8 | 3 | CS |
| gi\|56676346 | LIM and cysteine-rich domains 1 | 16.4 | 2 | CS |
| gi\|6981168 | Lipoprotein lipase precursor | 9.7 | 2 | PM |
| gi\|6981168 | Lipoprotein lipase precursor | 13.3 | 2 | MT |
| gi\|6981146 | L-lactate dehydrogenase B | 58.4 | 55 | CS |
| gi\|6981146 | L-lactate dehydrogenase B | 41.0 | 14 | PM |
| gi\|56788960 | LOC367586 protein | 25.6 | 6 | CS |
| gi\|60688598 | LOC367586 protein | 16.8 | 3 | PM |
| gi\|68534280 | LOC500183 protein | 23.5 | 3 | CS |
| gi\|71051822 | LOC683313 protein | 22.3 | 5 | MT |
| gi\|81916424 | Lon protease homolog, mitochondrial | 6.5 | 2 | PM |
| gi\|81916424 | Lon protease homolog, mitochondrial | 5.6 | 2 | MT |
| gi\|6978431 | Long-chain acyl-CoA dehydrogenase precursor | 63.7 | 64 | CS |
| gi\|6978431 | Long-chain acyl-CoA dehydrogenase precursor | 56.3 | 33 | PM |
| gi\|5759131 | Low molecular weight protein tyrosine phosphatase isoform A | 40.5 | 2 | CS |
| gi\|149062241 | LRP16 protein | 32.8 | 7 | CS |
| gi\|37361818 | LRRGT00066 | 11.0 | 2 | PM |
| gi\|643024 | Lumican, secretory intersticial proteoglycan | 26.3 | 4 | CS |
| gi\|226694805 | LYR motif-containing protein 7 | 49.0 | 1 | CS |
| gi\|6981362 | Lysophospholipase 1 | 20.0 | 3 | CS |
| gi\|6981144 | Lysosomal-associated membrane protein 1 precursor | 15.7 | 2 | MT |
| gi\|32129697 | MACRO domain-containing protein 1 | 23.3 | 2 | PM |
| gi\|694108 | Macrophage migration inhibitory factor | 47.8 | 4 | CS |
| gi\|81884568 | Macrophage-capping protein | 16.3 | 3 | CS |
| gi\|81861572 | Malate dehydrogenase, cytoplasmic | 52.1 | 50 | CS |
| gi\|81861572 | Malate dehydrogenase, cytoplasmic | 43.7 | 12 | PM |

| # Accession | Protein name | % Cov | Peptides | Fraction |
|---|---|---|---|---|
| gi\|81861572 | Malate dehydrogenase, cytoplasmic | 38.6 | 11 | MT |
| gi\|42476181 | Malate dehydrogenase, mitochondrial precursor | 72.5 | 47 | CS |
| gi\|42476181 | Malate dehydrogenase, mitochondrial precursor | 71.6 | 29 | PM |
| gi\|42476181 | Malate dehydrogenase, mitochondrial precursor | 68.3 | 22 | MT |
| gi\|62510703 | Mannose-6-phosphate isomerase | 9.0 | 2 | CS |
| gi\|8392833 | Medium-chain acyl-CoA dehydrogenase precursor | 49.6 | 17 | CS |
| gi\|8392833 | Medium-chain acyl-CoA dehydrogenase precursor | 39.2 | 15 | PM |
| gi\|8392833 | Medium-chain acyl-CoA dehydrogenase precursor | 18.3 | 6 | MT |
| gi\|62900631 | Membrane-associated progesterone receptor component 2 | 10.6 | 2 | PM |
| gi\|55824737 | Mercaptopyruvate sulfurtransferase | 42.1 | 9 | CS |
| gi\|56605654 | Metaxin 2 | 36.9 | 6 | PM |
| gi\|56605654 | Metaxin 2 | 28.9 | 9 | MT |
| gi\|81883845 | Methylcrotonoyl-CoA carboxylase β chain, mitochondrial | 29.3 | 10 | PM |
| gi\|81883845 | Methylcrotonoyl-CoA carboxylase β chain, mitochondrial | 13.0 | 2 | MT |
| gi\|81882966 | Methylcrotonoyl-CoA carboxylase subunit α, mitochondrial | 21.7 | 9 | PM |
| gi\|81882966 | Methylcrotonoyl-CoA carboxylase subunit α, mitochondrial | 13.0 | 6 | MT |
| gi\|400269 | Methylmalonate-semialdehyde dehydrogenase [acylating], mitochondrial | 70.8 | 50 | CS |
| gi\|400269 | Methylmalonate-semialdehyde dehydrogenase [acylating], mitochondrial | 49.2 | 19 | PM |
| gi\|400269 | Methylmalonate-semialdehyde dehydrogenase [acylating], mitochondrial | 29.4 | 13 | MT |
| gi\|157821869 | Methylmalonyl CoA epimerase | 46.6 | 4 | CS |
| gi\|710563 | MHC class I protein | 18.9 | 2 | MT |
| gi\|81909845 | Microtubule-associated protein 4 | 8.4 | 3 | CS |
| gi\|2780408 | MIPP65 | 33.2 | 15 | PM |
| gi\|47605758 | Mitofusin-1 | 10.9 | 4 | PM |
| gi\|47605758 | Mitofusin-1 | 8.9 | 2 | MT |
| gi\|166851834 | Mitochondria-associated granulocyte macrophage CSF signaling molecule | 40.8 | 3 | PM |
| gi\|166851834 | Mitochondria-associated granulocyte macrophage CSF signaling molecule | 32.0 | 3 | MT |
| gi\|81170680 | Mitochondrial antiviral-signaling protein | 26.8 | 3 | MT |
| gi\|165971637 | Mitochondrial carrier homolog 2 | 41.3 | 10 | MT |
| gi\|165971637 | Mitochondrial carrier homolog 2 | 28.4 | 7 | PM |
| gi\|94711371 | Mitochondrial fission 1 protein | 44.1 | 4 | PM |
| gi\|94711371 | Mitochondrial fission 1 protein | 59.2 | 6 | MT |
| gi\|59800393 | Mitochondrial import inner membrane translocase subunit Tim13 | 41.1 | 2 | CS |
| gi\|59800393 | Mitochondrial import inner membrane translocase subunit Tim13 | 57.9 | 3 | MT |

| # Accession | Protein name | % Cov | Peptides | Fraction |
|---|---|---|---|---|
| gi\|90110082 | Mitochondrial import inner membrane translocase subunit Tim9 | 47.2 | 3 | PM |
| gi\|90110082 | Mitochondrial import inner membrane translocase subunit Tim9 | 33.7 | 2 | MT |
| gi\|81864913 | Mitochondrial import receptor subunit TOM22 homolog | 56.3 | 6 | PM |
| gi\|81864912 | Mitochondrial import receptor subunit TOM40 homolog | 19.4 | 4 | MT |
| gi\|81911805 | Mitochondrial import receptor subunit TOM70 | 21.8 | 3 | PM |
| gi\|81911805 | Mitochondrial import receptor subunit TOM70 | 17.5 | 4 | MT |
| gi\|206597496 | Mitochondrial isoleucine tRNA synthetase | 17.1 | 2 | CS |
| gi\|206597496 | Mitochondrial isoleucine tRNA synthetase | 8.9 | 2 | PM |
| gi\|510110 | Mitochondrial long-chain 3-ketoacyl-CoA thiolase β subunit | 20.4 | 4 | CS |
| gi\|510110 | Mitochondrial long-chain 3-ketoacyl-CoA thiolase β subunit | 64.2 | 36 | PM |
| gi\|510110 | Mitochondrial long-chain 3-ketoacyl-CoA thiolase β subunit | 66.3 | 34 | MT |
| gi\|239049264 | Mitochondrial malic enzyme 3 | 27.6 | 12 | CS |
| gi\|239049264 | Mitochondrial malic enzyme 3 | 9.6 | 3 | PM |
| gi\|56553296 | Mitochondrial OPA1 | 67.6 | 2 | MT |
| gi\|599963 | Mitochondrial oxidative phosphorylation coupling factor 6 | 62.0 | 31 | PM |
| gi\|599963 | Mitochondrial oxidative phosphorylation coupling factor 6 | 62.0 | 32 | MT |
| gi\|55741522 | Mitochondrial protein 18 kDa | 21.1 | 3 | PM |
| gi\|55741522 | Mitochondrial protein 18 kDa | 19.9 | 2 | MT |
| gi\|71361655 | Mitochondrial ribosomal protein L12 | 12.0 | 2 | PM |
| gi\|157820131 | Mitochondrial ribosomal protein S35 | 10.6 | 2 | PM |
| gi\|149042266 | Moesin, isoform CRA_a | 35.4 | 10 | PM |
| gi\|32363196 | Moesin | 10.2 | 2 | MT |
| gi\|81882200 | MOSC domain-containing protein 2, mitochondrial | 13.3 | 1 | MT |
| gi\|68534595 | Mtx1 protein | 11.5 | 3 | PM |
| gi\|68534595 | Mtx1 protein | 8.1 | 1 | MT |
| gi\|81870614 | Murinoglobulin-1 | 42.2 | 52 | CS |
| gi\|81872292 | Murinoglobulin-2 | 7.8 | 6 | PM |
| gi\|6978661 | Muscle creatine kinase | 84.3 | 83 | CS |
| gi\|6978661 | Muscle creatine kinase | 70.9 | 23 | PM |
| gi\|6978661 | Muscle creatine kinase | 57.5 | 19 | MT |
| gi\|158138498 | Muscle glycogen phosphorylase | 71.1 | 82 | CS |
| gi\|535069 | Muscle LIM protein | 48.5 | 5 | CS |
| gi\|535069 | Muscle LIM protein | 19.1 | 2 | PM |
| gi\|81870810 | Myeloid-associated differentiation marker | 9.4 | 2 | PM |

| # Accession | Protein name | % Cov | Peptides | Fraction |
|---|---|---|---|---|
| gi\|197927186 | Myofibrillogenesis regulator 1 isoform 3 | 35.2 | 3 | PM |
| gi\|197927186 | Myofibrillogenesis regulator 1 isoform 3 | 43.0 | 3 | MT |
| gi\|78099013 | Myoglobin | 94.2 | 103 | CS |
| gi\|78099013 | Myoglobin | 89.6 | 24 | PM |
| gi\|78099013 | Myoglobin | 81.8 | 19 | MT |
| gi\|281306803 | Myomesin 2 | 18.4 | 10 | CS |
| gi\|157824043 | Myosin binding protein C, cardiac | 4.5 | 2 | CS |
| gi\|400429 | Myosin I heavy chain | 16.6 | 7 | PM |
| gi\|160961485 | Myosin light chain kinase 3 | 30.7 | 6 | CS |
| gi\|149066032 | Myosin, heavy polypeptide 9, non-muscle | 11.5 | 2 | CS |
| gi\|149066032 | Myosin, heavy polypeptide 9, non-muscle | 9.1 | 8 | PM |
| gi\|149066032 | Myosin, heavy polypeptide 9, non-muscle | 12.2 | 8 | MT |
| gi\|266495 | Myristoylated alanine-rich C-kinase substrate | 25.9 | 3 | MT |
| gi\|6831527 | N(G),N(G)-dimethylarginine dimethylaminohydrolase 1 | 22.8 | 5 | CS |
| gi\|6978543 | Na+/K+ -ATPase α 1 subunit precursor | 49.1 | 53 | PM |
| gi\|6978543 | Na+/K+ -ATPase α 1 subunit precursor | 28.3 | 25 | MT |
| gi\|6978545 | Na+/K+ -ATPase α 2 subunit precursor | 28.5 | 28 | PM |
| gi\|6978545 | Na+/K+ -ATPase α 2 subunit precursor | 18.5 | 11 | MT |
| gi\|81884377 | NAD-dependent deacetylase sirtuin-5 | 22.9 | 2 | CS |
| gi\|6981260 | NADH dehydrogenase (ubiquinone) 1 α subcomplex 5 | 91.4 | 10 | PM |
| gi\|6981260 | NADH dehydrogenase (ubiquinone) 1 α subcomplex 5 | 90.5 | 8 | MT |
| gi\|157818537 | NADH dehydrogenase (ubiquinone) 1 α subcomplex, 1 | 23.3 | 1 | PM |
| gi\|164565371 | NADH dehydrogenase (ubiquinone) 1 α subcomplex, 12 | 82.8 | 15 | PM |
| gi\|164565371 | NADH dehydrogenase (ubiquinone) 1 α subcomplex, 12 | 79.3 | 12 | MT |
| gi\|157817861 | NADH dehydrogenase (ubiquinone) 1 α subcomplex, 2 | 67.0 | 7 | PM |
| gi\|157817861 | NADH dehydrogenase (ubiquinone) 1 α subcomplex, 2 | 76.3 | 7 | MT |
| gi\|189085365 | NADH dehydrogenase (ubiquinone) 1 α subcomplex, 4 | 64.6 | 31 | PM |
| gi\|189085365 | NADH dehydrogenase (ubiquinone) 1 α subcomplex, 4 | 63.4 | 25 | MT |
| gi\|194473636 | NADH dehydrogenase (ubiquinone) 1 α subcomplex, 6 | 73.1 | 15 | PM |
| gi\|194473636 | NADH dehydrogenase (ubiquinone) 1 α subcomplex, 6 | 70.8 | 15 | MT |
| gi\|149038913 | NADH dehydrogenase (ubiquinone) 1 α subcomplex, 8 | 82.0 | 13 | PM |
| gi\|149038913 | NADH dehydrogenase (ubiquinone) 1 α subcomplex, 8 | 82.0 | 15 | MT |
| gi\|157824071 | NADH dehydrogenase (ubiquinone) 1 β subcomplex 3 | 40.4 | 4 | PM |
| gi\|157824071 | NADH dehydrogenase (ubiquinone) 1 β subcomplex 3 | 40.4 | 3 | MT |

| # Accession | Protein name | % Cov | Peptides | Fraction |
|---|---|---|---|---|
| gi\|82617686 | NADH dehydrogenase (ubiquinone) 1 β subcomplex 4 | 64.3 | 17 | PM |
| gi\|82617686 | NADH dehydrogenase (ubiquinone) 1 β subcomplex 4 | 72.1 | 15 | MT |
| gi\|187469737 | NADH dehydrogenase (ubiquinone) 1 β subcomplex 8 | 57.0 | 19 | PM |
| gi\|187469737 | NADH dehydrogenase (ubiquinone) 1 β subcomplex 8 | 63.4 | 17 | MT |
| gi\|157822175 | NADH dehydrogenase (ubiquinone) 1 β subcomplex, 10 | 80.7 | 28 | PM |
| gi\|157822175 | NADH dehydrogenase (ubiquinone) 1 β subcomplex, 10 | 83.0 | 29 | MT |
| gi\|157822851 | NADH dehydrogenase (ubiquinone) 1 β subcomplex, 11 | 53.6 | 11 | PM |
| gi\|157822851 | NADH dehydrogenase (ubiquinone) 1 β subcomplex, 11 | 71.5 | 15 | MT |
| gi\|219277692 | NADH dehydrogenase (ubiquinone) 1 β subcomplex, 2 | 22.9 | 2 | PM |
| gi\|219277692 | NADH dehydrogenase (ubiquinone) 1 β subcomplex, 2 | 22.9 | 2 | MT |
| gi\|157823387 | NADH dehydrogenase (ubiquinone) 1 β subcomplex, 5 | 33.9 | 9 | PM |
| gi\|157823387 | NADH dehydrogenase (ubiquinone) 1 β subcomplex, 5 | 36.5 | 8 | MT |
| gi\|157820465 | NADH dehydrogenase (ubiquinone) 1 β subcomplex, 6 | 54.7 | 4 | PM |
| gi\|157820465 | NADH dehydrogenase (ubiquinone) 1 β subcomplex, 6 | 59.4 | 6 | MT |
| gi\|157823197 | NADH dehydrogenase (ubiquinone) 1 β subcomplex, 7 | 74.5 | 17 | PM |
| gi\|157823197 | NADH dehydrogenase (ubiquinone) 1 β subcomplex, 7 | 74.5 | 15 | MT |
| gi\|197245756 | NADH dehydrogenase (ubiquinone) 1 β subcomplex, 9 | 73.7 | 23 | PM |
| gi\|197245756 | NADH dehydrogenase (ubiquinone) 1 β subcomplex, 9 | 59.8 | 23 | MT |
| gi\|157820787 | NADH dehydrogenase (ubiquinone) 1, α / β subcomplex, 1 | 36.5 | 12 | PM |
| gi\|57164133 | NADH dehydrogenase (ubiquinone) 1, subcomplex unknown, 2 | 50.8 | 13 | PM |
| gi\|57164133 | NADH dehydrogenase (ubiquinone) 1, subcomplex unknown, 2 | 55.0 | 11 | MT |
| gi\|157817227 | NADH dehydrogenase (ubiquinone) Fe-S protein 3 | 73.5 | 24 | MT |
| gi\|149022594 | NADH dehydrogenase (ubiquinone) Fe-S protein 3 (predicted), isoform CRA_b | 65.6 | 23 | PM |
| gi\|72086149 | NADH dehydrogenase (ubiquinone) Fe-S protein 5b | 73.6 | 13 | PM |
| gi\|72086149 | NADH dehydrogenase (ubiquinone) Fe-S protein 5b | 72.6 | 14 | MT |
| gi\|56606108 | NADH dehydrogenase (ubiquinone) Fe-S protein 7 | 48.2 | 17 | PM |
| gi\|56606108 | NADH dehydrogenase (ubiquinone) Fe-S protein 7 | 50.0 | 20 | MT |
| gi\|81882328 | NADH dehydrogenase [ubiquinone] 1 α subcomplex subunit 10, mitochondrial | 84.5 | 43 | PM |
| gi\|52000746 | NADH dehydrogenase [ubiquinone] 1 α subcomplex subunit 11 | 36.2 | 7 | PM |
| gi\|52000746 | NADH dehydrogenase [ubiquinone] 1 α subcomplex subunit 11 | 36.2 | 8 | MT |
| gi\|81882598 | NADH dehydrogenase [ubiquinone] 1 α subcomplex subunit 9, mitochondrial | 74.9 | 38 | PM |
| gi\|83305118 | NADH dehydrogenase [ubiquinone] flavoprotein 2, mitochondrial | 68.6 | 27 | PM |
| gi\|83305118 | NADH dehydrogenase [ubiquinone] flavoprotein 2, mitochondrial | 80.7 | 26 | MT |
| gi\|81890431 | NADH dehydrogenase [ubiquinone] iron-sulfur protein 2, mitochondrial | 77.8 | 43 | PM |

| # Accession | Protein name | % Cov | Peptides | Fraction |
|---|---|---|---|---|
| gi\|81890431 | NADH dehydrogenase [ubiquinone] iron-sulfur protein 2, mitochondrial | 73.4 | 39 | MT |
| gi\|81889861 | NADH dehydrogenase [ubiquinone] iron-sulfur protein 4, mitochondrial | 65.7 | 20 | PM |
| gi\|81889861 | NADH dehydrogenase [ubiquinone] iron-sulfur protein 4, mitochondrial | 44.6 | 21 | MT |
| gi\|228015040 | NADH dehydrogenase subunit 4 | 8.9 | 4 | PM |
| gi\|55741424 | NADH dehydrogenase ubiquinone flavoprotein 1 precursor | 25.0 | 4 | CS |
| gi\|55741424 | NADH dehydrogenase ubiquinone flavoprotein 1 precursor | 68.1 | 46 | PM |
| gi\|55741424 | NADH dehydrogenase ubiquinone flavoprotein 1 precursor | 76.1 | 42 | MT |
| gi\|81882716 | NADH-cytochrome b5 reductase 1 | 24.3 | 2 | PM |
| gi\|81882716 | NADH-cytochrome b5 reductase 1 | 10.8 | 2 | MT |
| gi\|81884209 | NADH-ubiquinone oxidoreductase 75 kDa subunit, mitochondrial | 72.6 | 64 | PM |
| gi\|81884209 | NADH-ubiquinone oxidoreductase 75 kDa subunit, mitochondrial | 72.4 | 75 | MT |
| gi\|163916626 | Ndufa3 protein | 49.4 | 3 | PM |
| gi\|163916626 | Ndufa3 protein | 49.4 | 2 | MT |
| gi\|163915993 | Ndufa7 protein | 75.0 | 11 | PM |
| gi\|163915993 | Ndufa7 protein | 75.9 | 12 | MT |
| gi\|165971299 | Ndufs8 protein | 55.2 | 14 | PM |
| gi\|165971299 | Ndufs8 protein | 61.8 | 10 | MT |
| gi\|212288176 | Neutral cholesterol ester hydrolase 1 | 10.3 | 3 | PM |
| gi\|212288176 | Neutral cholesterol ester hydrolase 1 | 19.1 | 4 | MT |
| gi\|40538874 | Nexilin (F actin binding protein) isoform b | 8.9 | 2 | MT |
| gi\|76880449 | NFS1 nitrogen fixation 1 homolog | 9.8 | 2 | CS |
| gi\|61557127 | Nicotinamide nucleotide transhydrogenase | 48.4 | 76 | PM |
| gi\|68565643 | Nicotinamide phosphoribosyltransferase | 23.6 | 3 | CS |
| gi\|77628000 | Nitrilase family, member 2 | 59.1 | 11 | CS |
| gi\|81888874 | NLR family member X1 | 13.3 | 6 | PM |
| gi\|81888874 | NLR family member X1 | 20.5 | 5 | MT |
| gi\|6822247 | Nogo-A protein | 7.7 | 5 | PM |
| gi\|7839520 | NPW16 | 60.7 | 6 | PM |
| gi\|48734832 | NSFL1 (p97) cofactor (p47) | 28.1 | 4 | MT |
| gi\|9910324 | Nuclear protein E3-3 isoform a | 21.1 | 3 | MT |
| gi\|55926145 | Nucleoside diphosphate kinase B | 88.2 | 21 | CS |
| gi\|55926145 | Nucleoside diphosphate kinase B | 63.2 | 8 | PM |
| gi\|55926145 | Nucleoside diphosphate kinase B | 50.0 | 4 | MT |
| gi\|81883766 | OCIA domain-containing protein 1 | 52.2 | 5 | PM |

| # Accession | Protein name | % Cov | Peptides | Fraction |
|---|---|---|---|---|
| gi\|81883766 | OCIA domain-containing protein 1 | 50.2 | 5 | MT |
| gi\|83405907 | Optic atrophy 1 homolog | 30.1 | 22 | PM |
| gi\|83405907 | Optic atrophy 1 homolog | 34.9 | 32 | MT |
| gi\|38181818 | Ornithine aminotransferase | 27.8 | 5 | CS |
| gi\|5106930 | Outer membrane protein | 49.0 | 6 | MT |
| gi\|274318365 | Oxidase assembly 1-like | 11.6 | 4 | PM |
| gi\|81912692 | Paraplegin | 11.3 | 3 | PM |
| gi\|81912692 | Paraplegin | 12.2 | 3 | MT |
| gi\|66911068 | Pcbp2 protein | 14.9 | 2 | CS |
| gi\|50925459 | Pcyt2 protein | 17.1 | 2 | CS |
| gi\|60688224 | Pdhx protein | 24.7 | 4 | CS |
| gi\|8393153 | PDZ and LIM domain 1 | 11.3 | 2 | CS |
| gi\|56268806 | Peptidylprolyl isomerase F (cyclophilin F) | 56.8 | 7 | CS |
| gi\|56268806 | Peptidylprolyl isomerase F (cyclophilin F) | 28.2 | 3 | PM |
| gi\|56268806 | Peptidylprolyl isomerase F (cyclophilin F) | 24.3 | 2 | MT |
| gi\|34849738 | Peroxiredoxin 2 | 62.1 | 12 | CS |
| gi\|149040547 | Peroxiredoxin 3 | 44.8 | 15 | PM |
| gi\|149040547 | Peroxiredoxin 3 | 21.4 | 5 | MT |
| gi\|51261175 | Peroxiredoxin 5 | 62.0 | 10 | CS |
| gi\|5902791 | Peroxiredoxin-6 | 67.4 | 22 | CS |
| gi\|5902791 | Peroxiredoxin-6 | 25.0 | 4 | PM |
| gi\|5902791 | Peroxiredoxin-6 | 22.8 | 4 | MT |
| gi\|6491860 | Peroxisomal 2,4-dienoyl CoA reductase | 23.1 | 4 | PM |
| gi\|81883743 | Peroxisomal 3,2-trans-enoyl-CoA isomerase | 17.7 | 5 | CS |
| gi\|81883743 | Peroxisomal 3,2-trans-enoyl-CoA isomerase | 11.5 | 4 | PM |
| gi\|81883743 | Peroxisomal 3,2-trans-enoyl-CoA isomerase | 9.7 | 1 | MT |
| gi\|76781470 | Peroxisomal short-chain alcohol dehydrogenase | 15.1 | 2 | PM |
| gi\|76781470 | Peroxisomal short-chain alcohol dehydrogenase | 13.3 | 3 | MT |
| gi\|41350889 | Pgam1 protein | 66.5 | 22 | CS |
| gi\|41350889 | Pgam1 protein | 18.1 | 3 | PM |
| gi\|118764083 | Pgm1 protein | 49.9 | 22 | CS |
| gi\|67460102 | Phosphatidate cytidylyltransferase 2 | 10.4 | 3 | PM |
| gi\|67460102 | Phosphatidate cytidylyltransferase 2 | 10.4 | 3 | MT |
| gi\|8393910 | Phosphatidylethanolamine binding protein | 40.1 | 4 | PM |

| # Accession | Protein name | % Cov | Peptides | Fraction |
|---|---|---|---|---|
| gi\|8393910 | Phosphatidylethanolamine binding protein | 20.9 | 3 | MT |
| gi\|62825891 | Phosphofructokinase, muscle | 26.2 | 7 | CS |
| gi\|56585024 | Phosphoglycerate kinase 1 | 77.2 | 42 | CS |
| gi\|56585024 | Phosphoglycerate kinase 1 | 30.0 | 7 | PM |
| gi\|56585024 | Phosphoglycerate kinase 1 | 25.7 | 8 | MT |
| gi\|8393948 | Phosphoglycerate mutase 2 | 51.0 | 29 | CS |
| gi\|8393948 | Phosphoglycerate mutase 2 | 35.2 | 7 | PM |
| gi\|164663846 | Phosphohistidine phosphatase 1 | 58.9 | 6 | CS |
| gi\|22654268 | Phospholemman | 38.0 | 3 | PM |
| gi\|158341684 | Phospholipase A2, activating protein | 10.1 | 2 | CS |
| gi\|197246445 | Phosphomannomutase 2 | 26.0 | 6 | CS |
| gi\|171846774 | Phosphorylase, glycogen, muscle | 13.1 | 6 | PM |
| gi\|157819139 | Pitrilysin metallopeptidase 1 | 7.0 | 2 | CS |
| gi\|60688649 | Plasminogen | 9.0 | 4 | PM |
| gi\|60688649 | Plasminogen | 10.8 | 3 | MT |
| gi\|51702760 | Platelet-activating factor acetylhydrolase IB subunit α | 14.4 | 3 | CS |
| gi\|157786694 | Polymerase I and transcript release factor | 53.3 | 21 | PM |
| gi\|157786694 | Polymerase I and transcript release factor | 16.6 | 3 | CS |
| gi\|71122474 | Ppa1 protein | 22.7 | 3 | CS |
| gi\|38541053 | Ppib protein | 16.7 | 2 | PM |
| gi\|57012987 | PRA1 family protein 3 | 19.7 | 2 | PM |
| gi\|158706096 | Pre-B-cell leukemia transcription factor-interacting protein 1 | 16.5 | 6 | PM |
| gi\|158706096 | Pre-B-cell leukemia transcription factor-interacting protein 1 | 15.1 | 5 | MT |
| gi\|55855 | Precursor (AA -17 to 399) | 51.2 | 10 | PM |
| gi\|55855 | Precursor (AA -17 to 399) | 19.7 | 5 | MT |
| gi\|56072 | Precursor polypeptide (AA -29 to 261) | 48.6 | 21 | CS |
| gi\|56072 | Precursor polypeptide (AA -29 to 261) | 53.1 | 12 | PM |
| gi\|56164 | Precursor polypeptide (AA -32 to 2445) | 2.0 | 1 | PM |
| gi\|56164 | Precursor polypeptide (AA -32 to 2445) | 3.1 | 3 | MT |
| gi\|109511985 | PREDICTED: hypothetical protein | 29.8 | 6 | PM |
| gi\|109511985 | PREDICTED: hypothetical protein | 29.5 | 7 | MT |
| gi\|62655115 | PREDICTED: similar to 40S ribosomal protein S2 | 20.3 | 3 | MT |
| gi\|27658914 | PREDICTED: similar to 60S ribosomal protein L21 | 22.6 | 1 | PM |
| gi\|62638415 | PREDICTED: similar to 60S ribosomal protein L26 (SIG-20) | 22.8 | 4 | PM |

| # Accession | Protein name | % Cov | Peptides | Fraction |
|---|---|---|---|---|
| gi\|62638415 | PREDICTED: similar to 60S ribosomal protein L26 (SIG-20) | 22.8 | 4 | MT |
| gi\|62639430 | PREDICTED: similar to 60S ribosomal protein L27a | 33.1 | 2 | PM |
| gi\|109490334 | PREDICTED: similar to 60S ribosomal protein L3-like | 13.0 | 4 | PM |
| gi\|109490334 | PREDICTED: similar to 60S ribosomal protein L3-like | 24.3 | 6 | MT |
| gi\|62642554 | PREDICTED: similar to 60S ribosomal protein L7a | 13.9 | 3 | PM |
| gi\|62642554 | PREDICTED: similar to 60S ribosomal protein L7a | 22.6 | 4 | MT |
| gi\|109463553 | PREDICTED: similar to AHNAK nucleoprotein isoform 1 isoform 3 | 20.7 | 6 | CS |
| gi\|109463555 | PREDICTED: similar to AHNAK nucleoprotein isoform 1 isoform 4 | 20.1 | 5 | PM |
| gi\|62664437 | PREDICTED: similar to Aldehyde dehydrogenase family 7, member A1 | 25.2 | 6 | CS |
| gi\|62648070 | PREDICTED: similar to Aldose reductase | 11.4 | 2 | PM |
| gi\|109468300 | PREDICTED: similar to α-enolase (Enolase 1) | 63.4 | 35 | CS |
| gi\|109496584 | PREDICTED: similar to ATP synthase F0, H+ transporting, subunit f, isoform 2 | 29.7 | 8 | PM |
| gi\|109496584 | PREDICTED: similar to ATP synthase F0, H+ transporting, subunit f, isoform 2 | 29.7 | 9 | MT |
| gi\|62646841 | PREDICTED: similar to Calcium-binding mitochondrial carrier protein Aralar2 | 43.6 | 29 | PM |
| gi\|62646841 | PREDICTED: similar to Calcium-binding mitochondrial carrier protein Aralar2 | 48.2 | 25 | MT |
| gi\|109512256 | PREDICTED: similar to Cation-dependent mannose-6-phosphate receptor | 17.0 | 2 | PM |
| gi\|109497479 | PREDICTED: similar to CG2453-PA | 25.1 | 8 | PM |
| gi\|109461675 | PREDICTED: similar to Cytochrome c oxidase, subunit VIb polypeptide 1 | 66.7 | 32 | MT |
| gi\|109511686 | PREDICTED: similar to Cytochrome c-type heme lyase | 35.8 | 9 | PM |
| gi\|109511686 | PREDICTED: similar to Cytochrome c-type heme lyase | 36.1 | 6 | MT |
| gi\|109484674 | PREDICTED: similar to Dihydrolipoamide S-acetyltransferase | 37.1 | 11 | MT |
| gi\|62657092 | PREDICTED: similar to Dual specificity protein phosphatase 3 (T-DSP11) | 24.0 | 1 | CS |
| gi\|109511865 | PREDICTED: similar to Dystrophin, muscular dystrophy | 6.3 | 2 | PM |
| gi\|109501906 | PREDICTED: similar to F11C1.5a | 8.1 | 5 | CS |
| gi\|109501906 | PREDICTED: similar to F11C1.5a | 6.6 | 1 | PM |
| gi\|62646949 | PREDICTED: similar to Filamin-C | 10.9 | 8 | CS |
| gi\|62646949 | PREDICTED: similar to Filamin-C | 5.4 | 3 | MT |
| gi\|62653546 | PREDICTED: similar to Glyceraldehyde-3-phosphate dehydrogenase | 79.6 | 73 | CS |
| gi\|109485458 | PREDICTED: similar to Glycerol-3-phosphate dehydrogenase 1-like | 32.5 | 5 | CS |
| gi\|109476173 | PREDICTED: similar to High mobility group protein 1 | 31.2 | 7 | CS |
| gi\|109490737 | PREDICTED: similar to Histidine triad nucleotide-binding protein 1 | 55.4 | 4 | CS |
| gi\|27693406 | PREDICTED: similar to Histone H2B 291B isoform 2 | 42.9 | 5 | PM |
| gi\|109501541 | PREDICTED: similar to Kinectin 1 | 7.7 | 2 | MT |
| gi\|109460394 | PREDICTED: similar to Laminin α-2 chain precursor | 4.9 | 3 | PM |

| # Accession | Protein name | % Cov | Peptides | Fraction |
|---|---|---|---|---|
| gi\|62643486 | PREDICTED: similar to Large subunit ribosomal protein L36a | 17.0 | 3 | PM |
| gi\|62654757 | PREDICTED: similar to Methylmalonyl-CoA mutase, mitochondrial precursor | 18.9 | 4 | CS |
| gi\|109498993 | PREDICTED: similar to Microsomal glutathione S-transferase 3 | 35.3 | 6 | MT |
| gi\|109466625 | PREDICTED: similar to NADH dehydrogenase (ubiquinone) 1, subcomplex unknown, 1 | 12.8 | 1 | PM |
| gi\|109477385 | PREDICTED: similar to Protein 4.1 (Band 4.1) (P4.1) (4.1R) | 8.0 | 2 | PM |
| gi\|62651145 | PREDICTED: similar to Protein C14orf159, mitochondrial precursor | 15.9 | 2 | CS |
| gi\|109502869 | PREDICTED: similar to RAN binding protein 5 | 9.4 | 3 | CS |
| gi\|27664676 | PREDICTED: similar to Ribosomal protein L10 | 15.4 | 2 | MT |
| gi\|62654783 | PREDICTED: similar to Ribosomal protein L10a | 18.0 | 4 | PM |
| gi\|62658447 | PREDICTED: similar to Ribosomal protein L15 | 18.6 | 2 | PM |
| gi\|109511626 | PREDICTED: similar to Ribosomal protein L21 | 21.1 | 2 | MT |
| gi\|27680589 | PREDICTED: similar to Ribosomal protein L31 | 31.5 | 2 | MT |
| gi\|62655953 | PREDICTED: similar to Ribosomal protein S10 | 42.4 | 4 | PM |
| gi\|62660444 | PREDICTED: similar to Ribosomal protein S23 | 16.1 | 2 | MT |
| gi\|109482126 | PREDICTED: similar to Ribosomal protein S24 | 20.7 | 2 | PM |
| gi\|109482126 | PREDICTED: similar to Ribosomal protein S24 | 25.2 | 1 | MT |
| gi\|109470913 | PREDICTED: similar to Ribosome-binding protein 1 | 19.3 | 3 | MT |
| gi\|34867677 | PREDICTED: similar to Serine protease inhibitor A3M precursor | 26.7 | 9 | CS |
| gi\|34854800 | PREDICTED: similar to Solute carrier family 25, member 12 | 33.5 | 8 | PM |
| gi\|109495987 | PREDICTED: similar to Tescalcin | 34.1 | 3 | CS |
| gi\|109470142 | PREDICTED: similar to Titin isoform N2-B | 25.4 | 405 | MT |
| gi\|109483017 | PREDICTED: similar to Tubulin cofactor a (predicted) | 25.7 | 2 | CS |
| gi\|55882 | Preprocathepsin D | 7.4 | 2 | PM |
| gi\|55882 | Preprocathepsin D | 11.3 | 3 | MT |
| gi\|77748257 | Presenilin associated, rhomboid-like | 6.6 | 1 | MT |
| gi\|9653968 | Prion protein | 16.9 | 2 | PM |
| gi\|5305687 | Pro-α-2(I) collagen | 23.9 | 8 | MT |
| gi\|73919297 | Probable saccharopine dehydrogenase | 25.9 | 3 | PM |
| gi\|73919297 | Probable saccharopine dehydrogenase | 12.4 | 3 | MT |
| gi\|149043683 | Procollagen, type VI, α 1 (predicted), isoform CRA_b | 14.2 | 4 | PM |
| gi\|149043683 | Procollagen, type VI, α 1 (predicted), isoform CRA_b | 11.8 | 5 | MT |
| gi\|149037631 | Procollagen, type VI, α 3 (predicted), isoform CRA_c | 15.0 | 15 | MT |
| gi\|149045856 | Procollagen, type XV, isoform CRA_b | 5.5 | 4 | PM |
| gi\|149045856 | Procollagen, type XV, isoform CRA_b | 10.7 | 3 | MT |

| # Accession | Protein name | % Cov | Peptides | Fraction |
|---|---|---|---|---|
| gi\|51702769 | Profilin-1 | 65.7 | 6 | CS |
| gi\|66911717 | Prohibitin | 76.5 | 17 | PM |
| gi\|66911717 | Prohibitin | 76.5 | 19 | MT |
| gi\|76363296 | Prohibitin-2 | 75.9 | 26 | PM |
| gi\|76363296 | Prohibitin-2 | 74.3 | 23 | MT |
| gi\|6981324 | Prolyl 4-hydroxylase, β polypeptide | 28.4 | 9 | CS |
| gi\|6981324 | Prolyl 4-hydroxylase, β polypeptide | 17.3 | 5 | PM |
| gi\|6981324 | Prolyl 4-hydroxylase, β polypeptide | 14.2 | 3 | MT |
| gi\|51260066 | Propionyl CoA carboxylase, β polypeptide | 44.0 | 11 | CS |
| gi\|51260066 | Propionyl CoA carboxylase, β polypeptide | 36.8 | 10 | PM |
| gi\|158303308 | Propionyl-coenzyme A carboxylase, α polypeptide | 28.8 | 12 | CS |
| gi\|157822395 | Prostaglandin E synthase 2 | 48.7 | 9 | PM |
| gi\|157822395 | Prostaglandin E synthase 2 | 39.1 | 9 | MT |
| gi\|38197361 | Prostaglandin I2 (prostacyclin) synthase | 7.0 | 2 | MT |
| gi\|206558239 | Prostaglandin reductase 2 | 19.9 | 4 | CS |
| gi\|61098214 | Protease (prosome, macropain) 28 subunit, α | 17.3 | 4 | CS |
| gi\|6981420 | Protease, serine, 2 precursor | 17.9 | 20 | PM |
| gi\|6981420 | Protease, serine, 2 precursor | 19.9 | 18 | MT |
| gi\|8394091 | Proteasome activator subunit 2 | 21.0 | 1 | CS |
| gi\|81884895 | Protein BAT5 | 7.9 | 2 | PM |
| gi\|62296810 | Protein disulfide-isomerase A6 | 23.4 | 6 | PM |
| gi\|62296810 | Protein disulfide-isomerase A6 | 21.1 | 6 | MT |
| gi\|56404680 | Protein DJ-1 | 57.7 | 7 | CS |
| gi\|29611707 | Protein ERGIC-53 | 25.7 | 8 | PM |
| gi\|76880465 | Protein kinase C and casein kinase substrate in neurons 2 | 6.4 | 2 | PM |
| gi\|71681475 | Protein kinase C and casein kinase substrate in neurons 3 | 16.8 | 5 | PM |
| gi\|81870080 | Protein kinase C δ-binding protein | 9.9 | 2 | PM |
| gi\|171846648 | Protein kinase C substrate 80K-H | 8.2 | 2 | PM |
| gi\|171846648 | Protein kinase C substrate 80K-H | 8.8 | 3 | MT |
| gi\|6981396 | Protein kinase, cAMP dependent regulatory, type I, α | 28.1 | 6 | CS |
| gi\|81867103 | Protein NDRG2 | 29.1 | 5 | CS |
| gi\|81867103 | Protein NDRG2 | 49.6 | 13 | PM |
| gi\|81867103 | Protein NDRG2 | 38.5 | 5 | MT |
| gi\|6981388 | Protein phosphatase 1, catalytic subunit, β | 10.7 | 3 | MT |

| # Accession | Protein name | % Cov | Peptides | Fraction |
|---|---|---|---|---|
| gi\|8394021 | Protein phosphatase 2a, catalytic subunit, β isoform | 34.3 | 3 | CS |
| gi\|663080 | Protein phosphatase T (PPT) | 12.6 | 1 | CS |
| gi\|155369684 | Protein tyrosine phosphatase-like, member a | 5.2 | 2 | PM |
| gi\|56961640 | Protein-L-isoaspartate (D-aspartate) O-methyltransferase 1 | 40.5 | 5 | CS |
| gi\|73621422 | Protein-tyrosine phosphatase mitochondrial 1 | 16.1 | 1 | PM |
| gi\|4336877 | PRx III | 23.4 | 7 | PM |
| gi\|205829287 | Purine nucleoside phosphorylase | 30.1 | 5 | CS |
| gi\|81884356 | Pyridine nucleotide-disulfide oxidoreductase domain-containing protein 2 | 8.8 | 4 | PM |
| gi\|81884356 | Pyridine nucleotide-disulfide oxidoreductase domain-containing protein 2 | 4.6 | 2 | MT |
| gi\|56268822 | Pyridoxine 5'-phosphate oxidase | 15.7 | 2 | CS |
| gi\|209529636 | Pyrophosphatase (inorganic) 2 | 39.1 | 10 | CS |
| gi\|209529636 | Pyrophosphatase (inorganic) 2 | 8.5 | 2 | PM |
| gi\|71051030 | Pyruvate dehydrogenase (lipoamide) α 1 | 39.2 | 10 | MT |
| gi\|56090293 | Pyruvate dehydrogenase (lipoamide) β precursor | 61.0 | 26 | PM |
| gi\|59709473 | Pyruvate dehydrogenase kinase 1 precursor | 18.2 | 4 | PM |
| gi\|694003 | Pyruvate dehydrogenase kinase 2 subunit p45 | 20.9 | 2 | PM |
| gi\|61889071 | RAB10, member RAS oncogene family | 15.5 | 3 | PM |
| gi\|61889071 | RAB10, member RAS oncogene family | 29.0 | 4 | MT |
| gi\|158341664 | RAB1B, member RAS oncogene family | 68.2 | 10 | PM |
| gi\|158341664 | RAB1B, member RAS oncogene family | 67.7 | 8 | MT |
| gi\|149061036 | RAB2, member RAS oncogene family | 23.5 | 3 | MT |
| gi\|149029654 | RAB5B, member RAS oncogene family (predicted), isoform CRA_c | 16.3 | 2 | PM |
| gi\|149029654 | RAB5B, member RAS oncogene family (predicted), isoform CRA_c | 11.2 | 2 | MT |
| gi\|165970759 | Rab5c protein | 19.9 | 4 | MT |
| gi\|54114993 | RAP1A, member of RAS oncogene family precursor | 54.9 | 5 | PM |
| gi\|54114993 | RAP1A, member of RAS oncogene family precursor | 49.5 | 6 | MT |
| gi\|157819711 | Ras homolog gene family, member T1 | 7.3 | 3 | MT |
| gi\|206555 | Ras protein | 45.3 | 7 | PM |
| gi\|56605840 | Ras-related C3 botulinum toxin substrate 2 (rho family, small GTP binding protein Rac2) | 12.0 | 2 | CS |
| gi\|51338716 | Ras-related protein Rab-1A | 41.0 | 6 | PM |
| gi\|51338716 | Ras-related protein Rab-1A | 55.1 | 6 | MT |
| gi\|81884468 | Ras-related protein Rab-21 | 18.4 | 3 | PM |
| gi\|81884468 | Ras-related protein Rab-21 | 20.6 | 2 | MT |
| gi\|54038996 | Ras-related protein Ral-A | 27.2 | 2 | PM |

| # Accession | Protein name | % Cov | Peptides | Fraction |
|---|---|---|---|---|
| gi\|149025373 | rCG20813 | 50.9 | 4 | PM |
| gi\|149025373 | rCG20813 | 63.2 | 4 | MT |
| gi\|149016683 | rCG22622 | 27.3 | 3 | CS |
| gi\|149016272 | rCG23940, isoform CRA_g | 12.0 | 2 | PM |
| gi\|149016272 | rCG23940, isoform CRA_g | 26.5 | 1 | MT |
| gi\|149022203 | rCG26319 | 43.7 | 7 | PM |
| gi\|149022203 | rCG26319 | 45.0 | 9 | MT |
| gi\|149044044 | rCG27771, isoform CRA_b | 59.1 | 22 | CS |
| gi\|149024343 | rCG30666, isoform CRA_a | 6.5 | 1 | MT |
| gi\|149024657 | rCG31394, isoform CRA_b | 10.9 | 2 | CS |
| gi\|149052642 | rCG33456, isoform CRA_c | 67.1 | 35 | CS |
| gi\|149054052 | rCG33529, isoform CRA_c | 28.5 | 2 | PM |
| gi\|149054052 | rCG33529, isoform CRA_c | 14.6 | 1 | MT |
| gi\|149053564 | rCG35210 | 28.3 | 2 | CS |
| gi\|149019918 | rCG36507, isoform CRA_b | 9.8 | 3 | CS |
| gi\|149050264 | rCG36968, isoform CRA_c | 13.3 | 3 | MT |
| gi\|149035969 | rCG38845, isoform CRA_b | 59.7 | 18 | PM |
| gi\|149035969 | rCG38845, isoform CRA_b | 69.4 | 16 | MT |
| gi\|149032791 | rCG41951, isoform CRA_a | 64.7 | 18 | PM |
| gi\|149032791 | rCG41951, isoform CRA_a | 69.0 | 19 | MT |
| gi\|149029697 | rCG42519, isoform CRA_a | 46.1 | 22 | CS |
| gi\|149029697 | rCG42519, isoform CRA_a | 42.1 | 22 | PM |
| gi\|149059204 | rCG44686, isoform CRA_a | 56.3 | 5 | PM |
| gi\|149039410 | rCG45400 | 61.4 | 20 | CS |
| gi\|149039134 | rCG45607, isoform CRA_a | 18.3 | 16 | MT |
| gi\|149058125 | rCG46430 | 42.1 | 7 | PM |
| gi\|149064445 | rCG46917, isoform CRA_f | 14.4 | 2 | PM |
| gi\|149062343 | rCG47621, isoform CRA_a | 24.9 | 3 | CS |
| gi\|149061629 | rCG47744, isoform CRA_c | 31.0 | 4 | PM |
| gi\|149061629 | rCG47744, isoform CRA_c | 15.5 | 2 | MT |
| gi\|149064836 | rCG49984, isoform CRA_a | 21.1 | 2 | MT |
| gi\|149056475 | rCG54610, isoform CRA_a | 30.9 | 7 | PM |
| gi\|149045627 | rCG55067 | 21.2 | 6 | CS |
| gi\|149046922 | rCG58516, isoform CRA_a | 13.2 | 3 | PM |

| # Accession | Protein name | % Cov | Peptides | Fraction |
|---|---|---|---|---|
| gi\|149017560 | rCG59263 | 17.0 | 2 | PM |
| gi\|149048221 | rCG62728, isoform CRA_b | 44.8 | 8 | PM |
| gi\|149016250 | rCG62892 | 17.0 | 2 | PM |
| gi\|149020707 | rCG62940 | 28.0 | 4 | PM |
| gi\|149068224 | rCG63717 | 30.0 | 3 | PM |
| gi\|149068224 | rCG63717 | 30.0 | 5 | MT |
| gi\|61740635 | Related RAS viral (r-ras) oncogene homolog 2 | 24.0 | 4 | PM |
| gi\|61740635 | Related RAS viral (r-ras) oncogene homolog 2 | 13.2 | 2 | MT |
| gi\|81864134 | Reticulon-3 | 7.9 | 3 | PM |
| gi\|197246916 | RGD1306917 protein | 22.8 | 2 | PM |
| gi\|197246916 | RGD1306917 protein | 26.5 | 2 | MT |
| gi\|197246361 | RGD1310159 protein | 18.5 | 5 | CS |
| gi\|81883710 | Rho GDP-dissociation inhibitor 1 | 54.9 | 5 | CS |
| gi\|736292 | Ribophorin I | 13.6 | 5 | PM |
| gi\|736292 | Ribophorin I | 12.9 | 4 | MT |
| gi\|71051098 | Ribosomal protein L17 | 26.6 | 3 | PM |
| gi\|71051098 | Ribosomal protein L17 | 28.3 | 3 | MT |
| gi\|89573867 | Ribosomal protein L18 | 17.0 | 3 | PM |
| gi\|710295 | Ribosomal protein L22 | 29.7 | 2 | MT |
| gi\|56090279 | Ribosomal protein L23 | 11.4 | 1 | MT |
| gi\|560493 | Ribosomal protein L24 | 22.9 | 3 | PM |
| gi\|560493 | Ribosomal protein L24 | 15.3 | 3 | MT |
| gi\|34849736 | Ribosomal protein L36 | 21.0 | 2 | MT |
| gi\|51980641 | Ribosomal protein L4 | 24.5 | 5 | PM |
| gi\|51980641 | Ribosomal protein L4 | 23.0 | 4 | MT |
| gi\|78214309 | Ribosomal protein L8 | 21.0 | 3 | PM |
| gi\|78214309 | Ribosomal protein L8 | 22.6 | 3 | MT |
| gi\|78126139 | Ribosomal protein S12 | 31.1 | 2 | PM |
| gi\|78126139 | Ribosomal protein S12 | 25.0 | 1 | MT |
| gi\|54261703 | Ribosomal protein S13 | 17.2 | 3 | PM |
| gi\|54261703 | Ribosomal protein S13 | 14.6 | 1 | MT |
| gi\|8394215 | Ribosomal protein S17 | 27.4 | 1 | PM |
| gi\|8394215 | Ribosomal protein S17 | 26.7 | 1 | MT |
| gi\|82654220 | Ribosomal protein S19 | 22.1 | 3 | PM |

| # Accession | Protein name | % Cov | Peptides | Fraction |
|---|---|---|---|---|
| gi\|82654220 | Ribosomal protein S19 | 31.7 | 3 | MT |
| gi\|78126159 | Ribosomal protein S2 | 13.3 | 2 | PM |
| gi\|56090271 | Ribosomal protein S20 | 16.8 | 2 | PM |
| gi\|56090271 | Ribosomal protein S20 | 26.9 | 2 | MT |
| gi\|483517 | Ribosomal protein S21 | 34.9 | 2 | PM |
| gi\|483517 | Ribosomal protein S21 | 42.2 | 4 | MT |
| gi\|5924385 | Ribosomal protein S271 | 14.3 | 2 | PM |
| gi\|57164151 | Ribosomal protein S3 | 32.5 | 7 | PM |
| gi\|57164151 | Ribosomal protein S3 | 25.9 | 4 | MT |
| gi\|8394221 | Ribosomal protein S3a | 27.7 | 7 | MT |
| gi\|56090273 | Ribosomal protein S4, X-linked | 28.1 | 4 | PM |
| gi\|56090273 | Ribosomal protein S4, X-linked | 17.9 | 4 | MT |
| gi\|8394224 | Ribosomal protein S6 | 12.9 | 3 | PM |
| gi\|8394224 | Ribosomal protein S6 | 37.4 | 4 | MT |
| gi\|71795613 | Ribosomal protein, large P2 | 67.0 | 5 | PM |
| gi\|71795613 | Ribosomal protein, large P2 | 67.0 | 4 | MT |
| gi\|67678203 | Rpl6 protein | 26.2 | 6 | PM |
| gi\|67678203 | Rpl6 protein | 28.5 | 6 | MT |
| gi\|54261550 | Rps16 protein | 25.8 | 4 | PM |
| gi\|54261550 | Rps16 protein | 10.1 | 2 | MT |
| gi\|165970894 | Rps5 protein | 13.7 | 1 | MT |
| gi\|189181710 | Ryanodine receptor 2, cardiac | 17.5 | 39 | PM |
| gi\|189181710 | Ryanodine receptor 2, cardiac | 18.2 | 33 | MT |
| gi\|206846 | S-adenosylmethionine synthetase (EC 2.5.1.6) | 12.7 | 2 | CS |
| gi\|149042663 | Sarcalumenin (predicted), isoform CRA_a | 55.2 | 38 | PM |
| gi\|149042663 | Sarcalumenin (predicted), isoform CRA_a | 53.1 | 29 | MT |
| gi\|149042664 | Sarcalumenin (predicted), isoform CRA_b | 15.6 | 2 | CS |
| gi\|157823585 | Sarcoglycan, α | 7.0 | 2 | PM |
| gi\|189083744 | Sarcomeric mitochondrial creatine kinase precursor | 71.8 | 80 | PM |
| gi\|189083744 | Sarcomeric mitochondrial creatine kinase precursor | 74.7 | 133 | MT |
| gi\|57303 | Sarcoplasmic reticulum 2+-Ca-ATPase | 63.0 | 105 | PM |
| gi\|57303 | Sarcoplasmic reticulum 2+-Ca-ATPase | 53.8 | 78 | MT |
| gi\|157786908 | SCAN domain containing 3 | 11.1 | 5 | CS |
| gi\|38197654 | Scavenger receptor class B, member 2 | 14.6 | 2 | PM |

| # Accession | Protein name | % Cov | Peptides | Fraction |
|---|---|---|---|---|
| gi\|38197654 | Scavenger receptor class B, member 2 | 13.2 | 1 | MT |
| gi\|149022245 | Secernin 3, isoform CRA_a | 16.0 | 3 | CS |
| gi\|81884646 | Secernin-2 | 15.1 | 3 | CS |
| gi\|274325671 | Secretory carrier membrane protein 3 | 18.3 | 2 | PM |
| gi\|81879451 | Selenium-binding protein 1 | 52.5 | 20 | CS |
| gi\|58865630 | Serine (or cysteine) peptidase inhibitor, clade C (antithrombin), member 1 | 30.8 | 7 | CS |
| gi\|2507388 | Serine protease inhibitor A3N | 22.3 | 4 | CS |
| gi\|51036655 | Serine protease inhibitor α 1 precursor | 54.5 | 23 | CS |
| gi\|51036655 | Serine protease inhibitor α 1 precursor | 16.3 | 4 | PM |
| gi\|543717 | Serine/threonine-protein phosphatase 2A 55 kDa regulatory subunit B α isoform | 10.5 | 4 | CS |
| gi\|49065778 | Serine/threonine-protein phosphatase PP1-α catalytic subunit | 14.9 | 3 | CS |
| gi\|57977275 | Serpine1 mRNA binding protein 1 | 17.6 | 4 | PM |
| gi\|57977275 | Serpine1 mRNA binding protein 1 | 22.2 | 3 | MT |
| gi\|55824765 | Serpinh1 protein | 9.4 | 3 | PM |
| gi\|81884184 | Serum deprivation-response protein | 26.1 | 8 | PM |
| gi\|157819445 | SET and MYND domain containing 1 | 17.4 | 3 | CS |
| gi\|193806001 | S-formylglutathione hydrolase | 39.4 | 7 | CS |
| gi\|78395043 | Similar to Basic FGF-repressed Zic-binding protein (mbFZb) | 23.1 | 4 | PM |
| gi\|78395043 | Similar to Basic FGF-repressed Zic-binding protein (mbFZb) | 25.8 | 5 | MT |
| gi\|149038409 | Similar to C50H11.1, isoform CRA_a | 14.6 | 2 | CS |
| gi\|149053707 | Similar to Hypothetical protein MGC18716, isoform CRA_a | 14.6 | 3 | CS |
| gi\|149067647 | Similar to tripartite motif protein 50 (predicted) | 13.4 | 3 | CS |
| gi\|149067647 | Similar to tripartite motif protein 50 (predicted) | 40.5 | 11 | PM |
| gi\|149067647 | Similar to tripartite motif protein 50 (predicted) | 21.2 | 4 | MT |
| gi\|417812 | Single-stranded DNA-binding protein, mitochondrial | 17.2 | 2 | PM |
| gi\|47718004 | Slc25a3 protein | 54.9 | 35 | PM |
| gi\|47718004 | Slc25a3 protein | 49.3 | 34 | MT |
| gi\|197246451 | Slmap protein | 18.8 | 11 | PM |
| gi\|197246451 | Slmap protein | 18.0 | 8 | MT |
| gi\|5107153 | Small zinc finger-like protein | 22.7 | 2 | CS |
| gi\|5107153 | Small zinc finger-like protein | 34.0 | 2 | PM |
| gi\|5107153 | Small zinc finger-like protein | 76.3 | 2 | MT |
| gi\|5107200 | Small zinc finger-like protein | 27.0 | 1 | MT |
| gi\|6467898 | Small zinc finger-like protein DDP2 | 28.9 | 2 | MT |

| # Accession | Protein name | % Cov | Peptides | Fraction |
|---|---|---|---|---|
| gi\|6981542 | Solute carrier family 16, member 1 (monocarboxylic acid transporter 1) | 15.4 | 7 | PM |
| gi\|6981542 | Solute carrier family 16, member 1 (monocarboxylic acid transporter 1) | 15.6 | 5 | MT |
| gi\|6980958 | Solute carrier family 2 , member 4 | 10.2 | 3 | PM |
| gi\|6980958 | Solute carrier family 2 , member 4 | 7.9 | 2 | MT |
| gi\|52138624 | Solute carrier family 25 (carnitine/acylcarnitine translocase), member 20 | 46.2 | 14 | PM |
| gi\|52138624 | Solute carrier family 25 (carnitine/acylcarnitine translocase), member 20 | 56.5 | 14 | MT |
| gi\|38014819 | Solute carrier family 25 (mitochondrial carrier; adenine nucleotide translocator), member 4 | 87.9 | 118 | PM |
| gi\|149053212 | Solute carrier family 25 , member 11, isoform CRA_b | 62.7 | 21 | PM |
| gi\|149053212 | Solute carrier family 25 , member 11, isoform CRA_b | 59.9 | 27 | MT |
| gi\|189027101 | Solute carrier family 25, member 42 | 16.7 | 2 | MT |
| gi\|50054324 | Solute carrier family 27, member 1 | 6.3 | 2 | PM |
| gi\|50054324 | Solute carrier family 27, member 1 | 8.8 | 2 | MT |
| gi\|78214331 | Solute carrier family 8, member 1 precursor | 7.1 | 2 | PM |
| gi\|78214331 | Solute carrier family 8, member 1 precursor | 4.6 | 2 | MT |
| gi\|158138513 | Solute carrier family 9 isoform 3 regulator 2 | 17.5 | 3 | PM |
| gi\|189491879 | Sorcin | 10.6 | 2 | CS |
| gi\|81884480 | Sorting and assembly machinery component 50 homolog | 44.8 | 13 | PM |
| gi\|81884480 | Sorting and assembly machinery component 50 homolog | 53.9 | 13 | MT |
| gi\|624918 | SP120 | 11.0 | 2 | CS |
| gi\|149044856 | Spectrin β 2, isoform CRA_a | 26.2 | 37 | PM |
| gi\|158533972 | Spectrin α, erythrocytic 1 | 29.0 | 39 | PM |
| gi\|61557085 | Spectrin β, non-erythrocytic 1 | 8.9 | 7 | MT |
| gi\|157824057 | SRA stem-loop-interacting RNA-binding protein | 21.6 | 2 | CS |
| gi\|72255527 | Stomatin (Epb7.2)-like 2 | 10.2 | 2 | PM |
| gi\|72255527 | Stomatin (Epb7.2)-like 2 | 14.5 | 4 | MT |
| gi\|116242506 | Stress-70 protein, mitochondrial | 47.7 | 26 | PM |
| gi\|116242506 | Stress-70 protein, mitochondrial | 37.0 | 18 | MT |
| gi\|116242506 | Stress-70 protein, mitochondrial | 48.8 | 34 | CS |
| gi\|54036435 | Stress-induced-phosphoprotein 1 | 19.0 | 5 | CS |
| gi\|52782765 | Succinate dehydrogenase [ubiquinone] flavoprotein subunit, mitochondrial | 75.9 | 58 | PM |
| gi\|52782765 | Succinate dehydrogenase [ubiquinone] flavoprotein subunit, mitochondrial | 13.4 | 4 | CS |
| gi\|209915614 | Succinate dehydrogenase complex, subunit B, iron sulfur (Ip) precursor | 66.7 | 20 | PM |
| gi\|209915614 | Succinate dehydrogenase complex, subunit B, iron sulfur (Ip) precursor | 70.2 | 24 | MT |
| gi\|556395 | Succinate semialdehyde dehydrogenase | 20.5 | 6 | CS |

| # Accession | Protein name | % Cov | Peptides | Fraction |
|---|---|---|---|---|
| gi\|556395 | Succinate semialdehyde dehydrogenase | 8.8 | 2 | PM |
| gi\|158749584 | Succinate-CoA ligase, ADP-forming, β subunit | 38.1 | 9 | MT |
| gi\|223634703 | Succinyl-CoA ligase [GDP-forming] subunit α, mitochondrial | 22.3 | 8 | PM |
| gi\|223634703 | Succinyl-CoA ligase [GDP-forming] subunit α, mitochondrial | 28.0 | 6 | MT |
| gi\|187469277 | Sucla2 protein | 61.9 | 23 | CS |
| gi\|187469277 | Sucla2 protein | 42.7 | 14 | PM |
| gi\|71681082 | Suclg2 protein | 30.5 | 11 | CS |
| gi\|71681082 | Suclg2 protein | 27.3 | 8 | PM |
| gi\|71681082 | Suclg2 protein | 10.7 | 3 | MT |
| gi\|57241 | Sulfated glycoprotein 2 | 19.0 | 3 | CS |
| gi\|74024923 | Sulfite oxidase precursor | 13.0 | 3 | CS |
| gi\|8394328 | Superoxide dismutase 1, soluble | 53.9 | 14 | CS |
| gi\|8394328 | Superoxide dismutase 1, soluble | 39.0 | 6 | PM |
| gi\|8394331 | Superoxide dismutase 2, mitochondrial precursor | 41.9 | 18 | CS |
| gi\|8394331 | Superoxide dismutase 2, mitochondrial precursor | 50.5 | 12 | PM |
| gi\|8394331 | Superoxide dismutase 2, mitochondrial precursor | 46.4 | 10 | MT |
| gi\|50927605 | Suppression of tumorigenicity 13 | 20.1 | 3 | CS |
| gi\|157823944 | Sushi domain containing 2 | 6.2 | 1 | PM |
| gi\|149067676 | Syntaxin 4A (placental), isoform CRA_d | 18.5 | 2 | PM |
| gi\|189181726 | Talin | 8.3 | 7 | CS |
| gi\|189181726 | Talin | 7.8 | 1 | MT |
| gi\|149032481 | Target of myb1 homolog (chicken), isoform CRA_a | 19.2 | 1 | CS |
| gi\|209529675 | Taxilin β | 19.4 | 4 | CS |
| gi\|4325177 | Thiopurine S-methyltransferase | 16.7 | 2 | CS |
| gi\|34849734 | Thioredoxin 1 | 35.2 | 4 | CS |
| gi\|166796823 | Thioredoxin domain containing 17 | 18.7 | 2 | CS |
| gi\|55250718 | Thioredoxin reductase 2 | 10.1 | 2 | CS |
| gi\|55250718 | Thioredoxin reductase 2 | 6.7 | 1 | PM |
| gi\|81916316 | Thioredoxin-like protein 1 | 16.6 | 3 | CS |
| gi\|83305808 | Thiosulfate sulfurtransferase | 16.8 | 2 | MT |
| gi\|83305808 | Thiosulfate sulfurtransferase | 45.1 | 8 | CS |
| gi\|78103212 | Thymosin β-4 | 54.6 | 4 | CS |
| gi\|73918915 | TIM21-like protein, mitochondrial | 20.0 | 6 | PM |
| gi\|73918915 | TIM21-like protein, mitochondrial | 25.3 | 6 | MT |

| # Accession | Protein name | % Cov | Peptides | Fraction |
|---|---|---|---|---|
| gi\|84781723 | TNF receptor-associated protein 1 precursor | 24.1 | 8 | CS |
| gi\|84781723 | TNF receptor-associated protein 1 precursor | 10.2 | 3 | PM |
| gi\|84781723 | TNF receptor-associated protein 1 precursor | 11.3 | 3 | MT |
| gi\|38512111 | Tpi1 protein | 82.7 | 41 | CS |
| gi\|38512111 | Tpi1 protein | 43.6 | 7 | PM |
| gi\|38512111 | Tpi1 protein | 44.4 | 6 | MT |
| gi\|157817646 | TraB domain containing | 10.6 | 1 | PM |
| gi\|157817646 | TraB domain containing | 14.1 | 1 | MT |
| gi\|8393848 | Trans-2-enoyl-CoA reductase, mitochondrial precursor | 10.2 | 2 | CS |
| gi\|92090643 | Transaldolase | 27.6 | 3 | CS |
| gi\|51317294 | Transcription elongation factor B polypeptide 2 | 36.4 | 3 | CS |
| gi\|9963946 | Transcription factor A | 11.3 | 2 | PM |
| gi\|61556986 | Transferrin precursor | 60.0 | 62 | CS |
| gi\|61556986 | Transferrin precursor | 38.4 | 16 | PM |
| gi\|47605935 | Transforming protein RhoA | 21.2 | 4 | PM |
| gi\|47605935 | Transforming protein RhoA | 18.1 | 5 | MT |
| gi\|47605935 | Transforming protein RhoA | 21.8 | 2 | CS |
| gi\|603877 | Transgelin | 28.9 | 3 | CS |
| gi\|641973 | Transitional endoplasmic reticulum ATPase | 57.9 | 35 | PM |
| gi\|641973 | Transitional endoplasmic reticulum ATPase | 15.1 | 8 | MT |
| gi\|263511729 | Translational activator of cytochrome c oxidase 1 | 20.0 | 2 | CS |
| gi\|60688577 | Translocase of inner mitochondrial membrane 10 homolog (yeast) | 35.6 | 2 | PM |
| gi\|60688577 | Translocase of inner mitochondrial membrane 10 homolog (yeast) | 35.6 | 2 | MT |
| gi\|8394449 | Translocase of inner mitochondrial membrane 44 homolog precursor | 12.6 | 1 | CS |
| gi\|149015617 | Translocator of inner mitochondrial membrane 44, isoform CRA_a | 26.1 | 3 | MT |
| gi\|149015618 | Translocator of inner mitochondrial membrane 44, isoform CRA_b | 14.6 | 3 | PM |
| gi\|149030989 | Transmembrane 9 superfamily protein member 4, isoform CRA_d | 10.6 | 1 | PM |
| gi\|62906896 | Transmembrane emp24 domain-containing protein 10 | 28.3 | 2 | PM |
| gi\|57528337 | Transmembrane emp24 protein transport domain containing 9 | 10.6 | 2 | PM |
| gi\|81884639 | Transmembrane protein 109 | 7.4 | 1 | MT |
| gi\|261244910 | Transmembrane protein 11 isoform 2 | 12.5 | 1 | PM |
| gi\|81882927 | Transmembrane protein 126A | 18.4 | 2 | PM |
| gi\|81882927 | Transmembrane protein 126A | 13.3 | 2 | MT |
| gi\|157819597 | Transmembrane protein 143 | 12.5 | 2 | PM |

| # Accession | Protein name | % Cov | Peptides | Fraction |
|---|---|---|---|---|
| gi\|157819597 | Transmembrane protein 143 | 14.0 | 5 | MT |
| gi\|6851387 | Triadin 1 | 15.7 | 3 | PM |
| gi\|6851387 | Triadin 1 | 17.8 | 2 | MT |
| gi\|8650526 | Tricarboxylate carrier-like protein | 13.4 | 2 | MT |
| gi\|60688124 | Trifunctional enzyme subunit α, mitochondrial | 22.0 | 8 | CS |
| gi\|60688124 | Trifunctional enzyme subunit α, mitochondrial | 69.7 | 64 | PM |
| gi\|60688124 | Trifunctional enzyme subunit α, mitochondrial | 67.1 | 56 | MT |
| gi\|52353308 | Tropomyosin 3, γ isoform 1 | 49.0 | 7 | PM |
| gi\|92090646 | Tropomyosin α-1 chain | 46.8 | 9 | CS |
| gi\|8394469 | Troponin 1, type 3 | 29.9 | 4 | CS |
| gi\|77627992 | Troponin C type 1 (slow) | 23.0 | 3 | CS |
| gi\|6981666 | Troponin T type 2 (cardiac) | 21.4 | 5 | PM |
| gi\|55977470 | Tubulin α-1A chain | 18.9 | 4 | PM |
| gi\|55977470 | Tubulin α-1A chain | 43.5 | 11 | MT |
| gi\|55976173 | Tubulin α-1B chain | 59.2 | 19 | CS |
| gi\|81889864 | Tubulin α-4A chain | 31.5 | 10 | MT |
| gi\|81889864 | Tubulin α-4A chain | 54.9 | 19 | CS |
| gi\|81892373 | Tubulin β-2C chain | 21.4 | 4 | PM |
| gi\|81892373 | Tubulin β-2C chain | 19.8 | 7 | MT |
| gi\|56754676 | Tubulin β-5 chain | 55.4 | 17 | CS |
| gi\|210032365 | Tumor rejection antigen gp96 precursor | 12.7 | 8 | PM |
| gi\|210032365 | Tumor rejection antigen gp96 precursor | 19.2 | 9 | MT |
| gi\|9507243 | Tyrosine 3-monooxgenase/tryptophan 5-monooxgenase activation protein, β polypeptide | 37.0 | 8 | CS |
| gi\|6981710 | Tyrosine 3-monooxygenase/tryptophan 5-monooxygenase activation protein, η polypeptide | 56.9 | 12 | CS |
| gi\|9507245 | Tyrosine 3-monooxygenase/tryptophan 5-monooxygenase activation protein, γ polypeptide | 55.5 | 12 | CS |
| gi\|9507245 | Tyrosine 3-monooxygenase/tryptophan 5-monooxygenase activation protein, γ polypeptide | 16.6 | 3 | PM |
| gi\|6981712 | Tyrosine 3-monooxygenase/tryptophan 5-monooxygenase activation protein, θ polypeptide | 39.2 | 8 | CS |
| gi\|84028250 | Tyrosine-protein phosphatase non-receptor type 11 | 11.2 | 2 | CS |
| gi\|187469561 | Ube2l3 protein | 35.1 | 3 | CS |
| gi\|55741544 | Ubiquinol cytochrome c reductase core protein 2 precursor | 76.3 | 64 | PM |
| gi\|55741544 | Ubiquinol cytochrome c reductase core protein 2 precursor | 69.7 | 59 | MT |
| gi\|189011657 | Ubiquinol-cytochrome c reductase binding protein | 76.6 | 22 | PM |
| gi\|189011657 | Ubiquinol-cytochrome c reductase binding protein | 85.6 | 25 | MT |
| gi\|281427170 | Ubiquinol-cytochrome c reductase complex 7.2kDa protein | 57.8 | 6 | PM |

| # Accession | Protein name | % Cov | Peptides | Fraction |
|---|---|---|---|---|
| gi\|281427170 | Ubiquinol-cytochrome c reductase complex 7.2kDa protein | 51.6 | 3 | MT |
| gi\|186910239 | Ubiquinol-cytochrome c reductase, 6.4kDa subunit | 53.6 | 1 | PM |
| gi\|57114330 | Ubiquinol-cytochrome c reductase, Rieske iron-sulfur polypeptide 1 | 74.5 | 37 | PM |
| gi\|57114330 | Ubiquinol-cytochrome c reductase, Rieske iron-sulfur polypeptide 1 | 72.6 | 32 | MT |
| gi\|90111989 | Ubiquinone biosynthesis methyltransferase COQ5, mitochondrial | 19.0 | 3 | MT |
| gi\|90111992 | Ubiquinone biosynthesis protein COQ9, mitochondrial | 39.1 | 11 | CS |
| gi\|90111992 | Ubiquinone biosynthesis protein COQ9, mitochondrial | 23.7 | 3 | MT |
| gi\|8394502 | Ubiquitin C | 84.7 | 5 | PM |
| gi\|8394502 | Ubiquitin C | 55.1 | 4 | MT |
| gi\|68566104 | Ubiquitin carboxyl-terminal hydrolase isozyme L3 | 43.5 | 4 | CS |
| gi\|157819971 | Ubiquitin specific peptidase 5 | 24.5 | 6 | CS |
| gi\|205829267 | Ubiquitin thioesterase OTUB1 | 23.6 | 5 | CS |
| gi\|77417616 | Ubiquitin-conjugating enzyme E2 N | 28.3 | 3 | CS |
| gi\|157822205 | Ubiquitin-conjugating enzyme E2K | 19.5 | 2 | CS |
| gi\|157817518 | Ubiquitin-conjugating enzyme E2M (UBC12 homolog, yeast) | 25.7 | 2 | CS |
| gi\|51980281 | Ubiquitin-like modifier activating enzyme 3 | 6.5 | 1 | CS |
| gi\|81889667 | Ubiquitin-like modifier-activating enzyme 1 | 25.1 | 17 | CS |
| gi\|67078526 | UDP-glucose pyrophosphorylase 2 | 16.7 | 6 | CS |
| gi\|71043752 | UMP-CMP kinase 1 | 26.9 | 3 | CS |
| gi\|157820137 | Unc-45 homolog B | 16.9 | 4 | CS |
| gi\|81883788 | Uncharacterized protein C18orf19 homolog | 27.1 | 5 | PM |
| gi\|81883788 | Uncharacterized protein C18orf19 homolog | 38.5 | 5 | MT |
| gi\|34784756 | Unknown (protein for IMAGE:6890907) | 36.8 | 5 | MT |
| gi\|829026 | Unknown protein | 18.3 | 1 | PM |
| gi\|763179 | Unnamed protein product | 64.0 | 12 | CS |
| gi\|56929 | Unnamed protein product | 78.2 | 51 | CS |
| gi\|220659 | Unnamed protein product | 19.5 | 6 | CS |
| gi\|56336 | Unnamed protein product | 38.1 | 9 | CS |
| gi\|55985 | Unnamed protein product | 53.5 | 11 | PM |
| gi\|56929 | Unnamed protein product | 24.1 | 8 | PM |
| gi\|57139 | Unnamed protein product | 36.5 | 5 | PM |
| gi\|259435950 | Unnamed protein product | 59.5 | 53 | PM |
| gi\|57702 | Unnamed protein product | 13.8 | 1 | PM |
| gi\|57125 | Unnamed protein product | 23.9 | 6 | PM |

| # Accession | Protein name | % Cov | Peptides | Fraction |
|---|---|---|---|---|
| gi\|57129 | Unnamed protein product | 29.1 | 2 | PM |
| gi\|57294 | Unnamed protein product | 8.4 | 2 | PM |
| gi\|55628 | Unnamed protein product | 75.0 | 53 | PM |
| gi\|56905 | Unnamed protein product | 28.2 | 9 | PM |
| gi\|57127 | Unnamed protein product | 55.2 | 5 | MT |
| gi\|57294 | Unnamed protein product | 13.2 | 2 | MT |
| gi\|56929 | Unnamed protein product | 28.4 | 8 | MT |
| gi\|57125 | Unnamed protein product | 18.2 | 2 | MT |
| gi\|56905 | Unnamed protein product | 22.8 | 7 | MT |
| gi\|57139 | Unnamed protein product | 31.7 | 4 | MT |
| gi\|57129 | Unnamed protein product | 32.5 | 2 | MT |
| gi\|259435950 | Unnamed protein product | 64.7 | 56 | MT |
| gi\|81884656 | UPF0364 protein C6orf211 homolog | 16.6 | 4 | CS |
| gi\|81918167 | UPF0389 protein FAM162A | 51.0 | 10 | PM |
| gi\|81918167 | UPF0389 protein FAM162A | 54.2 | 6 | MT |
| gi\|81884088 | UPF0598 protein C8orf82 homolog | 34.9 | 2 | CS |
| gi\|81868653 | Up-regulated during skeletal muscle growth protein 5 | 55.2 | 4 | PM |
| gi\|81868653 | Up-regulated during skeletal muscle growth protein 5 | 55.2 | 6 | MT |
| gi\|73920806 | Valyl-tRNA synthetase | 6.1 | 3 | CS |
| gi\|4240462 | VAMP-associated protein A | 27.7 | 5 | PM |
| gi\|4240462 | VAMP-associated protein A | 14.9 | 2 | MT |
| gi\|4240464 | VAMP-associated protein B | 14.4 | 3 | PM |
| gi\|71043730 | Vanin 1 | 14.3 | 6 | PM |
| gi\|71043730 | Vanin 1 | 6.4 | 2 | MT |
| gi\|7381163 | Vasopressin-activated calcium-mobilizing receptor protein | 6.4 | 1 | CS |
| gi\|38051979 | Vdac1 protein | 94.3 | 72 | PM |
| gi\|76096306 | Vesicle amine transport protein 1 homolog (T californica) | 17.1 | 1 | PM |
| gi\|57480 | Vimentin | 21.7 | 8 | MT |
| gi\|205830826 | Vinculin | 6.5 | 4 | PM |
| gi\|205830826 | Vinculin | 16.1 | 7 | MT |
| gi\|205830826 | Vinculin | 36.2 | 26 | CS |
| gi\|76780264 | Vitronectin | 8.6 | 2 | PM |
| gi\|76780264 | Vitronectin | 7.1 | 2 | MT |
| gi\|8810245 | Voltage-dependent anion channel 1 | 91.2 | 69 | MT |

| # Accession | Protein name | % Cov | Peptides | Fraction |
|---|---|---|---|---|
| gi\|8810247 | Voltage-dependent anion channel 2 | 69.2 | 28 | PM |
| gi\|8810249 | Voltage-dependent anion channel 3 | 74.6 | 27 | PM |
| gi\|81910041 | WD repeat-containing protein 1 | 42.1 | 12 | CS |

The proteins whose expression levels were not significantly altered after morphine treatment (M) or withdrawal for 3 days ($M_W$-I) or 6 days ($M_W$-II) compared to controls were arranged according to their function into several groups. Number of accession (gi numbers from GenBank/EMBL/DDBJ databases) and fraction in which the protein was detected are quoted for each protein (CS, cytosol; PM, plasma membrane-enriched fraction; MT, mitochondria-enriched fraction). %Cov, the percentage of matching amino acids from identified peptides divided by the total number of amino acids in the sequence. Peptides, number of unique peptides per identified protein.
